# Disease-free monoculture farming by fungus-growing termites

**DOI:** 10.1038/s41598-019-45364-z

**Published:** 2019-06-19

**Authors:** Saria Otani, Victoria L. Challinor, Nina B. Kreuzenbeck, Sara Kildgaard, Søren Krath Christensen, Louise Lee Munk Larsen, Duur K. Aanen, Silas Anselm Rasmussen, Christine Beemelmanns, Michael Poulsen

**Affiliations:** 1 Section for Ecology and Evolution, Department of Biology, University of Copenhagen, Universitetsparken 15, Building 3, 2100 Copenhagen East, Denmark; 2 Leibniz Institute for Natural Product Research and Infection Biology – Hans Knöll Institute, Beutenbergstraße 11a, D-07745 Jena, Germany; 3 Section for Organismal Biology, Department of Plant and Environmental Sciences, University of Copenhagen, Thorvaldsensvej 40, 2000 Frederiksberg, Denmark; 4 Laboratory of Genetics, Wageningen University, Droevendaalsesteeg 1, 6708 PB Wageningen, The Netherlands; 5 DTU Bioengineering, Department of Biotechnology and Biomedicine, Technical University of Denmark, Søltofts Plads, Building 221, 2800 Kgs. Lyngby, Denmark; 6 Present Address: DTU Food, National Food Institute, Technical University of Denmark, Kemitorvet, Building 204, 2800 Kgs., Lyngby, Denmark; 7 Present Address: FMC Corporation, Genvej 2, 2970 Hørsholm, Denmark

**Keywords:** Metabolomics, Evolutionary ecology, Metagenomics, Symbiosis

## Abstract

Fungus-growing termites engage in an obligate mutualistic relationship with *Termitomyces* fungi, which they maintain in monocultures on specialised fungus comb structures, without apparent problems with infectious diseases. While other fungi have been reported in the symbiosis, detailed comb fungal community analyses have been lacking. Here we use culture-dependent and -independent methods to characterise fungus comb mycobiotas from three fungus-growing termite species (two genera). Internal Transcribed Spacer (ITS) gene analyses using 454 pyrosequencing and Illumina MiSeq showed that non-*Termitomyces* fungi were essentially absent in fungus combs, and that *Termitomyces* fungal crops are maintained in monocultures as heterokaryons with two or three abundant ITS variants in a single fungal strain. To explore whether the essential absence of other fungi within fungus combs is potentially due to the production of antifungal metabolites by *Termitomyces* or comb bacteria, we performed *in vitro* assays and found that both *Termitomyces* and chemical extracts of fungus comb material can inhibit potential fungal antagonists. Chemical analyses of fungus comb material point to a highly complex metabolome, including compounds with the potential to play roles in mediating these contaminant-free farming conditions in the termite symbiosis.

## Introduction

Monoculture farming faces a number of challenges, including increased susceptibility to pathogens in genetically homogenous crop populations^1,2^, as exemplified by Ireland’s Great Famine caused by potato blight (*Phytophthora infestans*). While diverse crops may provide more robust defence against invasive disease^3^, two major farming symbioses in nature – the New World fungus-farming ants and the Old World fungus-farming termites – maintain basidiomycete fungal crops in monoculture^4–7^. These are expected to be disease-prone because plant substrates harvested to manure the fungal crop may contain potential antagonists that could spread throughout the genetically homogeneous fungal gardens. In the ants, specialised *Escovopsis* spp. (Ascomycota) mycoparasites infect ant cultivars with potentially devastating impact on colonies^8^, but with the exception of stowaway ascomycete fungi in the genus *Pseudoxylaria* (Ascomycota: Xylariales)^9^, farming termites do not appear to suffer from specialised diseases.

**Figure 1:**
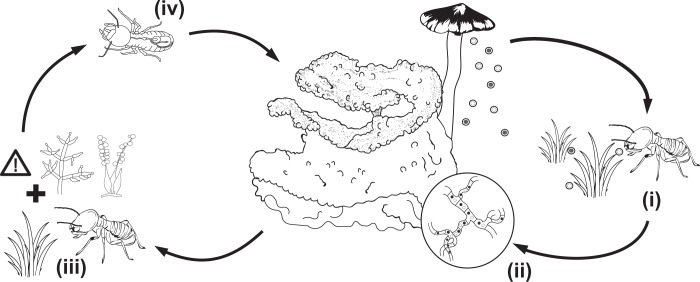
Right. A simplified schematic of the lifecycle of *Macrotermes natalensis* and *Odontotermes* sp. and *O*. cf. *badius*. In most termite species, *Termitomyces* spores are acquired from the environment (i) and brought back to incipient colonies, where spores fuse (ii) to form the dikaryotic mycelium characteristic of the growth within fungus combs. Left: Defence modes in farming termites. Termites forage for plant material (iii), which may contain potential antagonists such as *Trichoderma* or *Beauveria* (spore structures shown). Before being incorporated into the fungus comb, this plant material passes through the guts of young termite workers (iv). Any potential antagonists that pass through the gut unharmed must be suppressed through for example termite antimicrobial peptides, out-competition by *Termitomyces*, or antimicrobials of *Termitomyces* or bacterial origin.

Farming in termites originated thirty million years ago in a monophyletic group of 11 genera (330 described species) of higher termites (subfamily Macrotermitinae)^10–14^. The lignocellulose-digesting fungus cultivar (*Termitomyces*; Basidiomycota) is a source of termite nutrition^15,16^, providing access to otherwise inaccessible plant resources. It is cultivated in fungal gardens (combs), which are built with foraged plant material and asexual *Termitomyces* spores (in fungal nodules) that are mixed during termite gut passage^17,18^. Combs provide an optimal medium for fungal growth^16^, with *Termitomyces* growing rapidly to produce nutrient- and spore-rich nodules that are ingested by termite workers^19–21^. Workers then deposit further plant-spore mixture as new fungus comb on the older comb in a continuation of the fungal growth cycle (Fig. 1).

The decaying plant substrate that the termites harvest to manure *Termitomyces* is expected to harbour fungi that could compete with or antagonise *Termitomyces* or the termites (Fig. 1); however, only few have been reported, and these appear only to emerge after the termites have died or abandoned the mound^22–31^. There is thus no evidence of persistent infections in active combs or indications of specialised diseases, implying highly effective defences. These may include contaminant avoidance^32^ or physical removal by the termite host, termite-derived antimicrobial peptides^33,34^, and/or gut and comb symbiont-derived antimicrobials^28,29,35–37^. Compounds from *Termitomyces* may also be implicated, as non-*Termitomyces* fungal growth within combs is delayed by days following termite removal^38,39^. To profile fungal communities within fungus combs, we employ a combination of culture-dependent and -independent characterisation of fungi within combs of healthy termite nests. Subsequently, we take a metabolomics approach to examine the fungus-comb chemical environment and test its potential for suppression of antagonists *in vitro*.

## Results

### Culture-dependent and -independent identification of fungi within fungus combs

Nodule inoculations yielded solely *Termitomyces* in 83.3% of cases, with the remaining inoculations resulting in 23 fungal isolates from nine different genera (Table S1). Six events of fungus comb infections of laboratory colonies produced four *Trichoderma* and one *Fusarium* isolate (Table S1). Amplicon analyses from combs of 19 colonies (eight *M*. *natalensis*, six *Odontotermes* sp. and five *Odontotermes* cf. *badius*; Table S1) produced 702,804 quality-filtered ITS1 gene sequences from 454 pyro-sequencing and 408,491 quality-filtered ITS2 gene sequences from Illumina MiSeq, with 12,570–53,562 reads per sample after 454 sequence splitting, and 5,652–55,281 reads from MiSeq (Table S1). Coverage was judged sufficient based on rarefaction analyses (Fig. S1). 99% similarity clustering analysis yielded 560 and 14,095 OTUs from 454 and MiSeq, respectively, with 19–167 OTUs per 454-sequenced and 181–1,527 OTUs per MiSeq-sequenced comb sample. Taxonomic assignments yielded two fungal genera in the 454 (99.9% *Termitomyces*) and 21 in the MiSeq (99.9% *Termitomyces*) analyses (Tables S2 and S3). Non-*Termitomyces* genera were thus present in extremely low abundance, with *Preussia* (<0.001% relative abundance) being the only other identified genus in the 454 dataset, and the remaining non-*Termitomyces* reads (0.11%) being unassigned. In the MiSeq dataset, 20 non-*Termitomyces* fungal genera accounted for 0.07% relative abundance. *Pseudoxylaria* was detected in one *M*. *natalensis* and three *Odontotermes* sp. combs but accounted for only 0.03% of reads. It should be noted that this metabarcoding approach is only semi-quantitative as polyploidy, multiple ITS copies, DNA extraction biases etc. may bias community analyses. Although our mock fungal community analyses indicated that the primers used would detect the expected range of fungi (Fig. S2), the bias towards *Termitomyces* might in reality be less extreme than what we observed.

**Figure 2:**
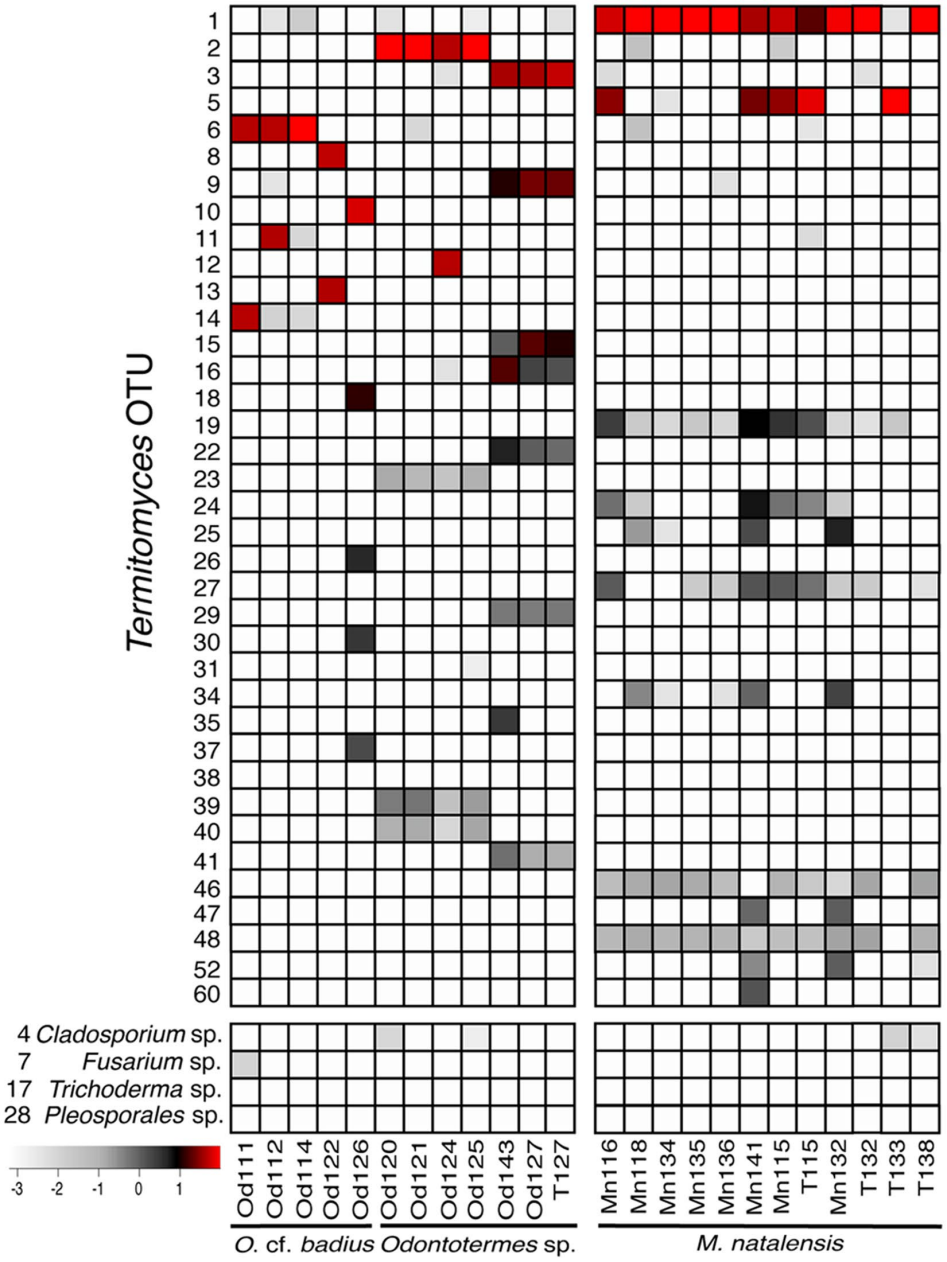
Heat maps showing the relative abundances of *Termitomyces* OTUs that accounted for >1% relative abundance per sample and the four other fungi identified in combs (fungus combs indicated with Od and Mn followed by colony code; pure cultures of *Termitomyces* indicated with T followed by colony code) in the MiSeq dataset for *Odontotermes* (left) and *M*. *natalensis* (right). The scale is a logarithmic calculation of the taxon read percentage out of the total number of quality-filtered and classified reads per sample.

Among the 561 and 14,002 *Termitomyces* OTUs in the 454 and MiSeq analyses, respectively, the most abundant OTUs (>1% relative abundance across datasets) are presented in heatmaps in Figs 2, S2 and S3. As expected, the analyses identified different *Termitomyces* OTUs in combs from different termite species; however, differences in the most abundant OTU were also apparent between combs from the same termite species (Figs 2, S2–S4). For example, the dominant *Termitomyces* OTUs in Od122 and Od126 combs were absent from other *O*. cf. *badius* colonies. Interestingly, three abundant OTUs from *Odontotermes* sp. Od127 were equally abundant and similar to the pure *Termitomyces* culture from that nest (Figs 2, S2 and S3). Furthermore, it is noteworthy that variants deviating from the one or two main types were found, but usually at low frequencies.

### Inter- and intra-colony variation of Termitomyces communities

Extraction and further analyses with 100% similarity clustering of *Termitomyces* MiSeq reads generated 47,492 *Termitomyces* variants across all fungus comb and pure culture samples (Table S3). At this threshold, the number of variants found ranged from 4,919 to 55,037 (average 22,427) per sample. While more *Termitomyces* variants were shared by fungus combs from colonies of different termite species, variants were apparent between conspecific colonies (Figs 2, S2 and S4; Table S3). For example, *O*. cf. *badius* colonies maintained different dominant *Termitomyces* variants. Differences between *Termitomyces* variants were present within single colonies. For example, the four most abundant of the 538 variants in the fungus comb from Mn115 had base pair differences at three positions, which were consistent with the findings for the pure culture (Table S3, Fig. S5). Similarly, Od127 showed differences between its eight dominant *Termitomyces* variants at 14 base pair positions, similar to the pure culture from that nest (Fig. S5), in which all but one of eight dominant *Termitomyces* variants were 100% identical to the dominant *Termitomyces* variants in the fungus comb.

### The effect of crude and fractionated fungus comb extracts on fungal contaminant growth

We evaluated the antifungal properties of extracts of comb material from six termite colonies (two of which were included in the fungal community analysis) against seven contaminants (four of which were detected at trace levels in the mycobiota analyses; Fig. 2). Five out of six crude acetonitrile (ACN) extracts inhibited the growth of *Trichoderma* sp. and three out of six *Beauveria bassiana* (Table S4, Fig. S7). Only the crude ACN extract from Od127 comb was inactive, while Od152 and Od167 extracts were active at 10 and 5 μg/μl (Table S4, Fig. S7). The crude *Macrotermes* ACN comb extracts showed varying degrees of activity at 10 and 5 μg/μl; in contrast, the six crude extracts prepared with a solution of acetonitrile/acetic acid (ACNAA) did not inhibit the growth of any of the tested fungi (Fig. S7; Table S4).

**Figure 3:**
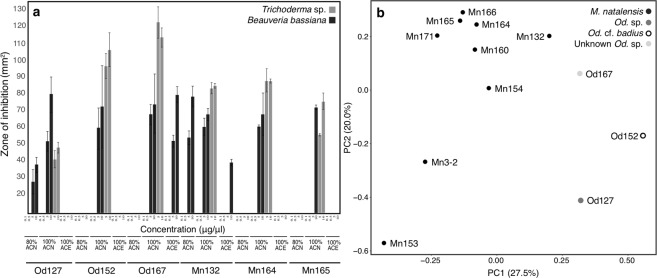
Antimicrobial activity assays and LCMS-PCA of fractionated fungus comb extracts. (**a**) Mean (±SE; n = 3) zones of inhibition (mm^2^) of *Trichoderma* sp. (grey) and *Beauveria bassiana* (black) when exposed to different concentrations of fractionated fungus comb extracts. Fractions were obtained by SPE eluting with 80% and 100% acetonitrile (ACN) and 100% acetone (ACE). Colonies of origin of fungus combs are indicated at the bottom of each panel. (**b**) PCA plot (PC1 vs PC2) of LCMS data for fractionated fungus comb extracts from 12 colonies of *M*. *natalensis*, *Odontotermes* sp. and *Odontotermes* cf. *badius*. Colonies labelled with colony ID numbers.

In order to narrow down the range of components potentially responsible for the observed activity, fractions from solid phase extraction (SPE) purification of the six tested extracts were also subjected to antifungal activity assays. Each of the active crude extracts also showed inhibition of *Trichoderma* sp. and *B*. *bassiana* in at least one of their corresponding fractions (Fig. 3; Table S4). The 100% ACN fraction of all comb extracts showed consistent inhibition of these two fungi at its highest concentration (10 μg/μl), followed by the next highest concentration (5 μg/μl) in all but one comb (Fig. 3; Table S4). While only the 100% ACN fractions inhibited *Trichoderma* sp., other fractions inhibited the entomopathogenic fungus *B*. *bassiana* (Fig. 3; Table S4). None of the crude comb extracts or fractions inhibited *Termitomyces* T112 or T115 (Table S4).

### LCMS/MS and principal component analysis (PCA) of fractionated fungus comb extracts

To analyse the overall metabolite composition and identify potential entities responsible for antimicrobial activity, tandem liquid chromatography-mass spectrometry (LCMS/MS) data were acquired for fractionated comb extracts prepared in the same manner, and for the same colonies, as those used for antifungal assays. These were supplemented with data from fractionated comb extracts for six additional *M*. *natalensis* colonies. Following data processing with MZmine 2^40^, a total of 66 chemical features (unique combinations of *m*/*z* and retention time values) were detected under positive electrospray ionisation (Table S5). Of these, 35 were detected in combs from both termite genera, while 26 were unique to *Macrotermes* and five unique to *Odontotermes*. In negative ion mode, 64 chemical features were identified, of which 24 were found in combs from both termite genera, 27 were unique to *Macrotermes* and 13 unique to *Odontotermes*. Dereplication using AntiBase and an in-house database of commercial reference substances revealed a broad range of putative hits for about 60% of all identified features within the positive ion mode and about 80% for the negative ion mode leaving a significant amount of mostly termite genera-specific chemical features unidentified. The predicted identities belong to a broad range of compound classes assigned to primary and secondary metabolism of plants and microorganisms. These include important monosaccharides, metabolites of the shikimic pathway, small and long chain fatty acids, bacterial autoinducer and quorum sensing molecules, as well as terpenes, terpene-derived compounds, and sterols of microbial or plant origin. Furthermore, several phenolic and polyhydroxylated aromatic compounds were putatively assigned, which could be redox mediators of fungal origin (Beemelmanns, unpublished data). In addition, we identified several typical fungal metabolites, such as terpenes, sterols (e.g., ergosterol and derivatives, carboxysterols or andrastins) and PKS-derived cyclic lactones (e.g., phomolides). Principal component analysis (PCA) revealed clustering largely according to termite genus, with 47.5% of the variance captured within the first two PCA components (Fig. 3).

### Inhibition of contaminant fungi by *Termitomyces*

To explore whether *Termitomyces* could be responsible for inhibition of other fungi, we conducted two *in vitro* interaction assays detailing inhibitory capabilities of a *M*. *natalensis* (T115) and an *Odontotermes* sp. (T112) *Termitomyces* (for details, see Supplementary Information). Most contaminant fungi grew less relative to controls when inoculated in direct contact with *Termitomyces*, with the exceptions of *Trichoderma* and *Fusarium* when tested against T115 (Fig. S8). Similar patterns were observed in the direct interactions with *Termitomyces* T112, but here *Fusarium* isolates never reached control growth levels, while a *Cladosporium* and a *Pleosporales* isolate grew more than twice as much in the presence of *Termitomyces* compared to controls (Fig. S8). In the indirect interaction assay, the *Trichoderma* isolates reached control rates in the presence of T115, a *Fusarium* isolate grew more than controls, while *Umbelopsis* and *Cunninghamella* had the same low growth rate as in the first assay (Fig. S8). In the T112 interactions, growth of one *Fusarium* isolate and both *Cladosporium* isolates was similar to in the direct assay; however, the other *Fusarium* isolate displayed increased growth and the *Pleosporales* sp. isolate exhibited reduced growth compared to the direct assay (Fig. S8).

## Discussion

### Termite fungus combs are essentially free of putative fungal pathogens

Non-*Termitomyces* fungi are extraordinarily rare within termite fungus combs, collectively accounting for <0.03% relative abundance based on the deep sequencing of mycobiotas from 19 nests. Only four of the contaminant isolates obtained from nodule and comb infections were detected in the amplicon sequencing, and even then, in extremely low abundances. Meta-barcoding data alone is only semi-quantitative as not all fungal species are picked up by this method to equal degrees and because polyploidy, heterokaryosis, differences in copy number of the ITS repeat and other factors may bias towards certain species. The mock community analyses to some extent help assess this potential bias, and we indeed amplified the four contaminant isolates that overlapped in our isolations and in the MiSeq of combs at expected high abundances in the mock communities. Several fungal genera, for example *Cladosporium*, *Trichoderma* and *Umbelopsis*, were obtained from isolations from multiple colonies of the three termite species, suggesting that they may enter combs relatively frequently, but likely only remain within combs as non-metabolising spores. These are frequently found in soil and decaying wood, and presumably enter the comb with the macerated plant substrate used to manure *Termitomyces*. Indeed, several have previously been reported from fungus-growing termites (e.g., *Trichoderma harzianum*^28^). Strikingly, *Pseudoxylaria* was encountered only at trace levels among amplicons. While *Pseudoxylaria* has not been reported from healthy functioning combs, it is often present in abandoned colonies^22,41^, and can usually readily be obtained if comb material is incubated in the absence of termites. Our findings thus support that co-occurring fungi such as *Pseudoxylaria* are stowaways that employ a sit-and-wait strategy^42^, emerging only when termites are absent.

### *Termitomyces* diversity within individual termite colonies

Fungus combs of Macrotermitinae are optimised for *Termitomyces* growth, but with the exception of studies in *M*. *natalensis*^4^ and *Odontotermes formosanus*^7^, there has been little effort to determine the diversity of *Termitomyces* within colonies. Fungal symbiont acquisition is mainly horizontal in newly founded colonies^43^, apart from in *Macrotermes bellicosus* and the genus *Microtermes*, where *Termitomyces* transmission is vertical and uniparental via one of the two founding reproductive alates^10,43^. Despite horizontal transmission, there is broad co-cladogenesis between termite hosts and fungus crops, implying some degree of association specificity^10^. Whether acquired horizontally or vertically, *Termitomyces* is propagated asexually within termite colonies^44^, and competitive exclusion through frequency-dependent selection ensures monoculture within individual nests^4^. Our findings confirm that individual termite colonies maintain a single *Termitomyces* strain and are consistent with previous work showing that *Termitomyces* grows as a heterokaryotic mycelium with two genetically different nuclei within both fungus combs and pure cultures^45,46^.

Although most nests had combs with two dominant *Termitomyces* ITS2 variants, three dominant variants were observed in both comb and pure-culture samples of T127 from an *Odontotermes* sp. nest (Fig. S5). Given that this culture was obtained from a single nodule, and therefore is unlikely to consist of two fungal strains, either the strain has three nuclei with unique ITS2 variants or one of the nuclei has two distinct ITS2 variants. The most plausible explanation, which is also consistent with previous findings^45^, is that combs contain a dikaryotic mycelium with one nucleus having more than one ITS variant. This is also consistent with the finding of small percentages of ITS variants deviating at a few positions from the most dominant types in other strains (Tables S3 and S4; Figure S5). Those findings beg for more study on the organisation of the ITS variants in heterokaryotic strains, either between the repeat units within nuclei or between genetically different nuclei within a single heterokaryon. The generation of single-nucleus (homokaryotic) isolates using protoplasting would allow a determination of ITS variants within nuclei, and shed light on whether the consistent low-abundance presence of variants in fungus combs and pure cultures are within-nucleus variants that never dominate ITS compositions.

### Termitomyces diversity between colonies

As expected, fungus combs from colonies of the same termite species harboured similar *Termitomyces* fungi, with our findings supporting the association of *M*. *natalensis* with a single *Termitomyces* species^10,47^. While most comb and isolate amplicon-sequencing data for *M*. *natalensis* indicated the presence of the same dominant ITS2 variant (OTU 1), one pure isolate was dominated by another variant (OTU 5). This variant was also abundant in several other *M*. *natalensis* nests and pure cultures, and almost always co-occurred with three other variants (OTUs 19, 24, and 27), which also supports that low-abundance variants are most likely present within single *Termitomyces* nuclei. As with *M*. *natalensis*, we found that the *Termitomyces* strains associated with *Odontotermes* sp. were dominated by the same ITS2 variants, suggesting that this termite species also likely associates with a single *Termitomyces* species, although this is based on only a few nests. If the unique ITS2 variant compositions are indeed indicative of different *Termitomyces* species, the five *O*. cf. *badius* species appear to associate with three different *Termitomyces* species, consistent with previous findings of low *Termitomyces* specificity in this termite species^47^.

### Inhibitory effects of Termitomyces and comb-residing bacteria

To investigate the extent to which *Termitomyces* has antifungal activities, we explored direct and indirect interactions between *Termitomyces* and contaminant fungi. We found that *Termitomyces* can have a negative effect on the growth of certain but not all tested fungi, but there was quite some variation in the interactions observed between the two *Termitomyces* isolates (Fig. S8). The lack of a negative effect on *Trichoderma* implies that the observed activity against this fungus (and likely *B*. *bassiana*) of fungus comb extracts (see below) is unlikely to be attributable to *Termitomyces* alone, although culture-based assays may not mimic natural comb conditions. A more parsimonious explanation is thus that antagonistic fungi are suppressed by antifungals produced by bacteria residing within combs, with the most likely candidates being Actinobacteria, which are abundant within combs^48^ and well-known antimicrobial producers^35,36,49^.

### The chemical environment of the fungus comb suppresses certain fungi

Compounds present in fungus combs could be responsible for suppression of potential antagonists. ACN extracts from five colonies showed antifungal activity against *Trichoderma* sp., which may compete with or antagonise *Termitomyces*, as well as the entomopathogenic fungus *B*. *bassiana*, suggesting that inhibitory properties may even target pathogens of the termite host. The comb chemical environment may thus suppress non-*Termitomyces* fungi, notably without negatively affecting *Termitomyces* (Table S4). To characterise this chemical environment, we performed LCMS/MS analyses complemented with PCA to assess the similarity of the chemical environments of fungus combs across different nests. Our analysis revealed some degree of clustering of colonies according to termite genera (Fig. 3), with the chemical profiles of samples collected from *Odontotermes* sp. and *O*. cf. *badius* nests resembling each other more closely than *M*. *natalensis* combs.

Careful examination of the LCMS/MS data revealed a complex assembly of detected chemical features in fungus combs. Subsequent comparison with the AntiBase natural compound database resulted in putative identifications of primary and secondary metabolites as shown in Table S5a–d. The identities of several metabolites (4-hydroxybenzaldehyde, azelaic acid, indole-3-carboxaldehyde, methylsuccinic acid, stearamide etc.) were verified using an in-house database of authentic commercial compounds, thereby comparing their retention times, high-resolution masses and fragmentation patterns. The most abundantly-detected features were universal primary metabolites (e.g., sugars, succinic acid derivatives), bacterial quorum sensing signals including several homoserine lactone derivatives, bacterial autoinducer signals, and plant and fungal derived fatty acids and sterols (e.g., cholesterols, ergosterols). While there is evidently an abundance of small primary metabolites present within fungal combs, several features attributed to secondary metabolism were also detected. A closer look at the assigned chemical features of comb extracts that inhibited fungal growth of contaminants revealed among others, the antioxidant ganosporeric acid and fungal-derived triterpenoid metabolites^50^, which have antibiotic properties. However, no general dominating antibiotic compounds present in all active comb samples could be identified.

In contrast, termite genera-specific chemical identities were often putatively assigned as secondary metabolites of diverse origin or were unassigned. Thus, it is tempting to speculate that genera-specific chemical features could be derived from differences in comb bacterial communities^48^ or *Termitomyces* associated with the termite genera. As our assay analysis revealed some degree of inhibition, we thus hypothesize that the observed antifungal activity is likely a result of the combination of bioactive compounds present within the comb material and cannot be attributed to single highly-abundant secondary metabolites. Overall the rate of true identification is illustrative of the challenges of metabolomics studies similar as the assignment of the observed activity to single compounds or compound groups. Pairing the metabolomic data with meta-transcriptomics data of healthy versus infected comb material or colonies directed at analysing secondary metabolite gene clusters and regulatory genes may help alleviate uncertainty surrounding compound identities.

The combined metabolome of the fungus comb may contribute to colony health not only by supporting *Termitomyces* growth but also suppressing antagonists within fungus combs, implying that the comb environment may act as a defence component in a series of consecutive defences that collectively allow the termites to sustainably maintain disease-free monoculture fungus farms. The termites themselves play key roles in keeping fungus combs healthy through hygienic practices, such as avoidance or physical removal of unwanted fungi and the secretion of antimicrobial peptides. Subsequent obligate gut passage of all incoming plant substrate for *Termitomyces* growth may provide an effective filter for removing substrate-dwelling competitors and antagonists, but unwanted microbes surviving gut passage should threaten comb health and select for the maintenance of comb defences. Collectively, the presence of sequential complementarity and multipartite defences in the symbiosis may be the key to the extremely robust broad-spectrum defence against a range of putative antagonists, but we have only just begun to unravel the relative importance of each of these defences and the antimicrobials involved.

## Material and Methods

### Culture-dependent isolation of cultivable fungi from fungus combs and PCR-based identification

Four *Odontotermes* cf. *badius*, three *Odontotermes* sp., one unknown *Odontotermes* sp., and four *Macrotermes natalensis* colonies were excavated from three sites in South Africa in 2011 (Table S1)^48^. Healthy fungus comb, workers, and soldiers were collected and brought back to the laboratory, where sub-colonies were set up in plastic containers at ambient humidity and 25 °C. For each nest, 12 nodules (asexual fruiting structures) of *Termitomyces* were aseptically placed on potato dextrose agar (PDA; 39 g/l) on the day of collection. Non-*Termitomyces* fungi growing from these inoculations were transferred to PDA, as were fungal infections of laboratory sub-colonies. These “contaminant” fungi have been deposited in the culture collection at the Department of Plant and Environmental Sciences, University of Copenhagen. We determined their identities by sequencing part of the Internal Transcribed Spacer (ITS) gene using ITS4 and ITS5 primers^51^.

### Samples for high throughput sequencing and chemical extraction of fungus combs

To characterise the fungal community composition within the fungus combs, material from 20 colonies of two termite genera were collected (Table S1). Samples were stored in RNAlater (Ambion, Inc., USA) at −20 °C until further use. To investigate the antifungal properties and chemical environment of fungus combs, material from 12 colonies of the same two genera (Table S1) was collected and stored at −20 °C until extractions were performed.

### ITS amplicon sequencing

#### DNA extraction and amplicon sequencing

The FastDNA SPIN Kit for Soil (MP Biomedicals, USA) was used for DNA extractions following the manufacturer’s instructions, with protocol modifications as in^48^. The ITS1 region was amplified for 454 pyrosequencing using ITS1-F (5′ CTTGGTCATTTAGAGGAAGTAA 3′) and ITS4-R (5′ TCCTCCGCTTATTGATATGC 3′) primers^51,52^ with additional sample specific multiplex identifier barcodes. PCR preparation, conditions and library preparations were performed as in^48^, with the exception that the annealing temperature was 58 °C. The ITS2 gene was chosen for Illumina MiSeq using ITS3-F (5′ GCATCGATGAAGAACGCAGC 3′) and ITS4-R (5′ TCCTCCGCTTATTGATATGC 3′) primers, and amplified using a dual-indexing sequencing strategy^53^. PCR preparation, conditions and library preparations were performed as for MiSeq pyrosequencing in^48^, with the exception that the annealing temperature was 56 °C. The use of both 454 pyrosequencing and Illumina MiSeq techniques reflects only the changing state of the art over the time period in which this work was performed. Five cultures of *Termitomyces* were included to compare the genetic makeup of pure cultures and natural fungus combs, as were three fungal community mock samples, each to test whether the ITS region adequately captured community diversity. Mock 1 had equal amounts of *Cladosporium phaenocomae*, *Fusarium* sp., and *Trichoderma*; Mock 2 had equal amounts of *Cladosporium perangustum*, a *Pleosporales* sp. and *Alternaria*; and Mock 3 had an equal mixture of Mock 1 and 2.

#### Bioinformatic analyses

The raw 454 flowgrams were fed into QIIME v. 1.8.0^54^ while the MiSeq analysis was performed using MOTHUR v. 1.34.3^55^. In QIIME, the multiplexed reads were assigned to samples based on unique barcodes and sequences were clustered into OTUs based on sequence similarity (99%). Representative sequences for each OTU were assigned to phylotypes using the UNITE database, and unassigned sequences were subjected to a BLASTn search against the non-redundant (NR) database in NCBI to determine their identity. Alpha diversity was estimated and represented as rarefaction curves. In MOTHUR, the standard operating procedure described at http://www.mothur.org/wiki/MiSeq_SOP was followed^55^. High quality sequences were aligned against the UNITE database. Alignments were assigned to taxa with a confidence threshold of 80% and operational taxonomic units (OTUs) were calculated at 3% species level classification. Finally, rarefaction curves based on 97% sequence similarity cut-off were generated using R v. 3.1.0^56^. Community similarities based on Bray-Curtis distances were visualised in R v. 3.1.0. Further, *Termitomyces* MiSeq quality-filtered reads were extracted and fed into a similarity clustering analysis using ClustalW v. 2.1^57^, with MOTHUR classification functions modified to a 100% base pair similarity threshold. For each comb or *Termitomyces* culture, all sequences that were 100% identical were pooled as one *Termitomyces* variant, after which variants accounting for >0.1% relative abundance per sample were aligned in Geneious v. 10.0.8^58^.

### Chemical extraction of fungus comb

First, for extraction of fungus combs in acetonitrile (ACN), comb from 12 colonies (Table S8) was pulverised and extracted with hexane (10 ml/g dry weight) overnight at room temperature (RT) to remove fatty acids and improve signal intensity. The hexane was filtered off and the comb extracted again with acetonitrile (ACN, 10 ml/g dry weight) overnight at RT. The ACN was concentrated under reduced pressure to yield crude comb extracts, six of which (Mn132, Mn164, Mn165, Od127, Od152 and Od167) were sampled (3 mg) for antifungal assays (see below). The remainders of these extracts, as well as the other six crude extracts, were dissolved in 80% aqueous (aq.) ACN (5 ml) using sonication and fractionated using a CHROMABOND C18 SPE column (500 mg sorbent capacity, Macherey Nagel GmbH & Co. KG). Metabolites were eluted with 80% aq. ACN (3 cv), 100% ACN (3 cv) and 100% acetone (3 cv) (Table S2). Second, for extraction of fungus combs in acetonitrile/acetic acid (ACNAA), comb material from six colonies (Table S2) was pulverised and extracted with 50% aq. ACN containing 1% acetic acid (10 ml/g dry weight) overnight at RT. The solvent was filtered off and concentrated under reduced pressure to yield crude extracts (ACNAA), which were sampled (3 mg) to perform antifungal bioassays.

### *In vitro* antifungal bioassays of comb extracts

To assess the antifungal activity of crude and fractionated comb extracts, we performed growth inhibition assays against *Cladosporium phaenocomae*, *Pleosporales* sp., *Trichoderma* sp., *Fusarium oxysporum*, and *Beauveria bassiana* (Table S1) in addition to two *Termitomyces* strains (T112 from an *O*. cf. *badius* colony and T115 from a *M*. *natalensis* colony) to serve as controls. Target antagonist/competitor strains were chosen based on the detection of fungal OTUs belonging to these genera in low abundance in the mycobiota analyses. An exception was the entomopathogenic *Beauveria bassiana*, which was included to represent a potential antagonist of the termites themselves. Culturability was also a criterion in strain selection. Crude ACN and ACNAA extracts were dissolved in 50% aq. dimethyl sulfoxide (DMSO) to yield 10, 5, 1, 0.5, 0.3, 0.1 and 0.01 μg/μl solutions. SPE fractions of the ACN extracts were dissolved (50% DMSO) to yield 10, 5, 0.5 and 0.1 μg/μl solutions. In addition to a negative control of 50% DMSO against contaminant fungi, all comb extracts were also tested against *Termitomyces* sp. isolated from *O*. cf. *badius* colony Od112 (T112) and *M*. *natalensis* colony Mn115 (T115). For activity assays, 200 μl of a 7-day-old contaminant or *Termitomyces* broth was inoculated on PDA. After 20 min, 8 μl crude or fractionated extract were placed on the inoculated plates. All assays were performed in triplicate and were evaluated by measuring zones of inhibition using ImageJ^59^ two days after inoculation. This timepoint was considered to be ecologically relevant as, in a natural setting, termite behavioural defences (e.g., weeding to remove contaminant fungi^32^) would subsequently be expected to be in effect.

### LCMS/MS and PCA of fractionated ACN fungus comb extracts

High-resolution LCMS/MS data were acquired for the SPE fractions of all 12 ACN fungus comb extracts and the extracts of *Termitomyces* cultures. Samples (2 mg/ml, 100% MeOH, 1 μL injection volume) were analysed using an Agilent UHPLCMS system, consisting of a 1290 Infinity UHPLC (Agilent Technologies, Torrance, CA, USA) equipped with a Poroshell 120 phenyl-hexyl column (250 mm × 2.1 mm, 2.7 μm particles). The column was eluted using a linear gradient consisting of A: HPLCMS grade H_2_O + 20 mM formic acid (FA) and B: HPLCMS grade ACN + 20 mM FA. The gradient ran from 10–100% B over 15 min, followed by 100% B for 2 min, returning to 10% B over 0.1 min and equilibrated for 1.9 + 2.0 (post run time) min prior to the next injection. A constant flow of 0.35 ml/min was used, and the column maintained at 60 °C. This was coupled to a 6545 QTOF-MS equipped with Agilent Dual Jet Stream electrospray ion source, in which samples were analysed in both negative and positive polarity. Mass spectra were recorded at 10, 20 and 40 eV as centroid data for *m*/*z* 85–1700 in MS mode and *m*/*z* 30–1700 in MS/MS mode, with an acquisition rate of 10 spectra/s. The ESI source parameters were: drying gas temperature, 250 °C; gas flow, 8 l/min; nebuliser gas pressure, 40 psig; sheath gas temperature, 300 °C; sheath gas flow, 12 l/min; capillary voltage, 4000 V; nozzle voltage, 500 V. Mass drift was corrected using a lock mass solution infused simultaneously with the sample using a secondary nebulizer, using *m/z* 922 (Agilent HP-0921) and *m/z* 186 (tributylamine) in positive polarity while *m/z* 966 (Agilent HP-0921 + formate) was used in negative polarity. Sample order was randomised.

Raw LCMS/MS data for fungus comb SPE fractions were converted to mzXML format using the ProteoWizard msconvert tool^60^ before pre-processing in MZmine 2 v. 2.37^40^ (parameters summarised in Table S7). Data were first baseline corrected, followed by peak detection, isotopic peak grouping, peak alignment, filtering and gap filling. Peaks were searched against the AntiBase database^61^ using the custom database search feature. To check which features were excluded by filtering for peaks with a corresponding MS2 scan, that otherwise would have been detected, both the positive and negative ESI datasets were also processed with this filter unchecked. This yielded no new features for the ESI + datasets, and only 20 additional features for the ESI- datasets. These are summarised in a separate spreadsheet within Table S5.

Peak lists (Table S5a–d) exported in csv format were edited to remove features that were also detected in the solvent or SPE blanks. Peak areas equal to zero (i.e., not detected) were replaced with a positive number being half of the value of the smallest non-zero peak area in the dataset, before a centred log ratio transformation was performed with the clr function from the chemometrics package in R v. 3.4.1. Principal component analysis (PCA) was then performed with the prcomp function (stats package) and results were visualised with the autoplot function (ggplot2 package).

### *In vitro* effects of *Termitomyces* on contaminant growth

To explore the effect of *Termitomyces* on the growth of contaminant fungi, interactions were evaluated in all possible pairings between two *Termitomyces* isolates, one from *M*. *natalensis* Mn115 (T115) and one from *Odontotermes* sp. Od112 (T112), and 16 contaminant isolates, seven from *M*. *natalensis* nests and nine from *Odontotermes* nests, in two separate assays. A first assay tested direct interactions by growing contaminant fungi on *Termitomyces*, while a second assay tested indirect interactions by measuring growth of contaminant fungi on a flipped PDA plate of a lawn of *Termitomyces*. *Termitomyces* nodules were macerated in 500 μl 0.8% NaCl solution with a sterile pestle, and the suspension was homogenised. Pure contaminant cultures were inoculated in PDB (24 g/l) and incubated for three days. *Termitomyces* inoculations were done by spreading 20 μl of *Termitomyces* suspension on PDA plates, which were incubated for three days before day zero, where the contaminants were inoculated. In the first assay, 30 μl contaminant broth was placed in the middle and on top of the *Termitomyces* culture. In the second assay, on day zero, the three days old *Termitomyces* cultures were flipped to face the bottom of the plate and 30 μl of the contaminant broth was placed on the middle of the flipped PDA. For all combinations, we performed three replicates and growth was measured 5, 8, 11, 14, and 17 days after day zero using ImageJ^59^.

## Supplementary information

Supporting Information

Table S2

Table S3

Table S4

Table S5

## Data Availability

The datasets generated and analysed during the current study are available in the Supplementary Information files of this article, and in GenBank and Dryad repositories. ITS sequences have been deposited in GenBank (accession no. KJ817309-KJ817331), 454 and MiSeq data have been submitted to NCBI (SRR6856127-SRR6856172), and LCMS data in mzXML format are available through the Dryad data repository at datadryad.org (10.5061/dryad.t6t12).
