# Supplementary material for: Disease-free monoculture farming by fungus-growing termites
Source: Sci Rep. 2019 Jun 19;9:8819. doi: 10.1038/s41598-019-45364-z (PMC6584615; doi:10.1038/s41598-019-45364-z)
Supplement: Supplementary file 1 — Supporting Information [file 41598_2019_45364_MOESM1_ESM.docx]

**Supplementary Information**

**for**

**Disease-free monoculture farming by fungus-growing termites**

Saria Otani^1†, ‡^, Victoria L. Challinor^1†^, Nina B. Kreuzenbeck^2^, Sara Kildgaard^1^, Søren Krath Christensen^1^, Louise Lee Munk Larsen^3§^, Duur K. Aanen^4^, Silas Anselm Rasmussen^5^, Christine Beemelmanns^2^ and Michael Poulsen^1*^

^1^Section for Ecology and Evolution, Department of Biology, University of Copenhagen, Universitetsparken 15, Building 3, 2100 Copenhagen East, Denmark.

^2^Leibniz Institute for Natural Product Research and Infection Biology – Hans Knöll Institute, Beutenbergstraße 11a, D-07745 Jena, Germany.

^3^Section for Organismal Biology, Department of Plant and Environmental Sciences, University of Copenhagen, Thorvaldsensvej 40, 2000 Frederiksberg, Denmark.

^4^Laboratory of Genetics, Wageningen University, Droevendaalsesteeg 1, 6708 PB, Wageningen, The Netherlands.

^5^DTU Bioengineering, Department of Biotechnology and Biomedicine, Technical University of Denmark, Søltofts Plads, Building 221, 2800 Kgs. Lyngby, Denmark.

^†^Contributed equally to this work

^‡^Current address: DTU Food, National Food Institute, Technical University of Denmark, Kemitorvet, Building 204, 2800 Kgs. Lyngby, Denmark.

^§^Current address: FMC Corporation, Genvej 2, 2970 Hørsholm, Denmark.

^*^Corresponding author

Michael Poulsen, Section for Ecology and Evolution, Department of Biology, University of Copenhagen, Universitetsparken 15, Building 3, 2100 Copenhagen East, Denmark, email: mpoulsen@bio.ku.dk, phone: +45 35330377.

**Table S1**. Termite species and colony codes, excavation locations and years (Mookgophong: S24 40 30.5 E28 47 50.4; ARC farm: S25 43 55.7 E28 14 08.2; Experimental farm: S25 43 55.7 E28 14 08.2; Lajuma: S23 02.218 E29 26.017; Iswepe: S26 50.163 E30 30.490; eManzana: S25 56.622 E30 35.869), number and identities of non-*Termitomyces* isolates and their closest matches in a BLASTn search, non-*Termitomyces* fungal infections occurring after colonies were set up in the lab, and the number of high-quality clean 16S rRNA gene sequences obtained from MiSeq and 454 pyrosequencing, respectively. Identification of *Odontotermes* termite species is based on COII sequencing when material was available (cf., Otani S, Hansen LH, Sørensen SJ, Poulsen M. 2016 Bacterial communities in termite fungus combs are comprised of consistent gut deposits and contributions from the environment. *Microb. Ecol.* **71**, 207-220, doi:10.1007/s00248-015-0692-6). Asterisks indicate colonies that were sampled for chemical extraction.

| **Termite colony** | **Location, year** | **Non-*Termitomyces* isolate number**  **and closest BLASTn match** | **Non-*Termitomyces* infections** | **MiSeq sequences** | **454 sequences** |
| --- | --- | --- | --- | --- | --- |
| *M. natalensis* Mn3-2* | Iswepe, 2016 | N/A | N/A | N/A | N/A |
| *O.* cf. *badius* Od111 | Experimental farm, 2011 | 1=*Cladosporium perangustum* (JF499836) (3 isolates) | Not tested | 20,214 (comb) | 41,105 (comb) |
| *O.* cf. *badius* Od112 | Experimental farm, 2011 | 9=PCR negative  3=*Cladosporium perangustum* (JF499836) | Not tested | 25,686 (comb) | 42,534 (comb) |
| Unknown *O*. sp. Od113 | Experimental farm, 2011 | 5=*Fusarium* sp. (HM631978)  13=*Cladosporium* *phaenocomae* (JF499838) | Not tested | N/A | N/A |
| *O.* cf. *badius* Od114 | Experimental farm, 2011 | 6=Uncultured Ascomycete endophyte (GQ999522)  7=*Coriolopsis* sp. (EU863193) | Not tested | 35,570 (comb) | 33,787 (comb) |
| *M. natalensis* Mn115 | Experimental farm, 2011 | 8=*Coriolopsis* sp. (EU863193) (1 isolate) | 22=*Trichoderma* sp. (JF831494) (1 isolate) | 17,595 (comb)  31,586 (culture) | 36,155 (comb) |
| *M. natalensis* Mn116 | Experimental farm, 2011 | 2=*Cladosporium perangustum* (JF499836) (2 isolates) | 18=*Fusarium oxysporum* (HQ651161) (1 isolate)  17=*Trichoderma* sp. (HQ607860) (1 isolate) | 15,306 (comb) | 53,562 (comb) |
| *M. natalensis* Mn117 | Mookgophong, 2011 | 14=*Umbelopsis isabellina* (JF303862) (2 isolates) | 21=*Trichoderma gamsii* (JF509737) (1 isolate) | N/A | N/A |
| *M. natalensis* Mn118 | Mookgophong, 2011 | 19=*Umbelopsis isabellina* (AB369912)  12=*Cunninghamella* *echinulata* var. *nodosa* (AF346407) | 16, 23=*Trichoderma gamsii* (JF509737)  (2 isolates) | 5,652 (comb) | 41,698 (comb) |
| *O.* sp. Od119 | Mookgophong, 2011 | 10=*Fusarium equiseti* (HQ607811)  20=Uncultured Ascomycete clone (GU722087) | N/A | N/A | N/A |
| *O.* sp. Od120 | ARC farm, 2011 | 11=*Umbelopsis isabellina* (JF303862) (1 isolate) | 24=*Hypocrea virens* (anamorph of *Trichoderma virens*) (HQ608079) (1 isolate) | 26,698 (comb) | 24,399 (comb) |
| *O.* sp. Od121 | ARC farm, 2011 | N/A | Not tested | 13,462 (comb) | 35,681 (comb) |
| *O.* cf. *badius* Od122 | ARC farm, 2011 | 4=*Pleosporales* sp. (HQ832821) (1 isolate) | N/A | 25,051 (comb) | 21,125 (comb) |
| *O.* sp. Od124 | ARC farm, 2011 | 15=*Alternaria alternata* (HQ263343) (1 isolate) | N/A | 25,107 (comb) | 51,922 (comb) |
| *O.* sp. Od125 | ARC farm, 2011 | N/A | Not tested | 55,281 (comb) | 47,237 (comb) |
| *O.* cf. *badius* Od126 | Experimental farm, 2013 | N/A | Not tested | 14,993 (comb) | 12,570 (comb) |
| *O.* sp. Od127* | Experimental farm, 2013 | N/A | Not tested | 23,181 (comb)  22,542 (culture) | 36,948 (comb) |
| *M. natalensis* Mn132* | Mookgophong, 2013 | N/A | Not tested | 10,205 (comb)  23,035 (culture) | 24,754 (comb) |
| *M. natalensis* Mn133 | ARC farm, 2013 | N/A | Not tested | 26,682 (culture) | N/A |
| *M. natalensis* Mn134 | ARC farm, 2013 | N/A | Not tested | 26,391 (comb) | 33,609 (comb) |
| *M. natalensis* Mn135 | ARC farm, 2013 | N/A | Not tested | 26,113 (comb) | 40,444 (comb) |
| *M. natalensis* Mn136 | Lajuma, 2013 | N/A | Not tested | 22,035 (comb) | 51,011 (comb) |
| *M. natalensis* Mn138 | Lajuma, 2013 | N/A | Not tested | 22,119 (culture) | N/A |
| *M. natalensis* Mn141 | Lajuma, 2013 | N/A | Not tested | 9,181 (comb) | 30,560 (comb) |
| *O.* sp. Od143 | ARC farm, 2013 | N/A | Not tested | 10,770 (comb) | 43,703 (comb) |
| *O.* cf. *badius* Od152* | ARC farm, 2016 | N/A | N/A | N/A | N/A |
| *M. natalensis* Mn153* | Experimental farm, 2016 | N/A | N/A | N/A | N/A |
| *M. natalensis* Mn154* | Experimental farm, 2016 | N/A | N/A | N/A | N/A |
| *M. natalensis* Mn160* | Experimental farm, 2016 | N/A | N/A | N/A | N/A |
| *M. natalensis* Mn164* | Experimental farm, 2016 | N/A | N/A | N/A | N/A |
| *M. natalensis* Mn165* | Mookgophong, 2016 | N/A | N/A | N/A | N/A |
| *M. natalensis* Mn166* | Mookgophong, 2016 | N/A | N/A | N/A | N/A |
| Unknown *O.* sp. Od167* | ARC farm, 2016 | N/A | N/A | N/A | N/A |
| *M. natalensis* Mn171* | eManzana, 2016 | N/A | N/A | N/A | N/A |
| Mock community for MiSeq #1 | |  |  | 27,843 | N/A |
| Mock community for MiSeq #2 | |  |  | 25,034 | N/A |
| Mock community for MiSeq #3 | |  |  | 35,029 | N/A |

**Table S2 (attached separately)**. Relative abundances (in percentages) of full fungal OTUs, identified with 454 pyrosequencing, in each fungus comb sample with taxonomical levels presented.

**Table S3 (attached separately)**. Relative abundances (in percentages) of full fungal OTUs, identified with Illumina MiSeq, in each fungus comb sample, *Termitomyces* pure culture and mock samples with taxonomical levels presented.

**Table S4 (attached separately)**. Antifungal activities of crude and fractionated fungus comb extracts when tested against *Trichoderma* sp., *Beauveria bassiana*, *Cladosporium phaenocomae*, *Pleosporales* sp., *Fusarium oxysporum* and *Termitomyces* strains isolated from an *O*. cf. *badius* colony (T112) and a *M. natalensis* colony (T115). Zones of inhibition, measured using ImageJ, are expressed in mm^2^.

**Table S5 (attached separately).** Peak lists for LCMS/MS analysis (ESI+ and ESI–) of fungus comb extracted in acetonitrile and fractionated by solid phase extraction (SPE), showing peak areas for detected chemical features (unique combinations of retention time and *m/z* values). ACN = acetonitrile; ACNAA = 50% aqueous acetonitrile containing 1% acetic acid; ACE = acetone. Peaks areas were summed across SPE fractions (included in separate sheets in this Excel workbook) prior to principal component analysis (PCA).

**Table S6.** Weights (g or mg as indicated) of crude fungus comb extracts, and material obtained after fractionation by solid phase extraction (SPE). ACN = acetonitrile; ACNAA = 50% aqueous acetonitrile containing 1% acetic acid; ACE = acetone.

| **Termite colony** | **Mn3.2** | **Mn132** | **Mn153** | **Mn154** | **Mn160** | **Mn164** | **Mn165** | **Mn166** | **Mn171** | **Od127** | **Od152** | **Od167** |
| --- | --- | --- | --- | --- | --- | --- | --- | --- | --- | --- | --- | --- |
|  |  |  |  |  |  |  |  |  |  |  |  |  |
| *Samples for antifungal assays* |  |  |  |  |  |  |  |  |  |  |  |  |
| Comb extracted in  ACN (g) | 25.0 | 50.0 | 25.1 | 50.0 | 25.0 | 50.1 | 50.2 | 25.1 | 25.1 | 25.0 | 24.9 | 25.0 |
| SPE 80% ACN (mg) | 262.8 | 327.5 | 302.6 | 676.4 | 221.5 | 449.2 | 517.6 | 213.8 | 191.6 | 285.4 | 163.0 | 216.2 |
| SPE 100% ACN (mg) | 12.1 | 18.2 | 16.5 | 12.3 | 11.6 | 12.8 | 19.9 | 7.5 | 10.3 | 7.9 | 7.5 | 11.6 |
| SPE 100% ACE (mg) | 10.4 | 13.6 | 16.1 | 16.7 | 16.7 | 13.6 | 17.8 | 7.6 | 8.0 | 6.8 | 4.1 | 6.6 |
| Comb extracted in ACNAA (g) | – | 50.0 | – | – | – | 50.1 | 50.0 | – | – | 25.0 | 25.0 | 25.0 |
|  |  |  |  |  |  |  |  |  |  |  |  |  |
| *Samples for LCMS/MS analysis* |  |  |  |  |  |  |  |  |  |  |  |  |
| Comb extracted in  ACN (g) | 10.0 | 10.0 | 10.0 | 10.0 | 10.0 | 10.0 | 10.0 | 10.0 | 10.0 | 10.0 | 10.0 | 10.0 |
| SPE 80% ACN (mg) | 97.1 | 61.7 | 118.2 | 93.7 | 61.8 | 65.0 | 80.5 | 59.8 | 58.0 | 106.8 | 31.8 | 70.3 |
| SPE 100% ACN (mg) | 4.6 | 5.6 | 7.4 | 3.1 | 4.8 | 2.7 | 3.0 | 2.6 | 2.8 | 5.5 | 1.9 | 3.1 |
| SPE 100% ACE (mg) | 2.6 | 4.4 | 6.6 | 2.2 | 3.0 | 2.1 | 2.5 | 2.2 | 2.0 | 5.1 | 1.9 | 2.0 |

**Table S7.** Parameters used for MZmine 2 pre-processing of positive and negative ion ESI LCMS/MS data for fractionated acetonitrile (ACN) fungus comb extracts (80% ACN, 100% ACN and 100% ACE fractions for ESI+ mode, 80% ACN and 100% ACN fractions for ESI–). ACN = acetonitrile, ACE = acetone.

| **Baseline correction** | **ESI+** | **ESI–** |  | **Isotopic peak grouping** | **ESI+** | **ESI–** |
| --- | --- | --- | --- | --- | --- | --- |
| *Chromatogram type* | BPC | - |  | m/z *tolerance* | 0.00 *m/z* or 20 ppm | 0.00 *m/z* or 20 ppm |
| *MS level* | 1 | *-* |  | *Retention time tolerance (min)* | 0.2 | 0.2 |
| *Use m/z bins* | Unchecked | *-* |  | *Monotonic shape* | Unchecked | Unchecked |
| *Baseline corrector* | Asymmetric | *-* |  | *Maximum charge* | 3 | 3 |
| *Smoothing* | 100 000 | *-* |  | *Representative isotope* | Most intense | Most intense |
| *Asymmetry* | 0.01 | - |  | **Peak alignment (Join aligner)** |  |  |
| **Peak detection (Mass detection)** |  |  |  | m/z *tolerance* | 0.00 *m/z* or 20 ppm | 0.00 *m/z* or 20 ppm |
| *Filters* | MS level 1 (then 2) | MS level 1 (then 2) |  | *Weight for* m/z | 75 | 75 |
| *Mass detector* | Centroid | Centroid |  | *RT tolerance* | 0.2 min | 0.2 min |
| *Noise level* | 1.0E3 (then 1.0E2) | 1.0E3 (then 1.0E2) |  | *Weight for RT* | 25 | 25 |
| *Mass list name* | masses | masses |  | **Peak filter (Peak list rows filter)** |  |  |
| *CDF filename* | Unchecked | Unchecked |  | *Minimum peaks in a row* | Unchecked | Unchecked |
| **Chromatogram builder** |  |  |  | *Minimum peaks in an isotope pattern* | Checked, 2 | Unchecked |
| *Scans* | MS level 1  Retention time 1.45-16.50 | MS level 1  Retention time 1.00-13.50 |  | *Keep only peaks with MS2 scan* | Checked | Checked |
| *Mass list* | masses | masses |  | *Reset the peak number ID* | Checked | Checked |
| *Min time span* | 0.01 | 0.01 |  | **Gap filling (peak finder)** |  |  |
| *Min height* | 3.0E3 | 3.0E3 |  | *Intensity tolerance* | 10% | 10% |
| *Mass tolerance* | 0.00 *m/z* or 20 ppm | 0.00 *m/z* or 20 ppm |  | m/z *tolerance* | 0.00 *m/z* or 20 ppm | 0.00 *m/z* or 20 ppm |
| **Chromatogram deconvolution** |  |  |  | *RT tolerance* | 0.3 min | 0.3 min |
| *Algorithm* | Baseline cut-off | Baseline cut-off |  | *RT correction* | Unchecked | Unchecked |
| *Min peak height* | 1.0E4 | 1.0E4 |  | **Custom database search** |  |  |
| *Peak duration range (min)* | 0.01-0.50 | 0.01-0.50 |  | *Field separator* | , | , |
| *Baseline level* | 1.0E3 | 1.0E3 |  | *Field order* | ID, *m/z*, Retention time (min), Identity, Formula | ID, *m/z*, Retention time (min), Identity, Formula |
| m/z *center calculation* | Auto | Auto |  | *Ignore first line* | Checked | Checked |
| m/z *range for MS2 scan pairing* | 0.02 | 0.02 |  | m/z *tolerance* | 0.00 *m/z* or 5 ppm | 0.00 *m/z* or 5 ppm |
| *RT range for MS2 scan pairing* | 0.2 | 0.2 |  | *Retention time tolerance* | 20.0 min | 20.0 min |


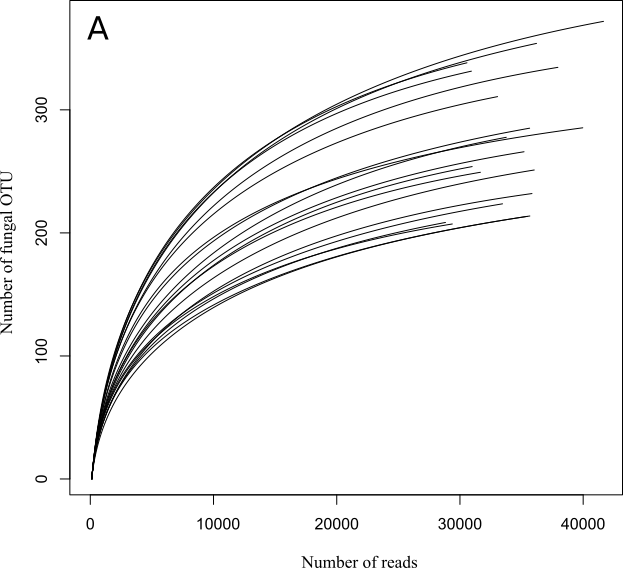

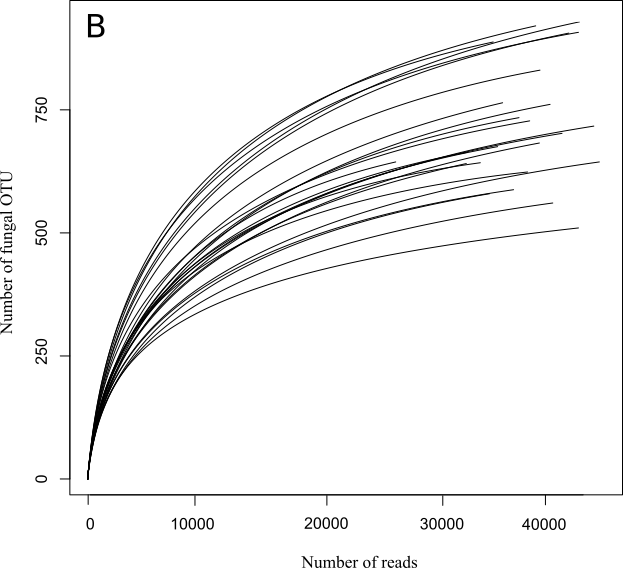


**Figure S1**. Rarefaction curves of the number of operational taxonomic units (OTUs) for fungal communities in each sample as assessed by (A) 454 pyrosequencing for fungus combs and (B) Illumina MiSeq for fungus combs, *Termitomyces* pure cultures, and mock samples. OTUs were delineated at 97% sequence similarity.

**Figure S2**. Fungal OTUs accounting for > 1% relative abundance in the entire Illumina MiSeq dataset. The horizontal axis lists termite colony of origin for fungus comb samples and *Termitomyces* pure cultures, along with the mock samples, while the vertical axis presents the identified fungal taxa. The coloured scale of the heat map represents fungal relative abundances within each mycobiota (for full results, see Table S3).

**Figure S3**. Fungal OTUs accounting for > 1% relative abundance in the entire 454 pyrosequencing dataset. The horizontal axis lists termite colony of origin for fungus comb samples, and the vertical axis presents the identified fungal OTU taxa, corresponding to different *Termitomyces* variants. The coloured scale of the heat map represents fungal relative abundances within each mycobiota (for full results, see Table S2).

**Figure S4**. Non-metric multidimensional scaling (NMDS) ordination plots of fungal communities based on Bray-Curtis distances for A) samples sequenced with 454 pyrosequencing and B) samples sequenced with Illumina MiSeq. The ordination displays the similarity of fungal communities between the different samples. Green represents *O.* cf. *badius* fungus comb samples, red *O.* sp., blue *M. natalensis* or *Termitomyces* isolated from *M. natalensis* colonies, and black the mock community samples.


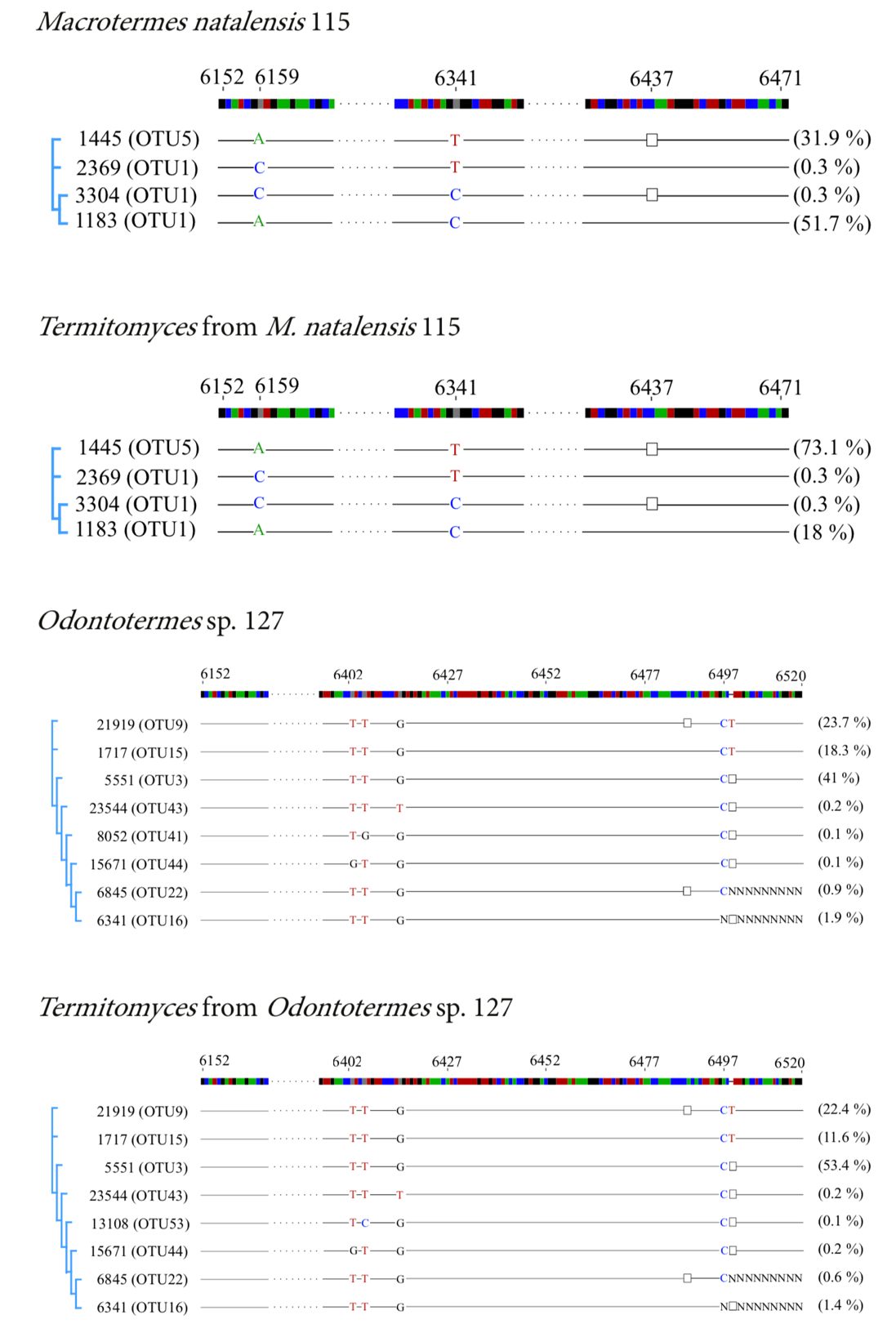


**Figure S5.** Clustering analyses of ITS2 gene sequences of *Termitomyces* variants within combs from two colonies (one per termite genus: *Macrotermes natalensis* 115 and *Odontotermes* sp. 127) and their respective pure culture isolates (*Termitomyces* from *Macrotermes natalensis* 115 and *Termitomyces* from *Odontotermes* sp. 127). The Maximum Likelihood trees on the left represent phylogenetic relationships between the different *Termitomyces* variants in its corresponding comb or pure culture, and the percentages on the right represent the relative abundances of each *Termitomyces* variant in its corresponding fungal community. The numbers on top of each cluster indicate the start and end positions of the variant OTUs in ITS2 and positions of the varying base pairs between variant OTUs in its corresponding fungal community. Unfilled squares indicate deletions.


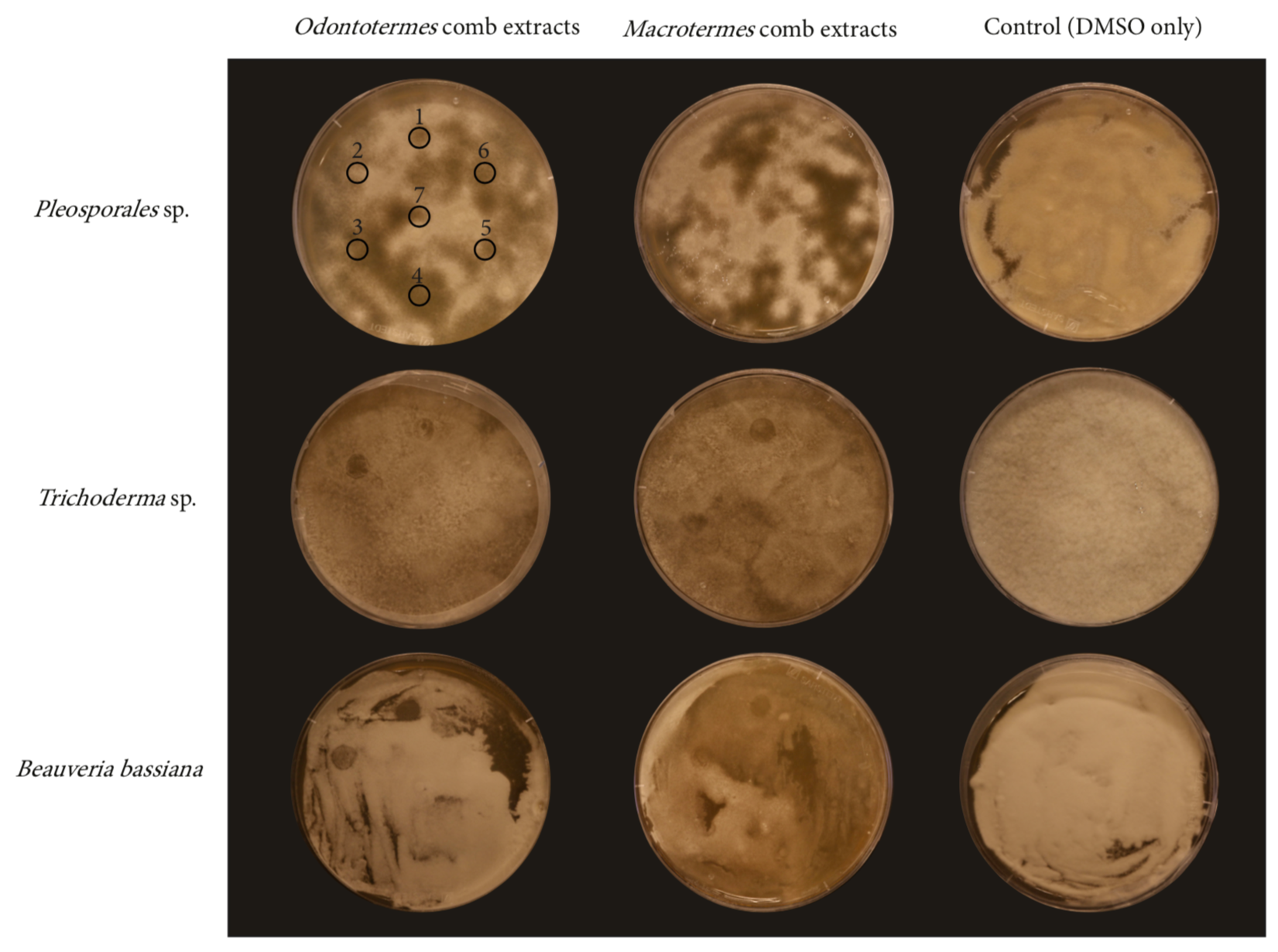


**Figure S6**. Representative plates of fungal growth inhibition assays performed with crude fungus comb extracts. The top left plate shows the assay setup, with numbers indicating the placement of comb extract solutions (8 μl each) with different concentrations. Solutions were prepared in 1:1 DMSO:H_2_O with the following concentrations: 10 μg/μl (1); 5 μg/μl (2); 1 μg/μl (3); 0.5 μg/μl (4); 0.3 μg/μl (5); 0.1 μg/μl (6); and 0.01 μg/μl (7). Assays were performed in triplicate.

**Figure S7**. Results of fungal growth inhibition assays, showing mean (±SE; n = 3) zones of inhibition (mm^2^) of *Beauveria bassiana* (red) and *Trichoderma*sp. (blue) when exposed to different concentrations of crude acetonitrile (ACN) extracts of fungus comb material. Colonies of origin of fungus combs are indicated at the bottom of each panel.

**
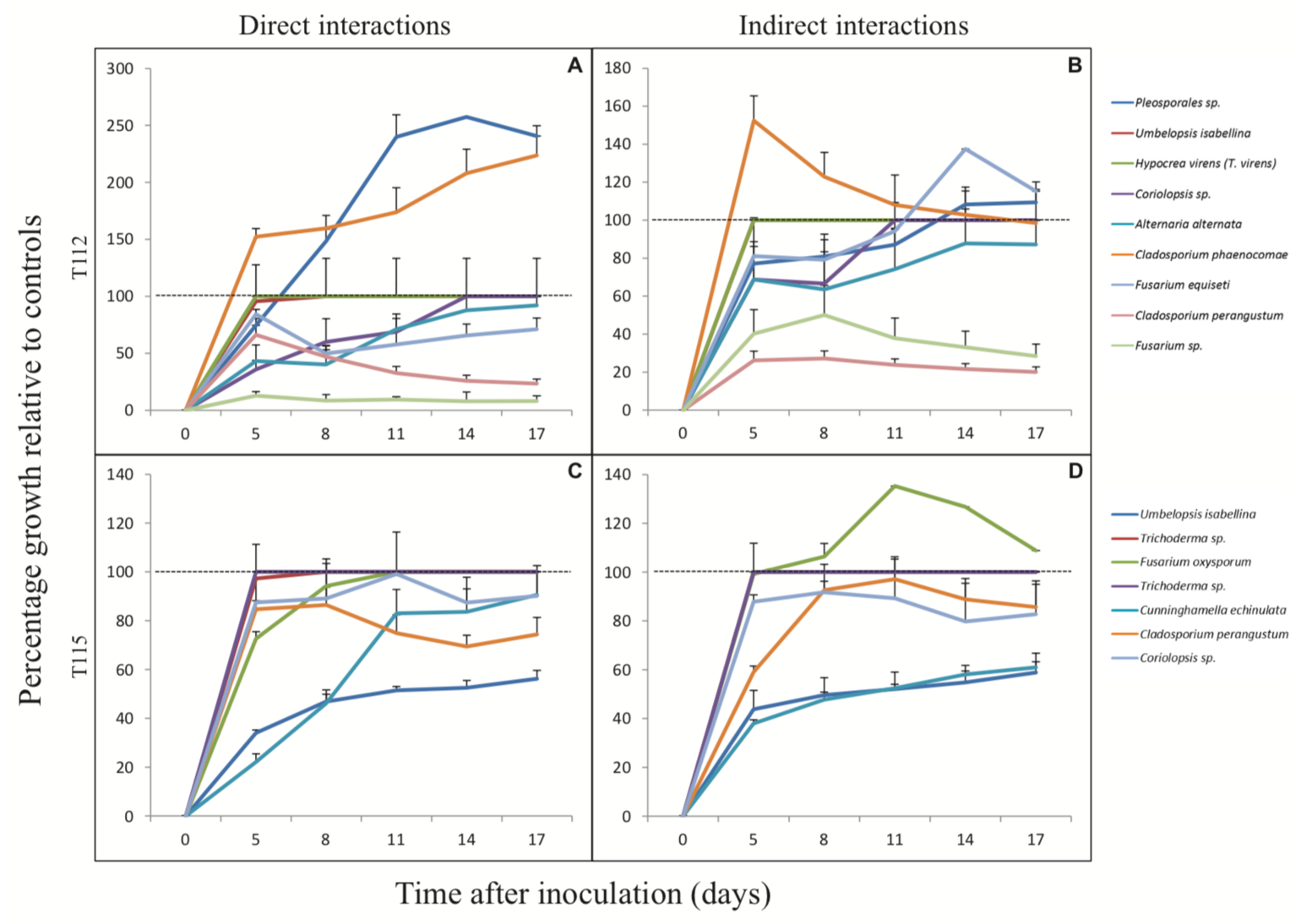
**

**Figure S8**. Mean±SE (n = 3) growth of contaminant fungi in *in vitro* interactions with *Termitomyces* isolate T115 from *M. natalensis* Mn115 (A,B) and *Termitomyces* isolate T112 from *Odontotermes* cf. *badius* Od112 (C,D) in either direct (A,C) or indirect interactions (B,D). Dashed lines indicate contaminant growth identical to when *Termitomyces* was absent (controls).


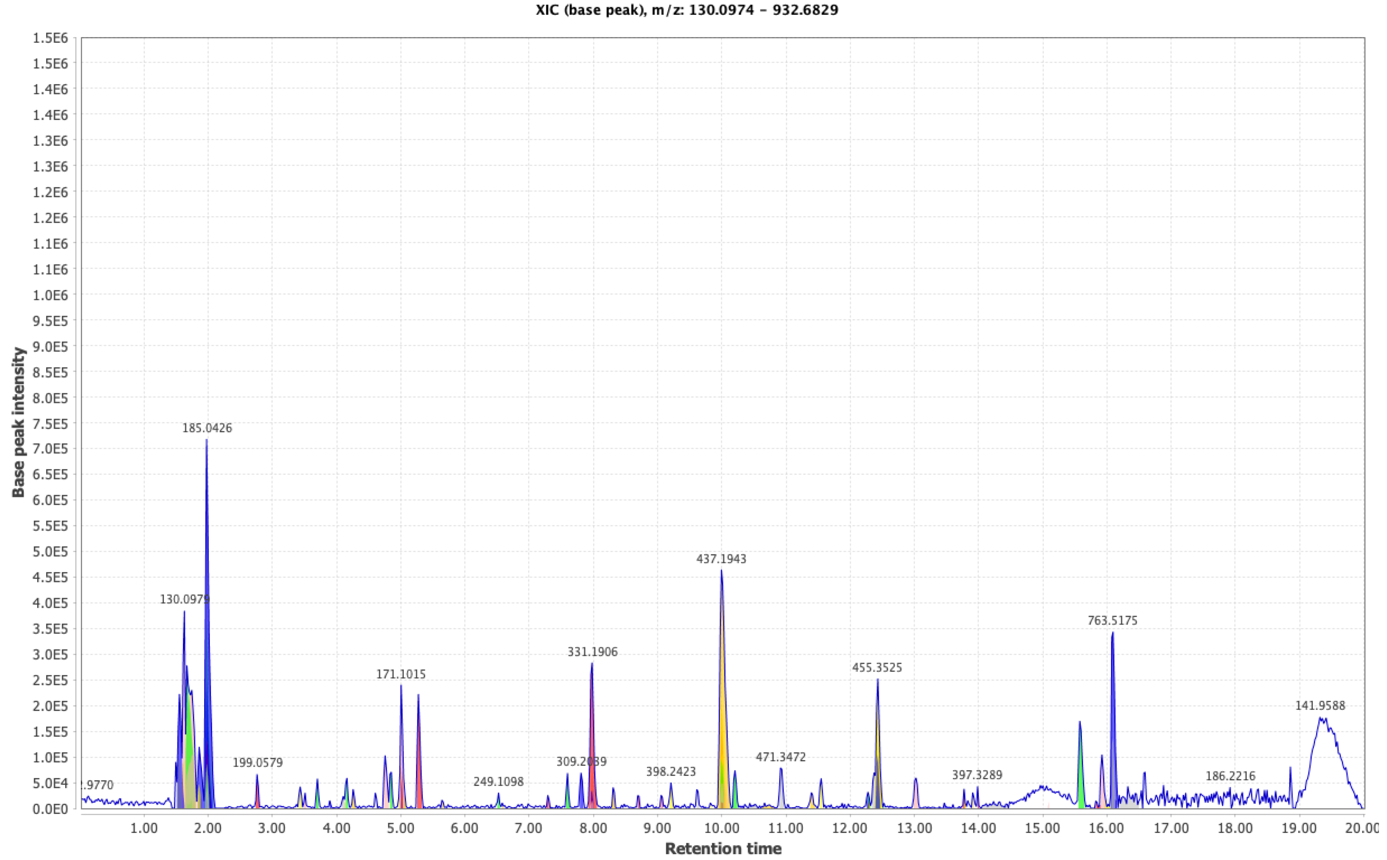


**Figure S9.** Base peak chromatogram (positive ESI) for fungus comb from colony Mn3.2 extracted in acetonitrile (ACN), 80% ACN fraction (retention time in min).


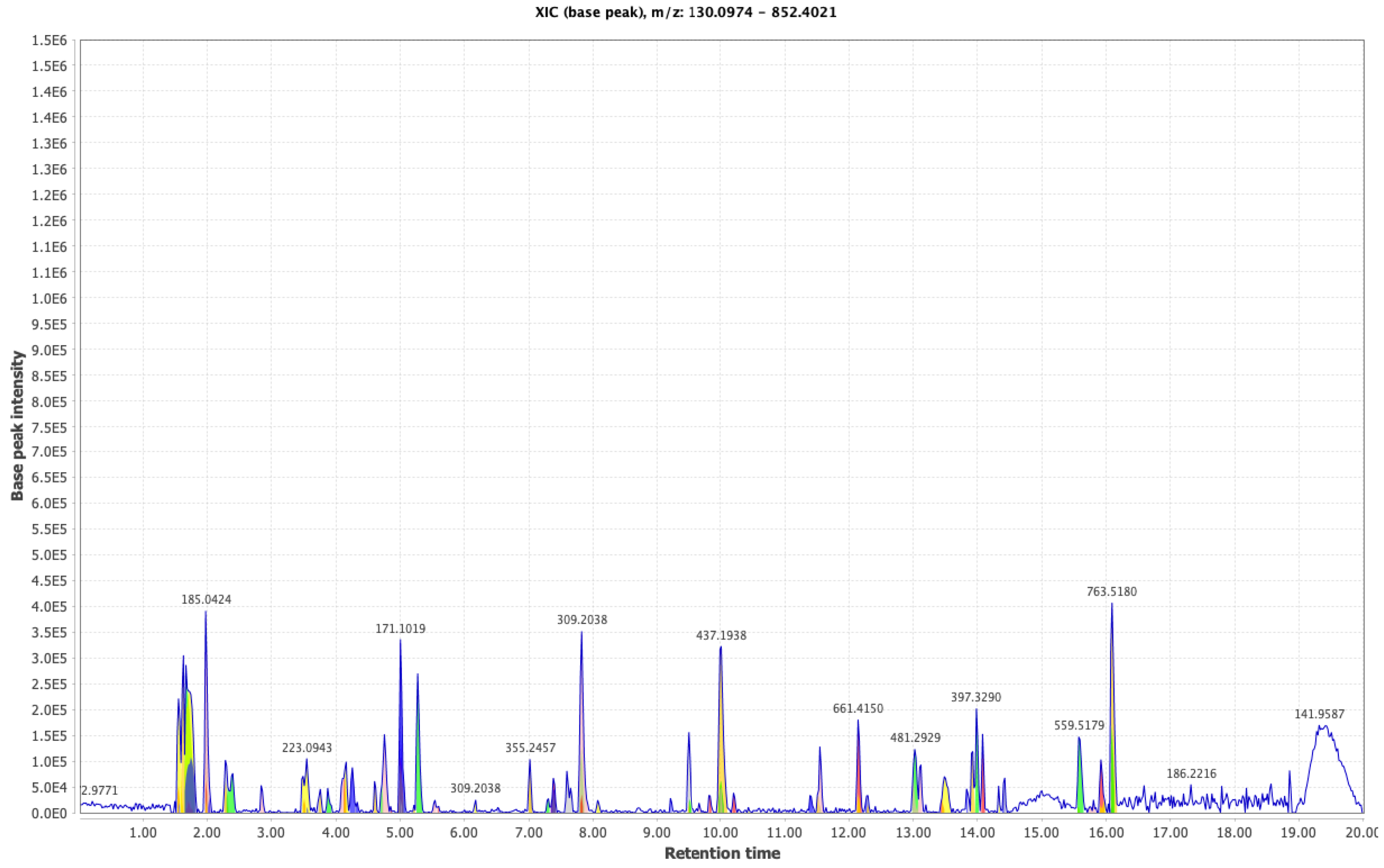


**Figure S10.** Base peak chromatogram (positive ESI) for fungus comb from colony Mn132 extracted in acetonitrile (ACN), 80% ACN fraction (retention time in min).


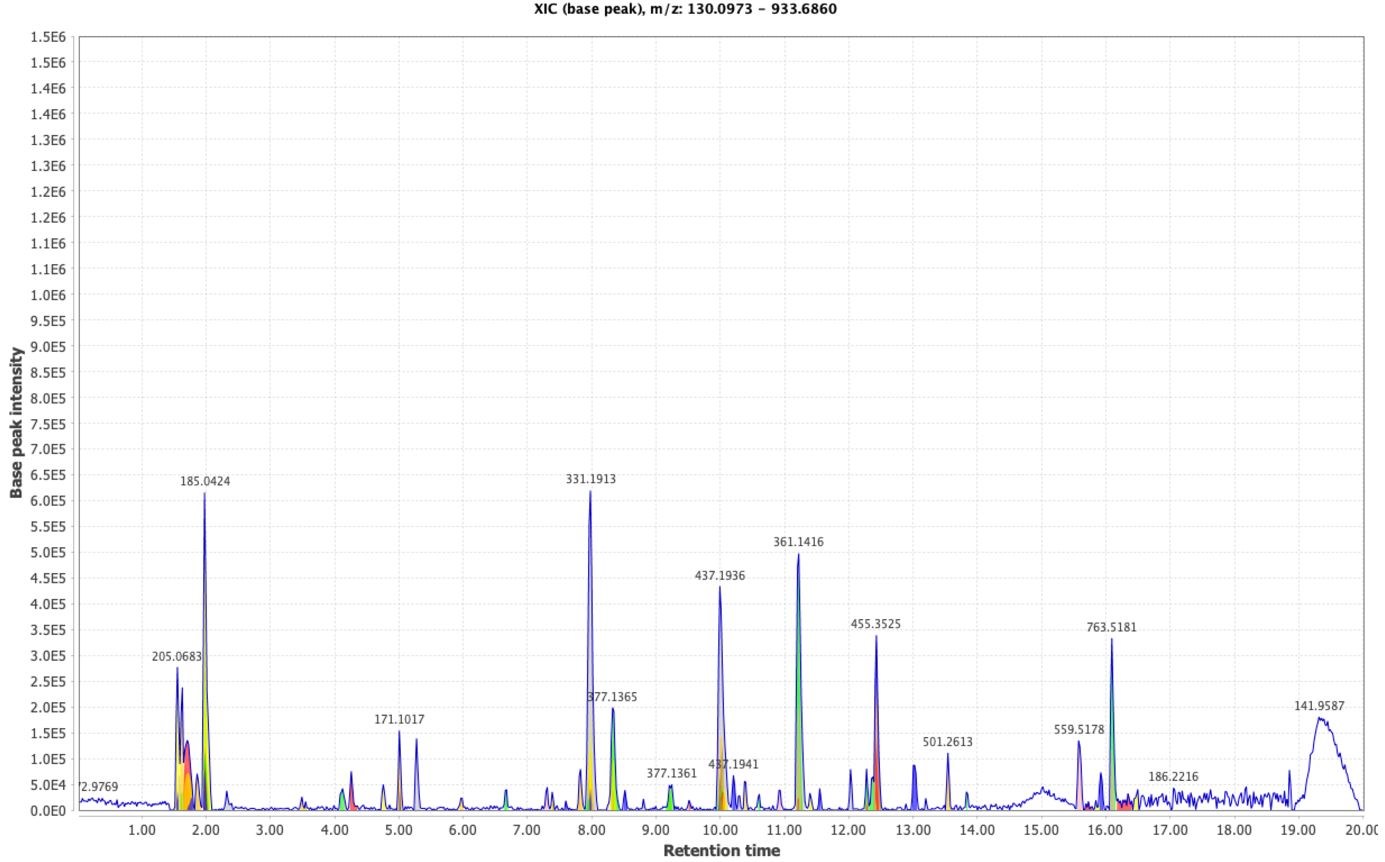


**Figure S11.** Base peak chromatogram (positive ESI) for fungus comb from colony Mn153 extracted in acetonitrile (ACN), 80% ACN fraction (retention time in min).


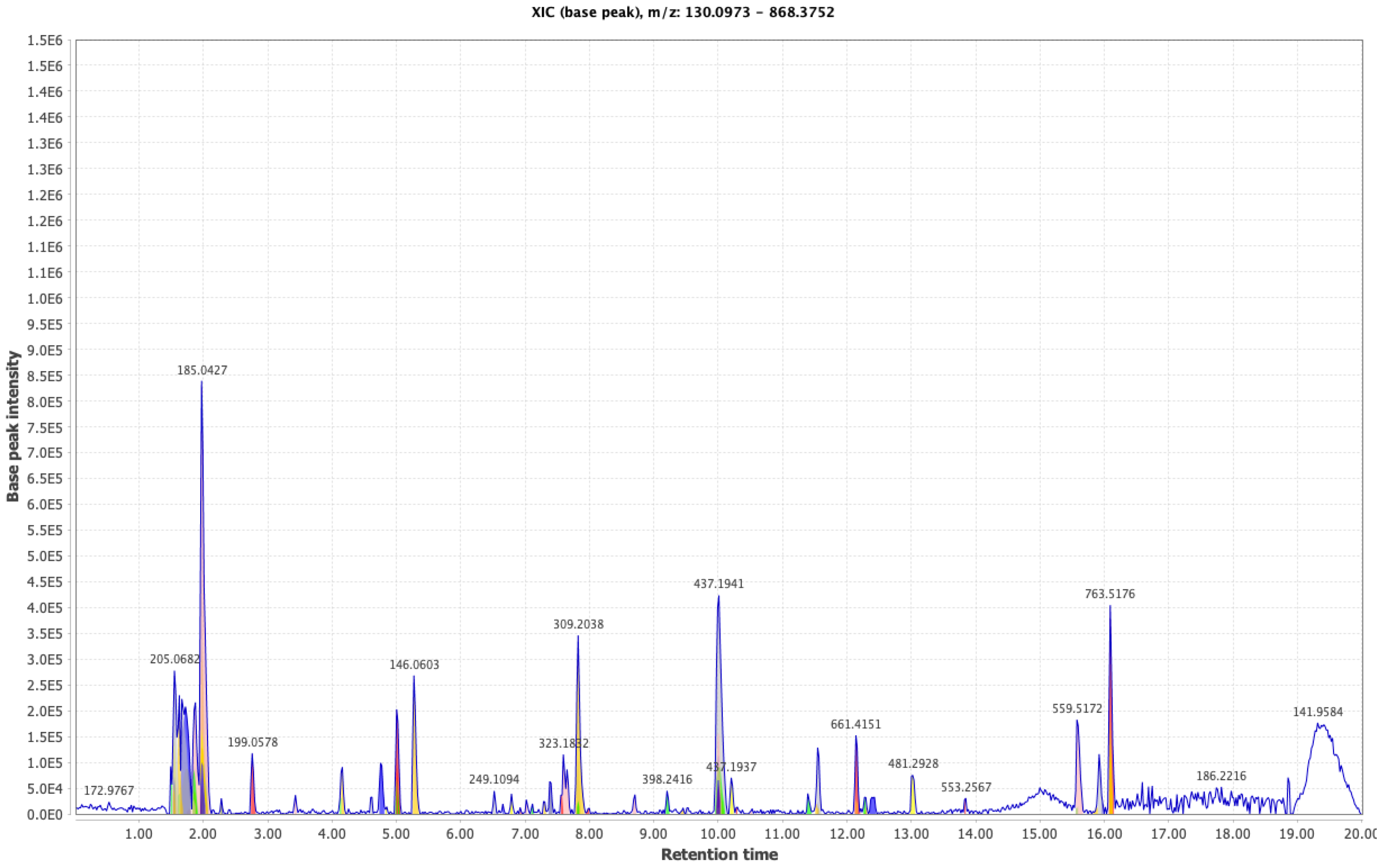


**Figure S12.** Base peak chromatogram (positive ESI) for fungus comb from colony Mn154 extracted in acetonitrile (ACN), 80% ACN fraction (retention time in min).


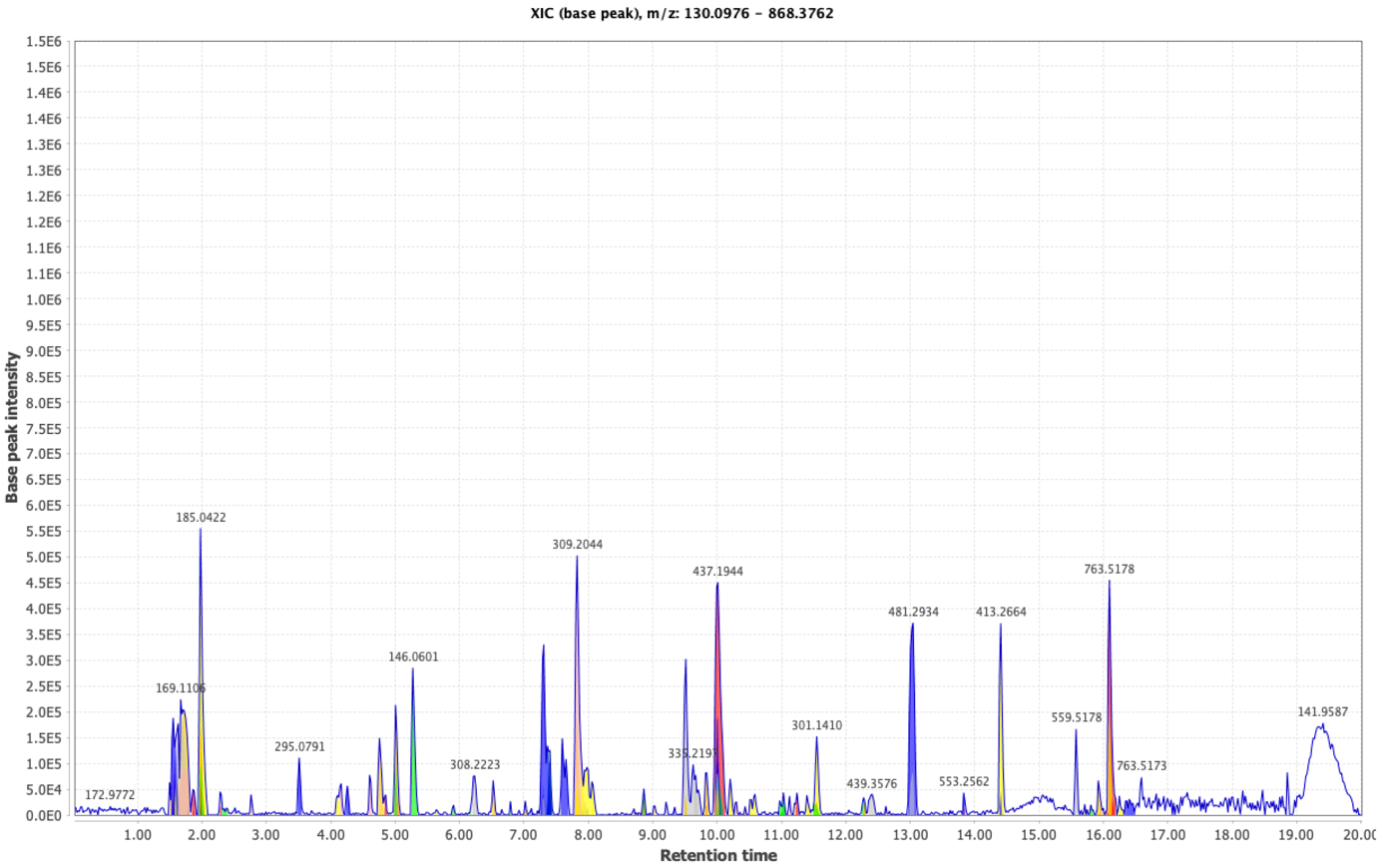


**Figure S13.** Base peak chromatogram (positive ESI) for fungus comb from colony Mn160 extracted in acetonitrile (ACN), 80% ACN fraction (retention time in min).


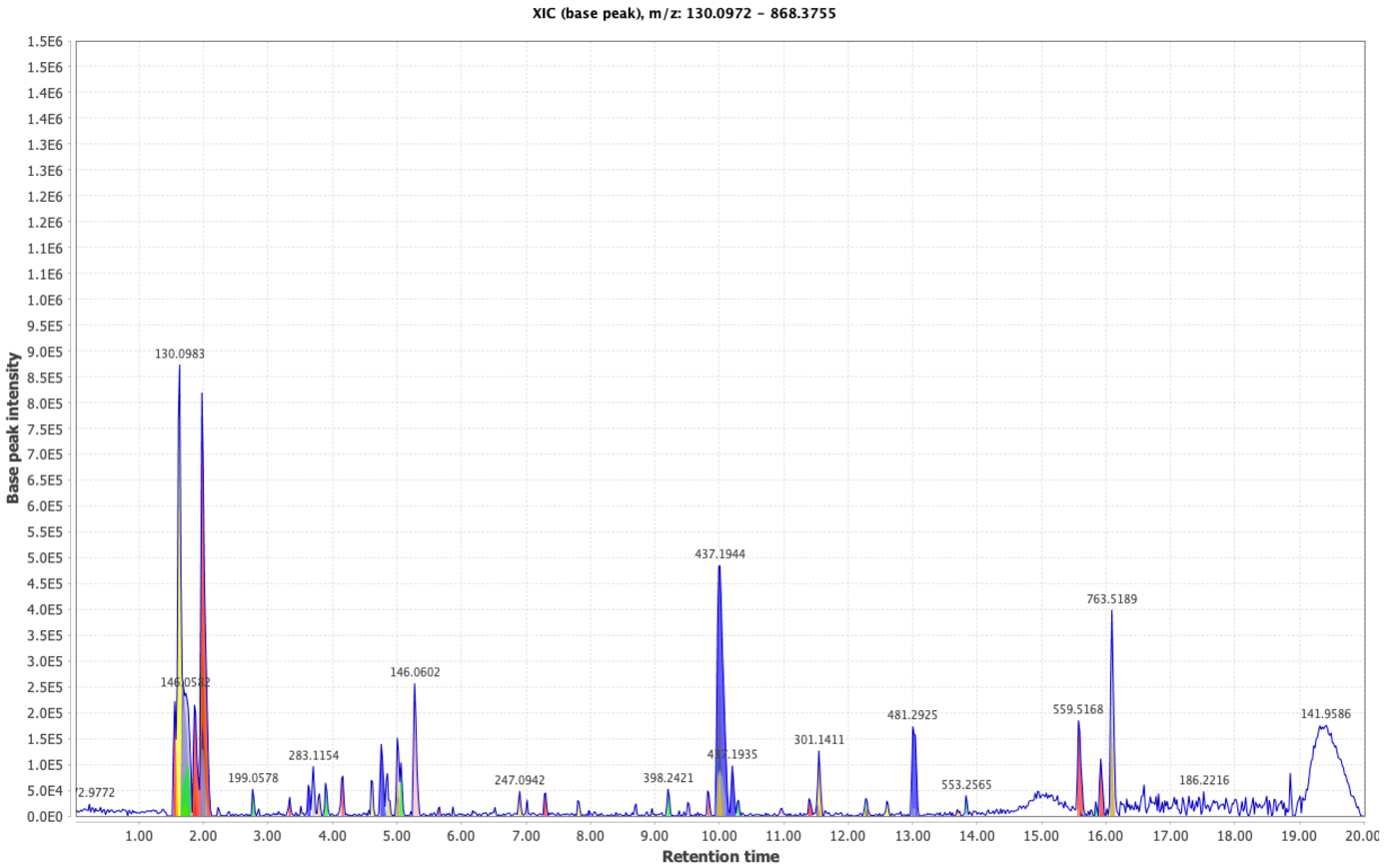


**Figure S14.** Base peak chromatogram (positive ESI) for fungus comb from colony Mn164 extracted in acetonitrile (ACN), 80% ACN fraction (retention time in min).


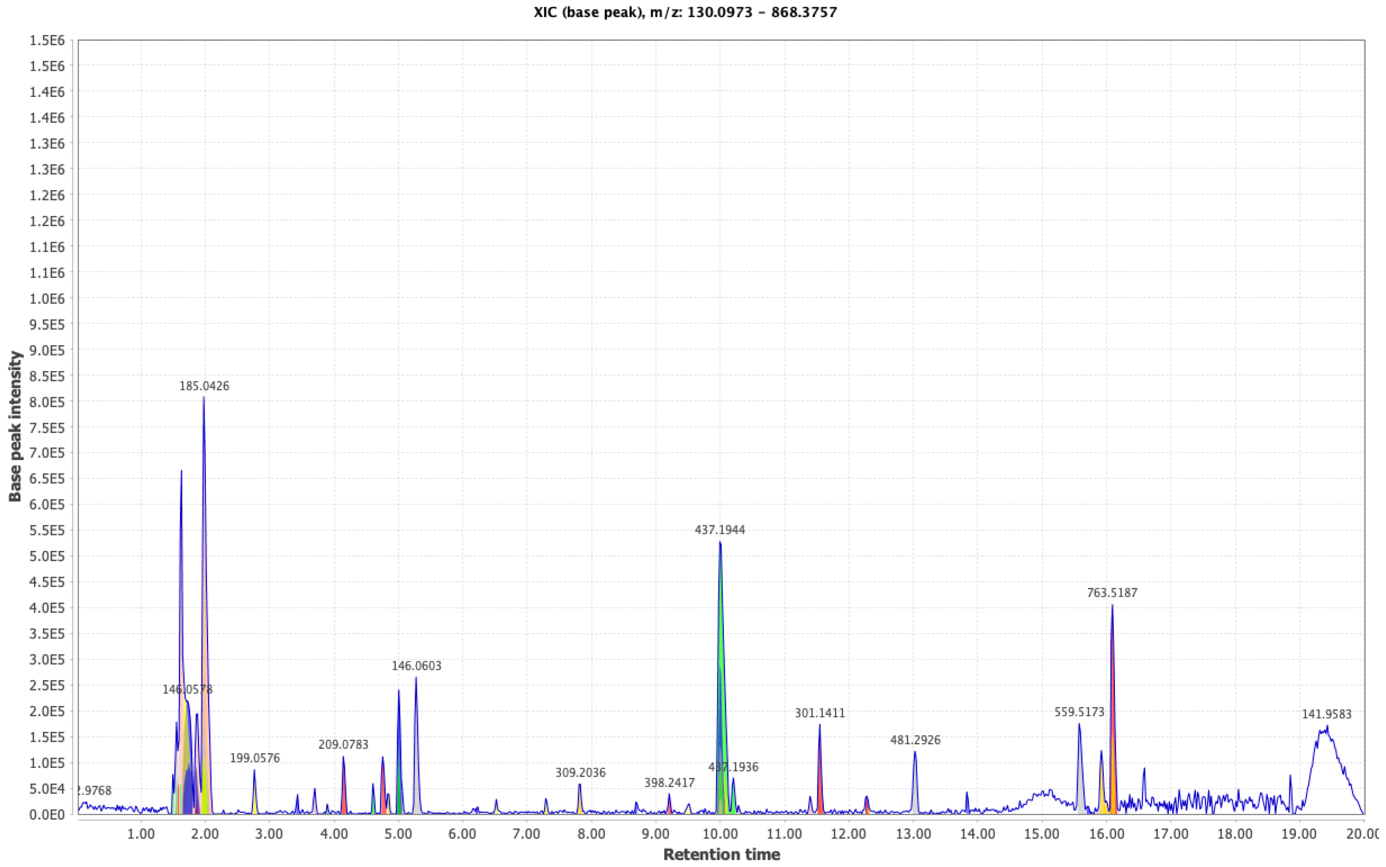


**Figure S15.** Base peak chromatogram (positive ESI) for fungus comb from colony Mn165 extracted in acetonitrile (ACN), 80% ACN fraction (retention time in min).


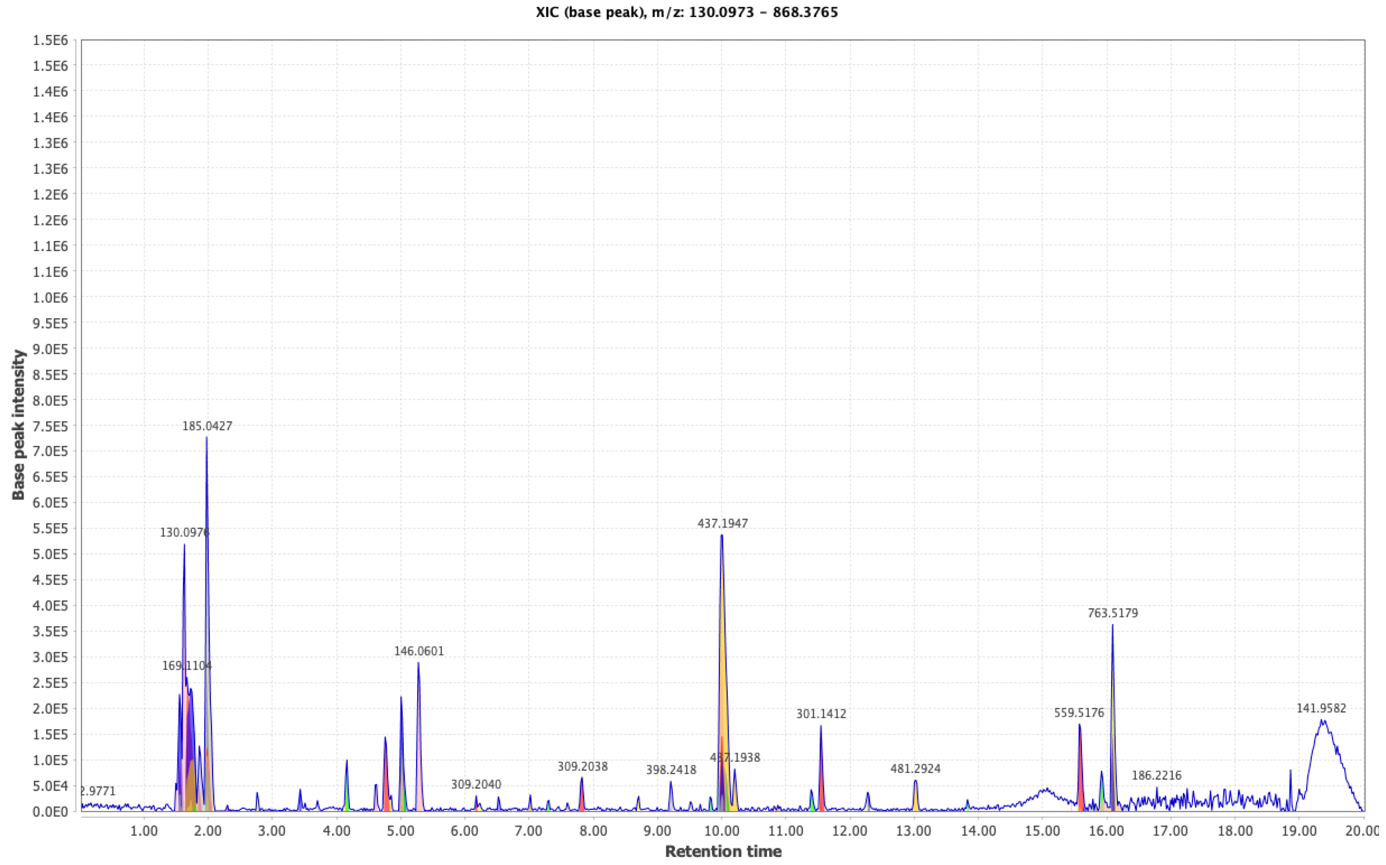


**Figure S16.** Base peak chromatogram (positive ESI) for fungus comb from colony Mn166 extracted in acetonitrile (ACN), 80% ACN fraction (retention time in min).


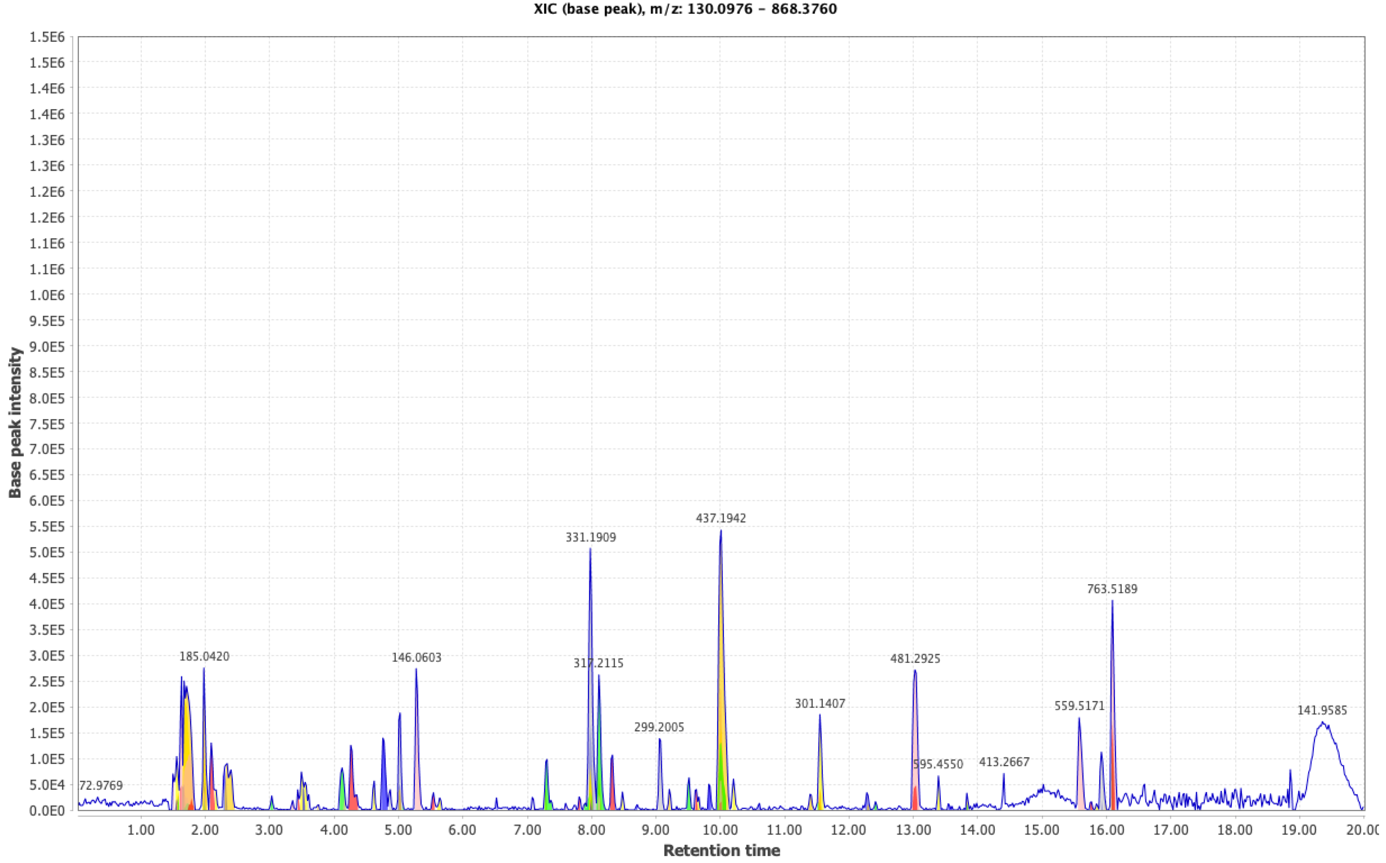


**Figure S17.** Base peak chromatogram (positive ESI) for fungus comb from colony Mn171 extracted in acetonitrile (ACN), 80% ACN fraction (retention time in min).


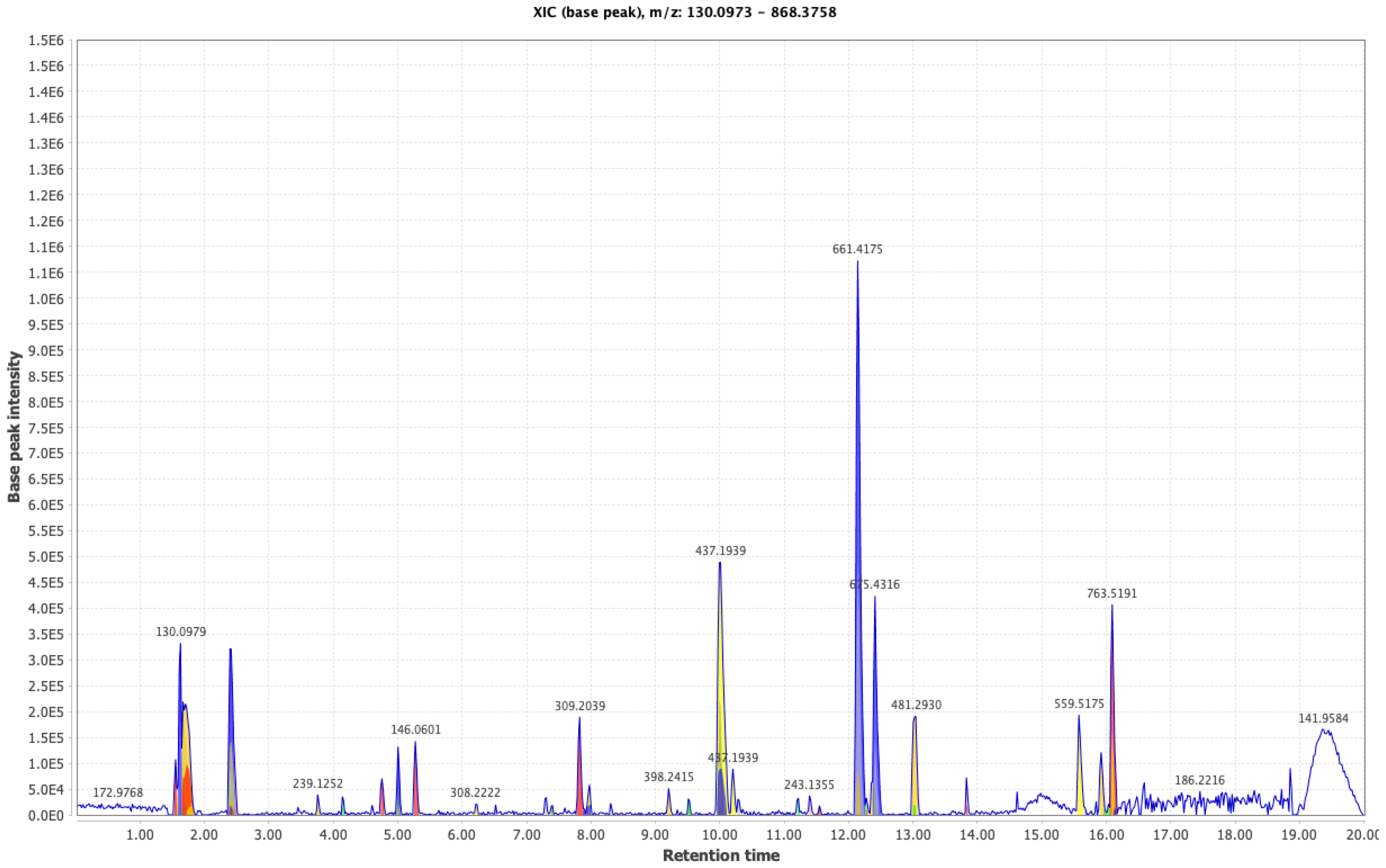


**Figure S18.** Base peak chromatogram (positive ESI) for fungus comb from colony Od127 extracted in acetonitrile (ACN), 80% ACN fraction (retention time in min).

**
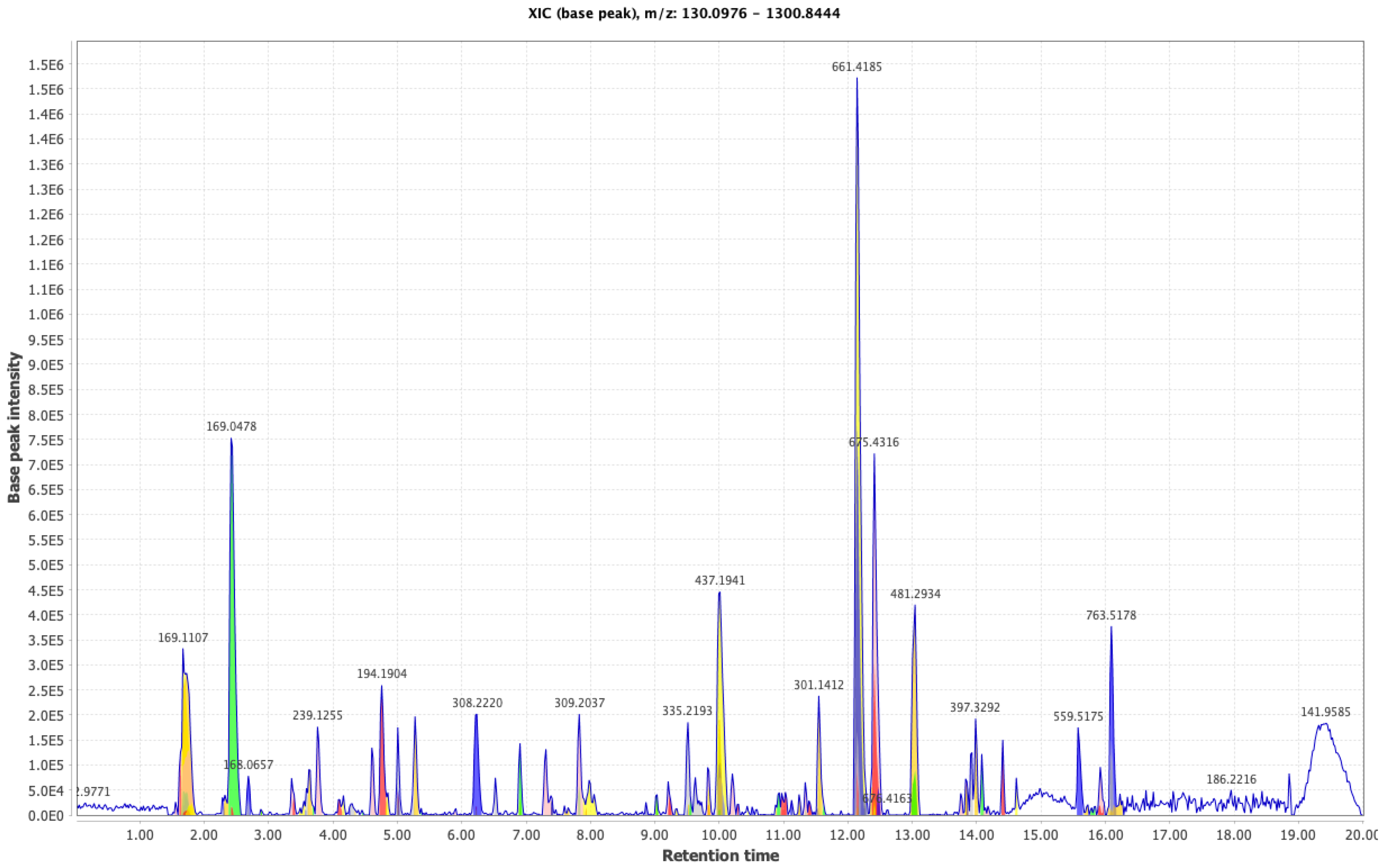
**

**Figure S19.** Base peak chromatogram (positive ESI) for fungus comb from colony Od152 extracted in acetonitrile (ACN), 80% ACN fraction (retention time in min).


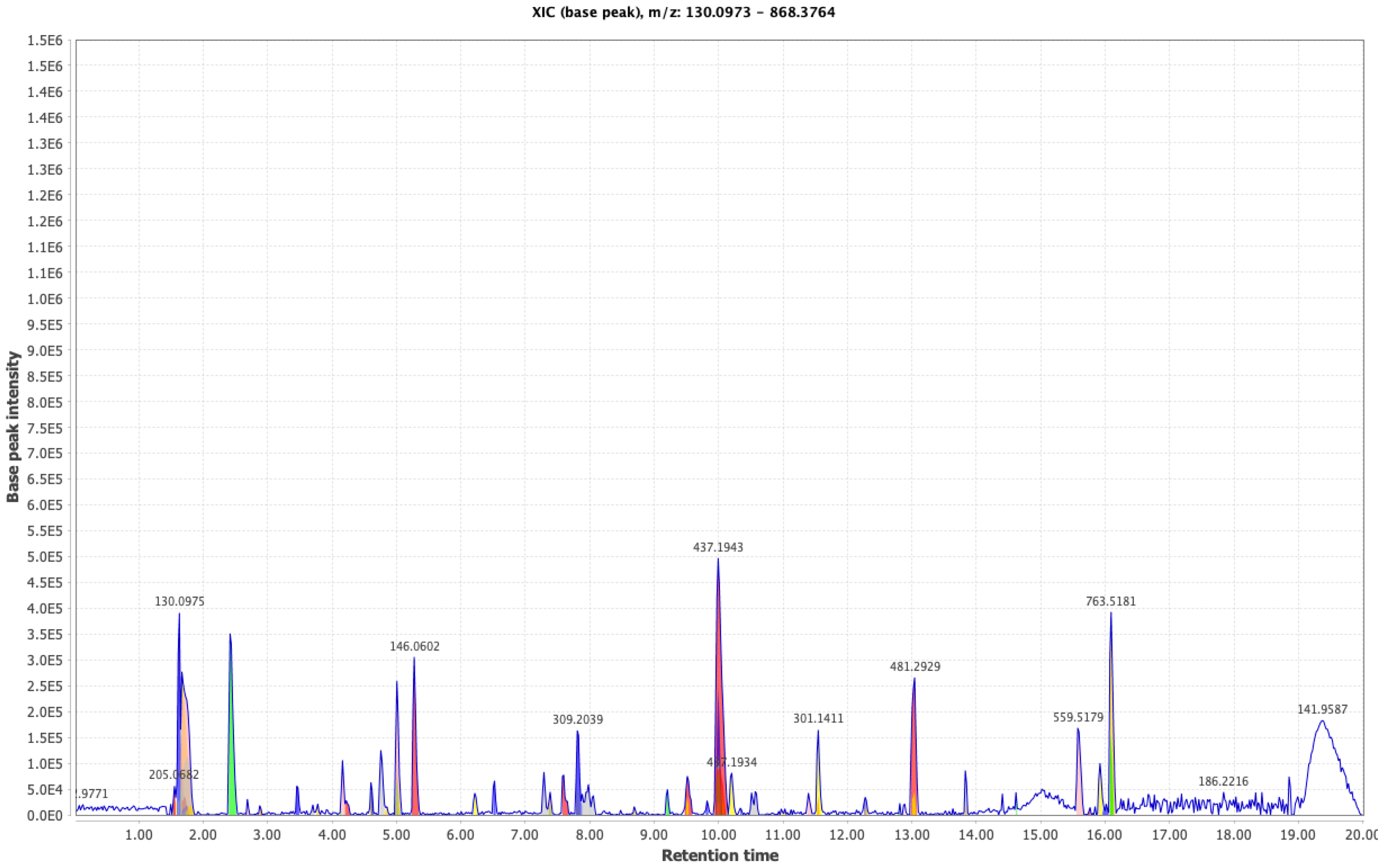


**Figure S20.** Base peak chromatogram (positive ESI) for fungus comb from colony Od167 extracted in acetonitrile (ACN), 80% ACN fraction (retention time in min).


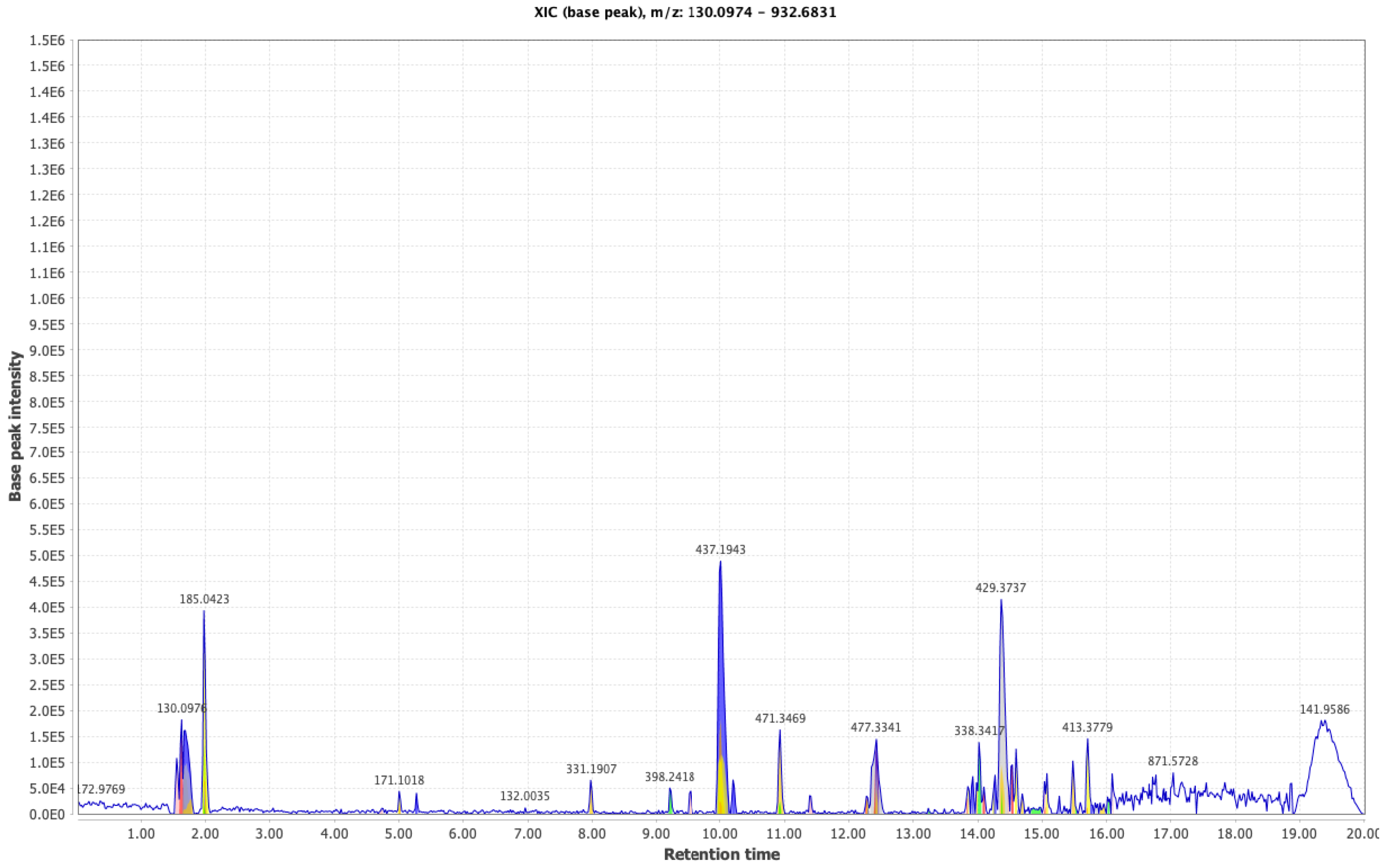


**Figure S21.** Base peak chromatogram (positive ESI) for fungus comb from colony Mn3.2 extracted in acetonitrile (ACN), 100% ACN fraction (retention time in min).


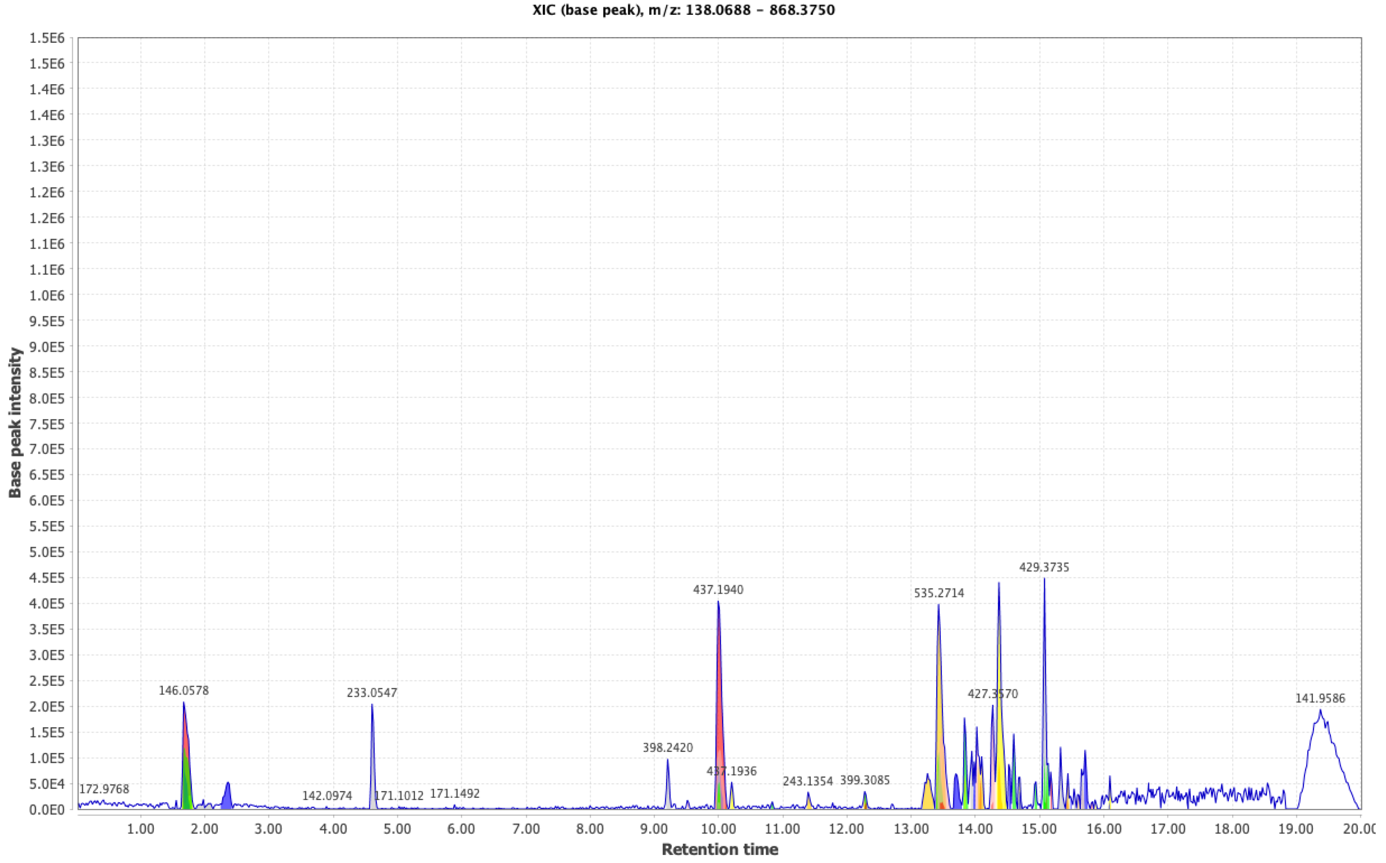


**Figure S22.** Base peak chromatogram (positive ESI) for fungus comb from colony Mn132 extracted in acetonitrile (ACN), 100% ACN fraction (retention time in min).


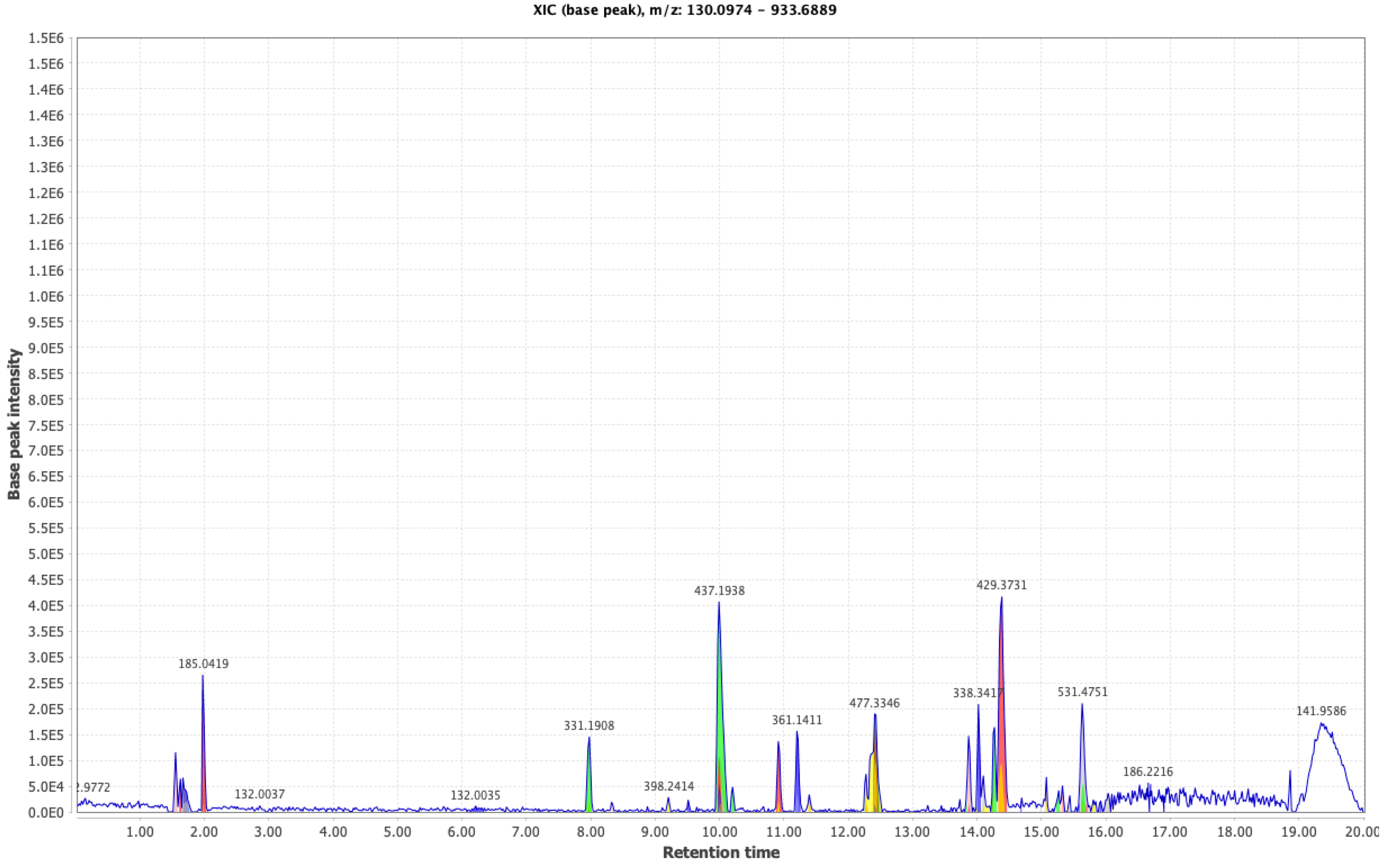


**Figure S23.** Base peak chromatogram (positive ESI) for fungus comb from colony Mn153 extracted in acetonitrile (ACN), 100% ACN fraction (retention time in min).


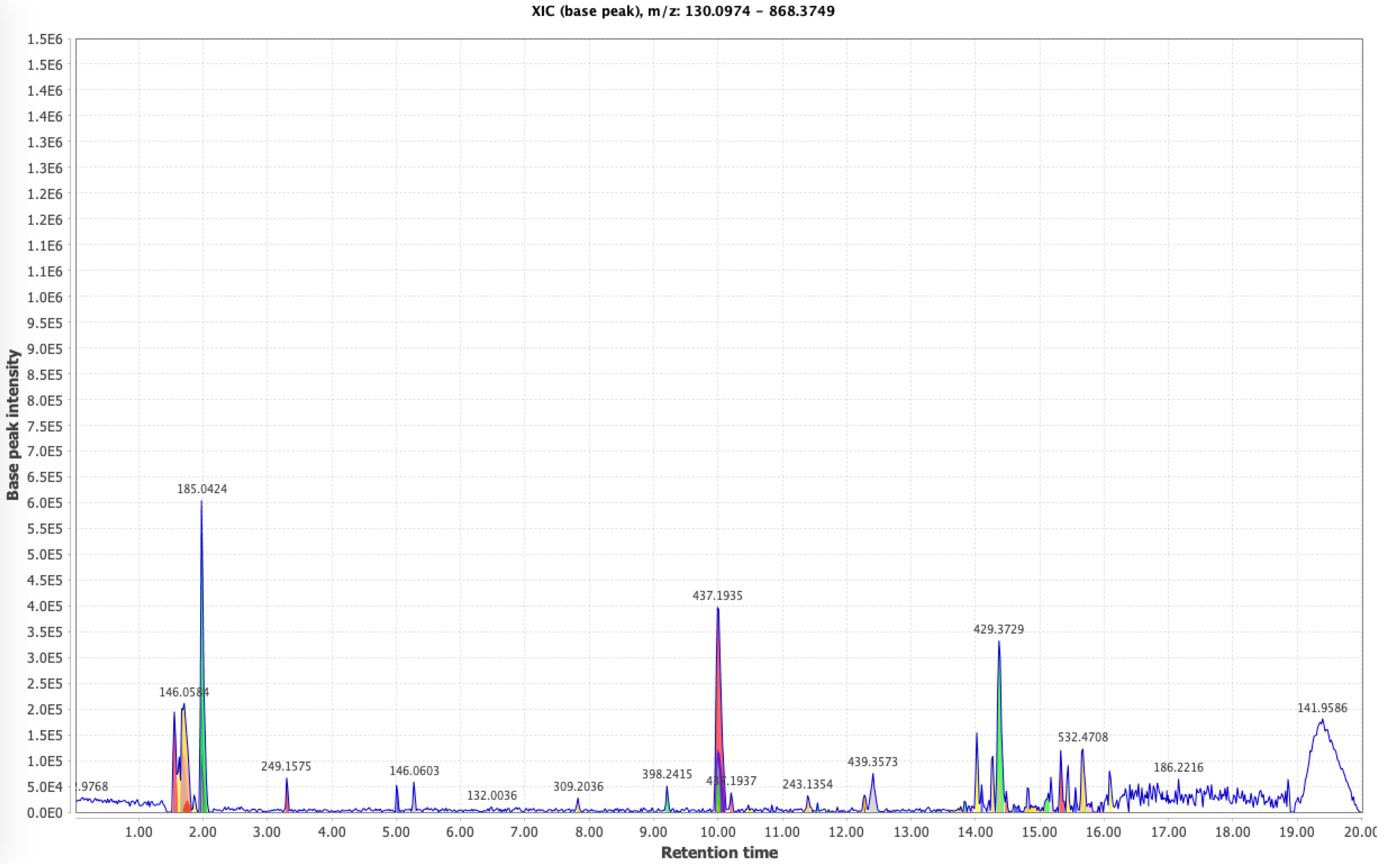


**Figure S24.** Base peak chromatogram (positive ESI) for fungus comb from colony Mn154 extracted in acetonitrile (ACN), 100% ACN fraction (retention time in min).


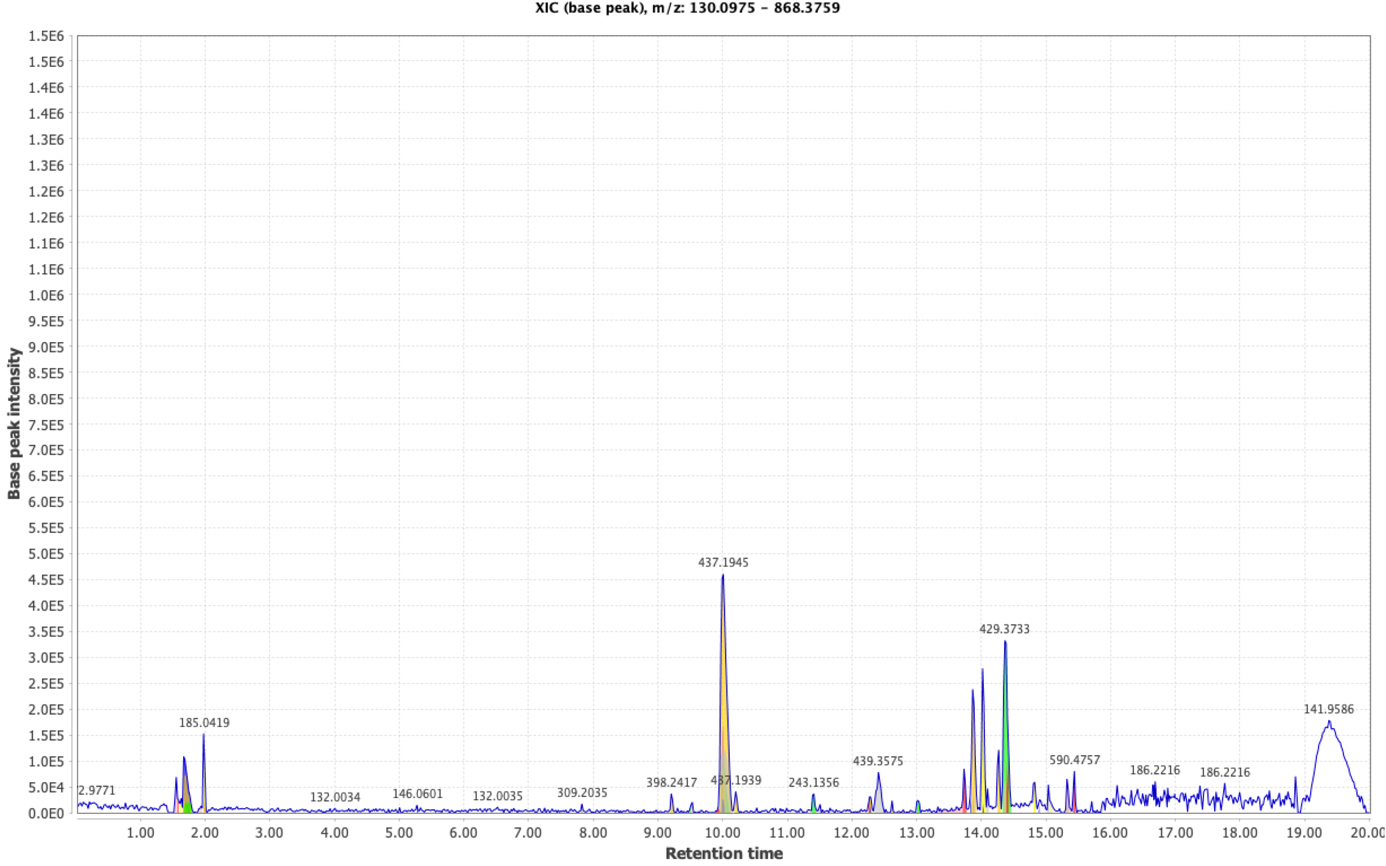


**Figure S25.** Base peak chromatogram (positive ESI) for fungus comb from colony Mn160 extracted in acetonitrile (ACN), 100% ACN fraction (retention time in min).


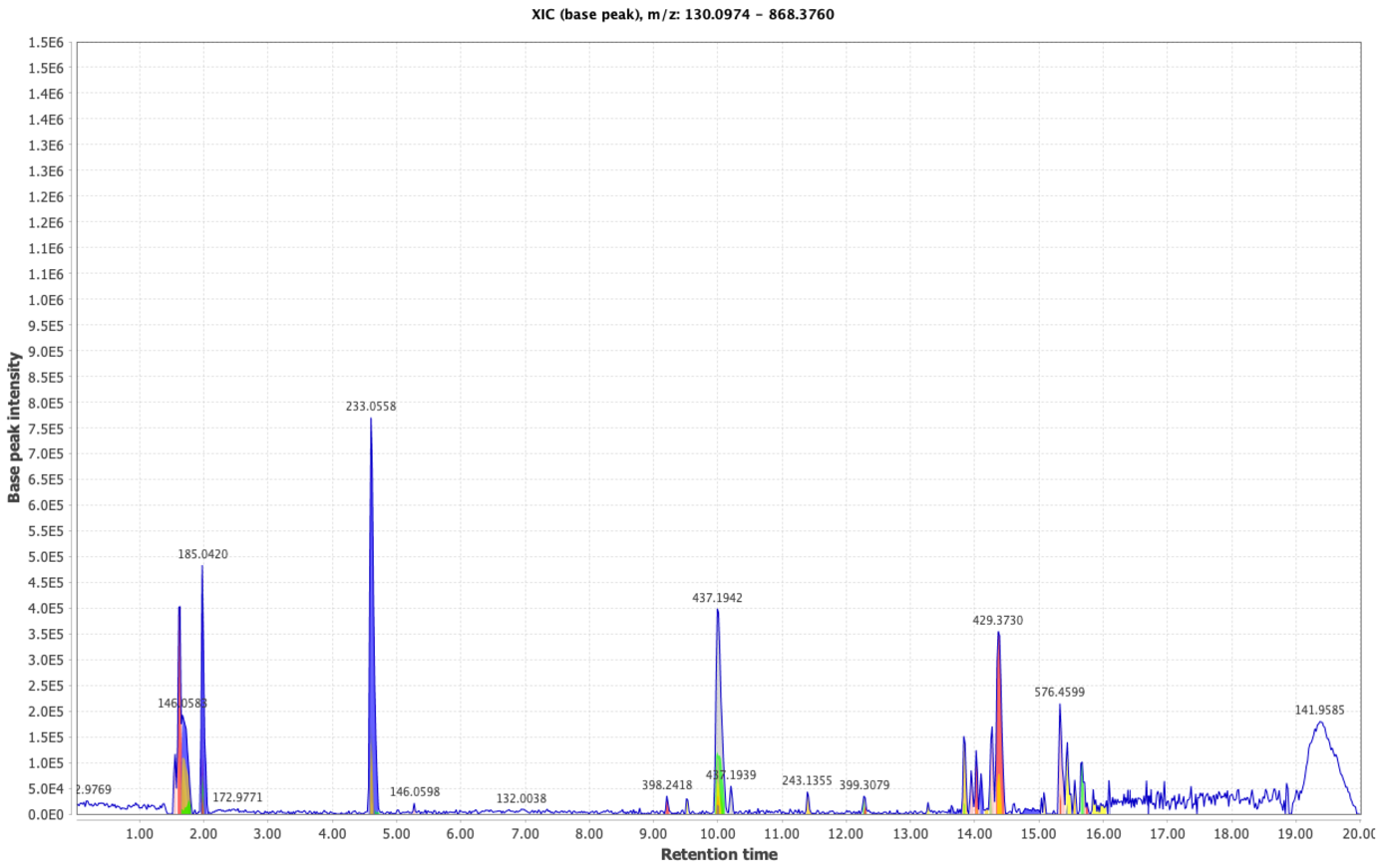


**Figure S26.** Base peak chromatogram (positive ESI) for fungus comb from colony Mn164 extracted in acetonitrile (ACN), 100% ACN fraction (retention time in min).


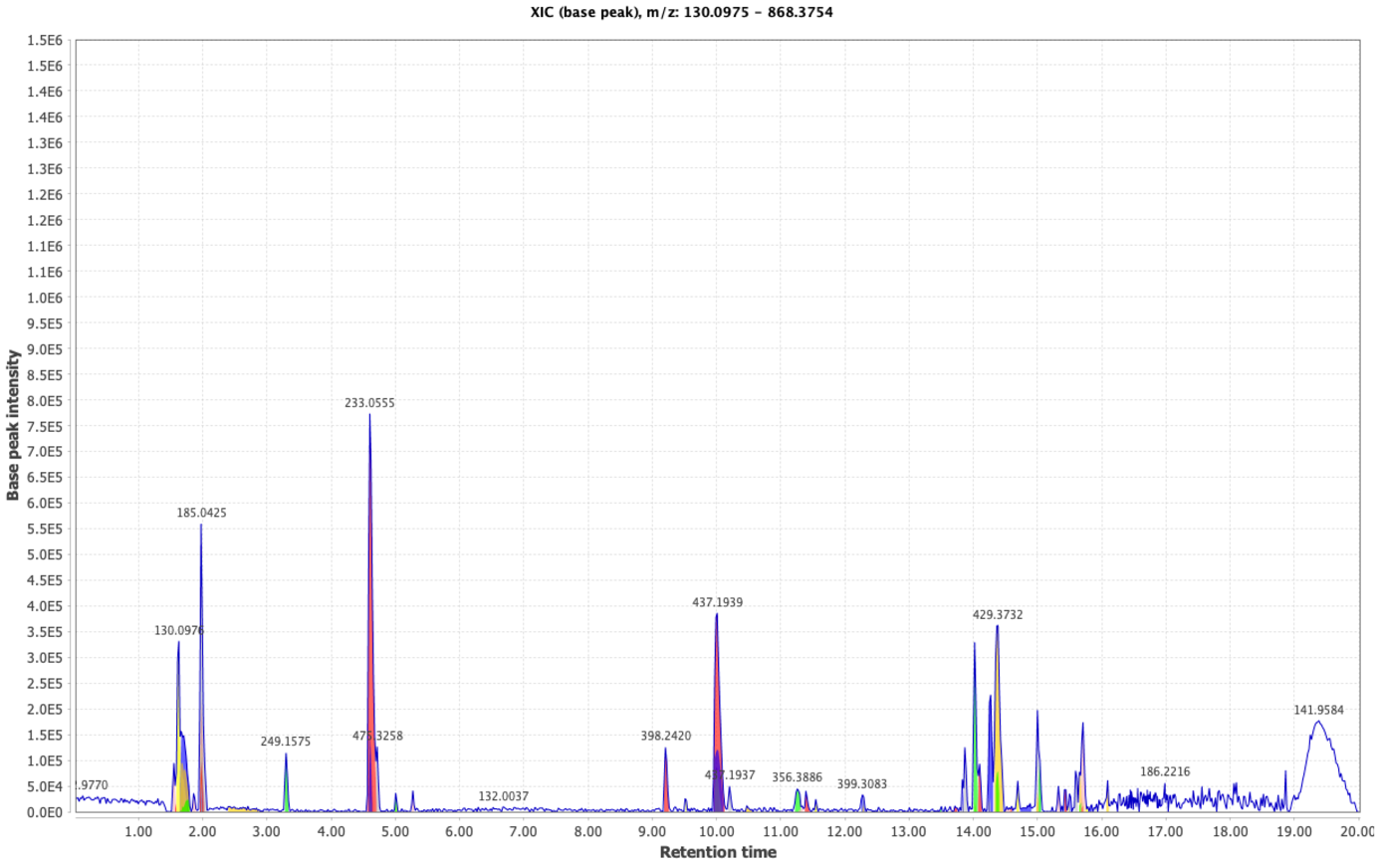


**Figure S27.** Base peak chromatogram (positive ESI) for fungus comb from colony Mn165 extracted in acetonitrile (ACN), 100% ACN fraction (retention time in min).


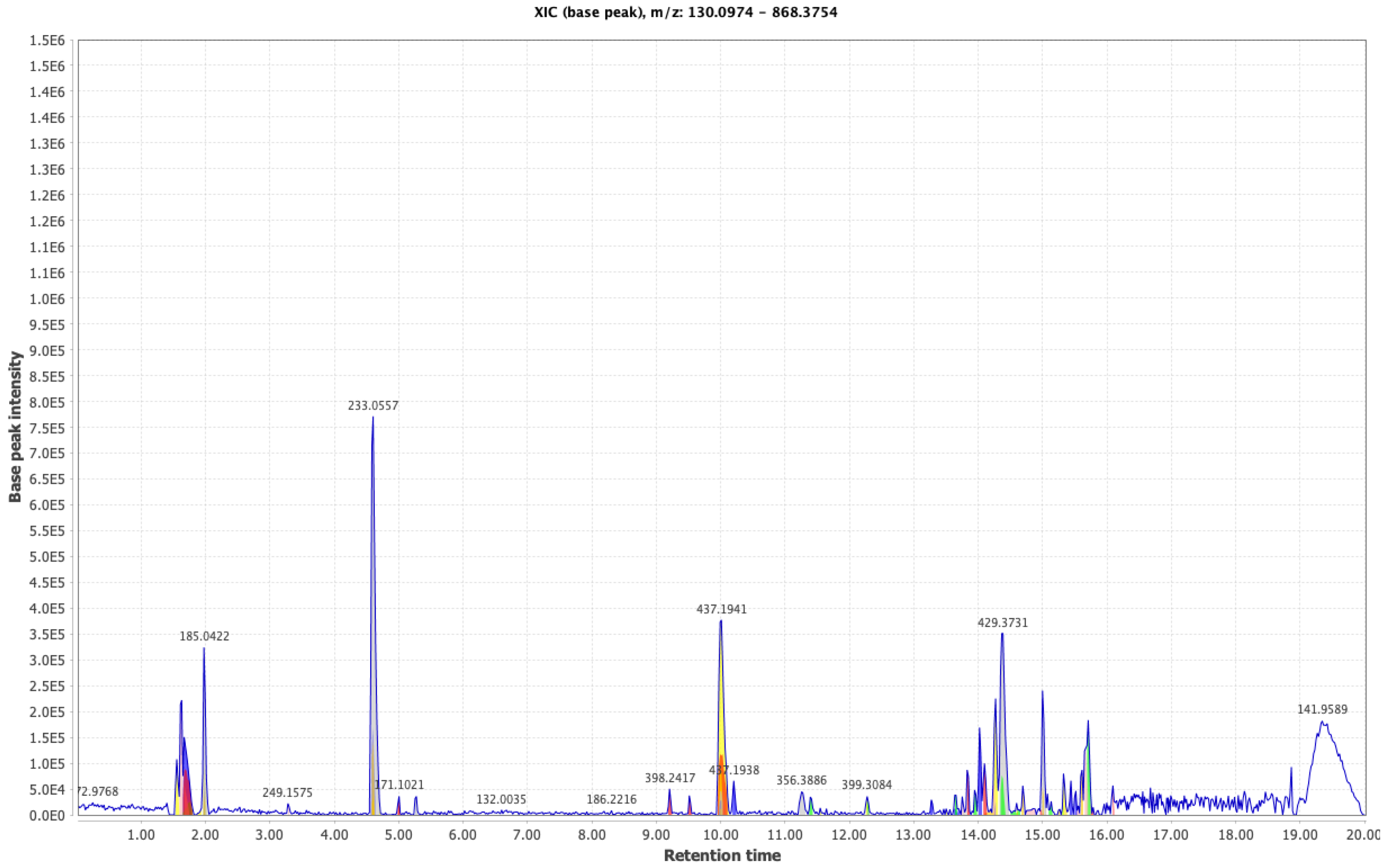


**Figure S28.** Base peak chromatogram (positive ESI) for fungus comb from colony Mn166 extracted in acetonitrile (ACN), 100% ACN fraction (retention time in min).


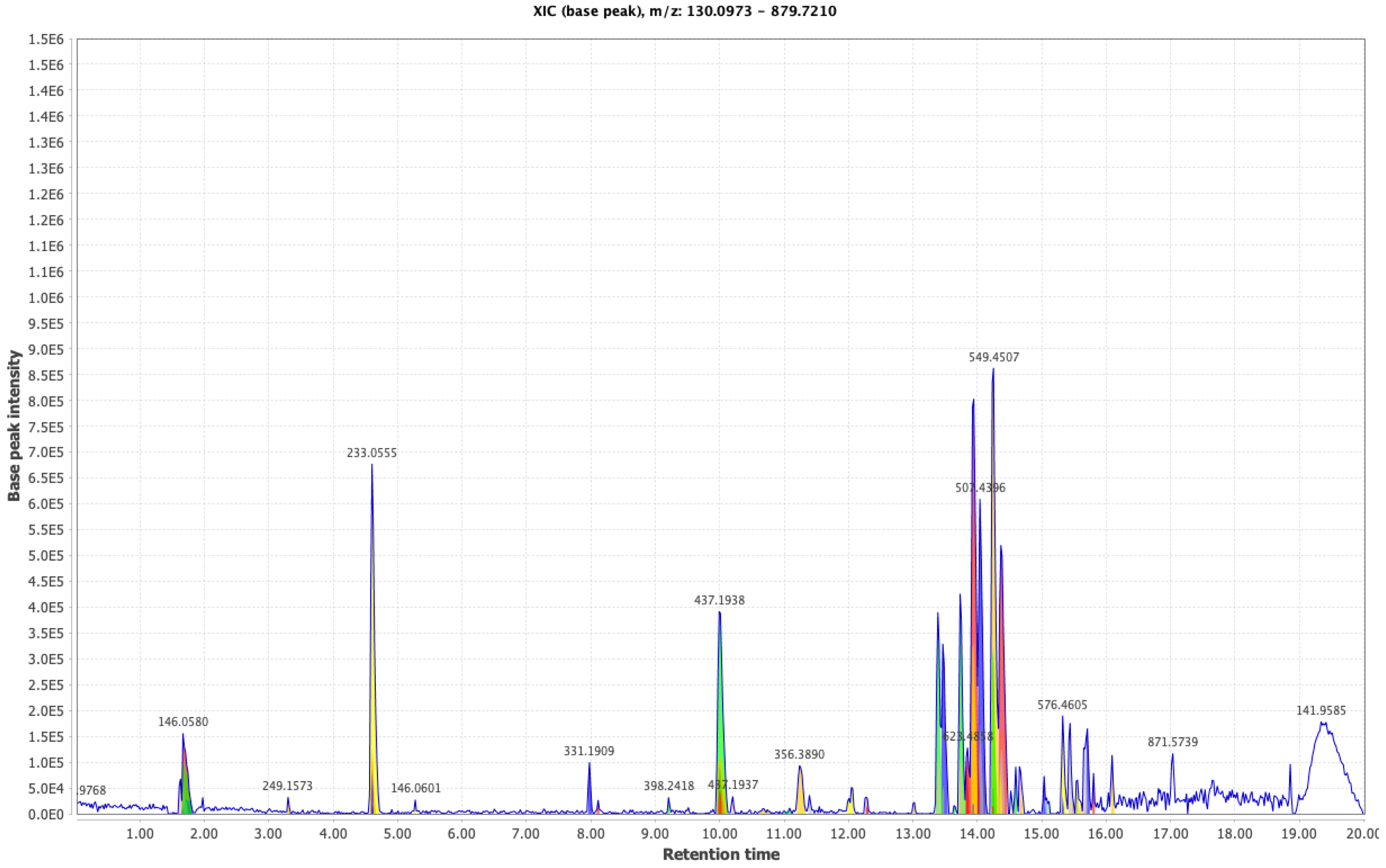


**Figure S29.** Base peak chromatogram (positive ESI) for fungus comb from colony Mn171 extracted in acetonitrile (ACN), 100% ACN fraction (retention time in min).


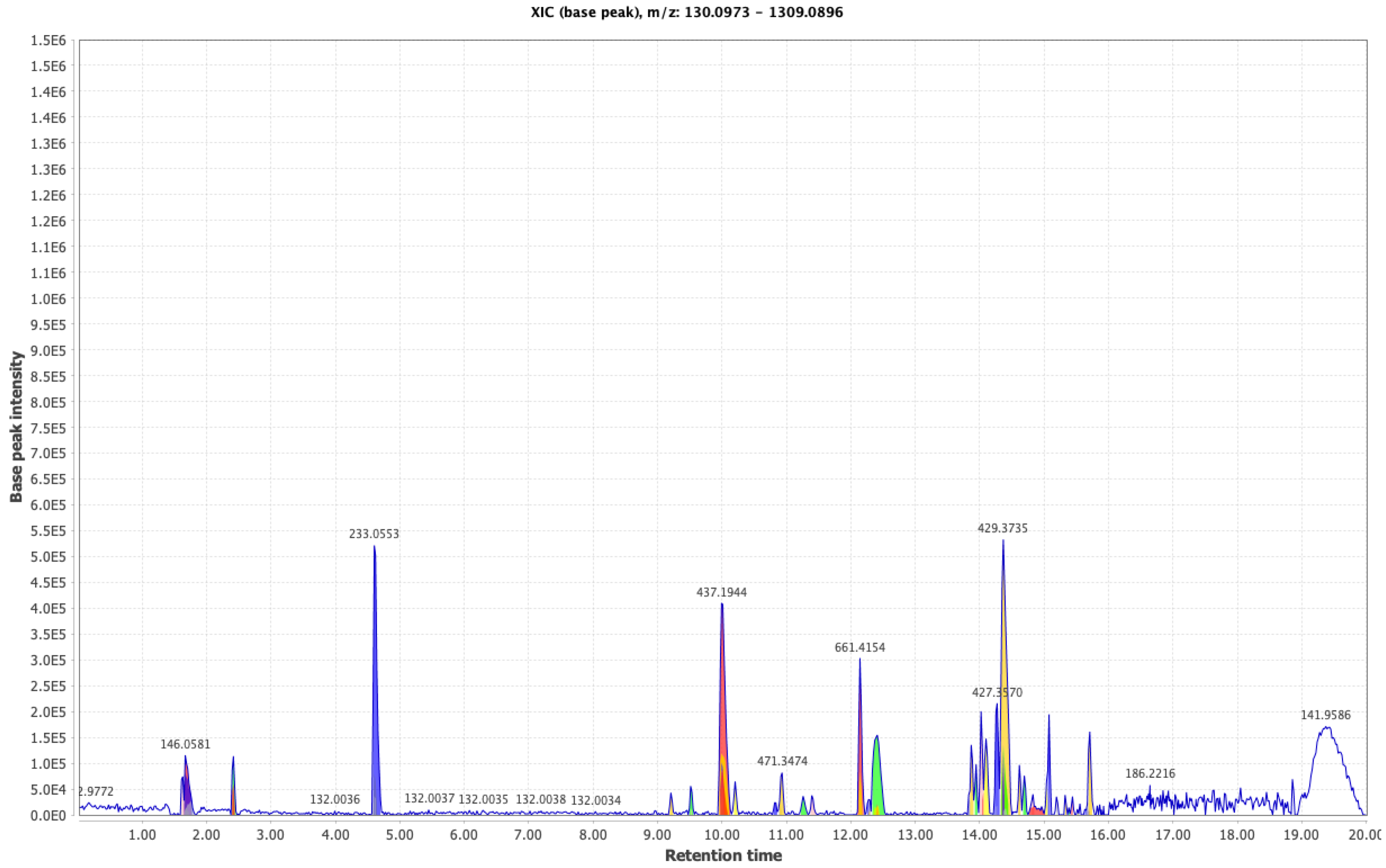


**Figure S30.** Base peak chromatogram (positive ESI) for fungus comb from colony Od127 extracted in acetonitrile (ACN), 100% ACN fraction (retention time in min).


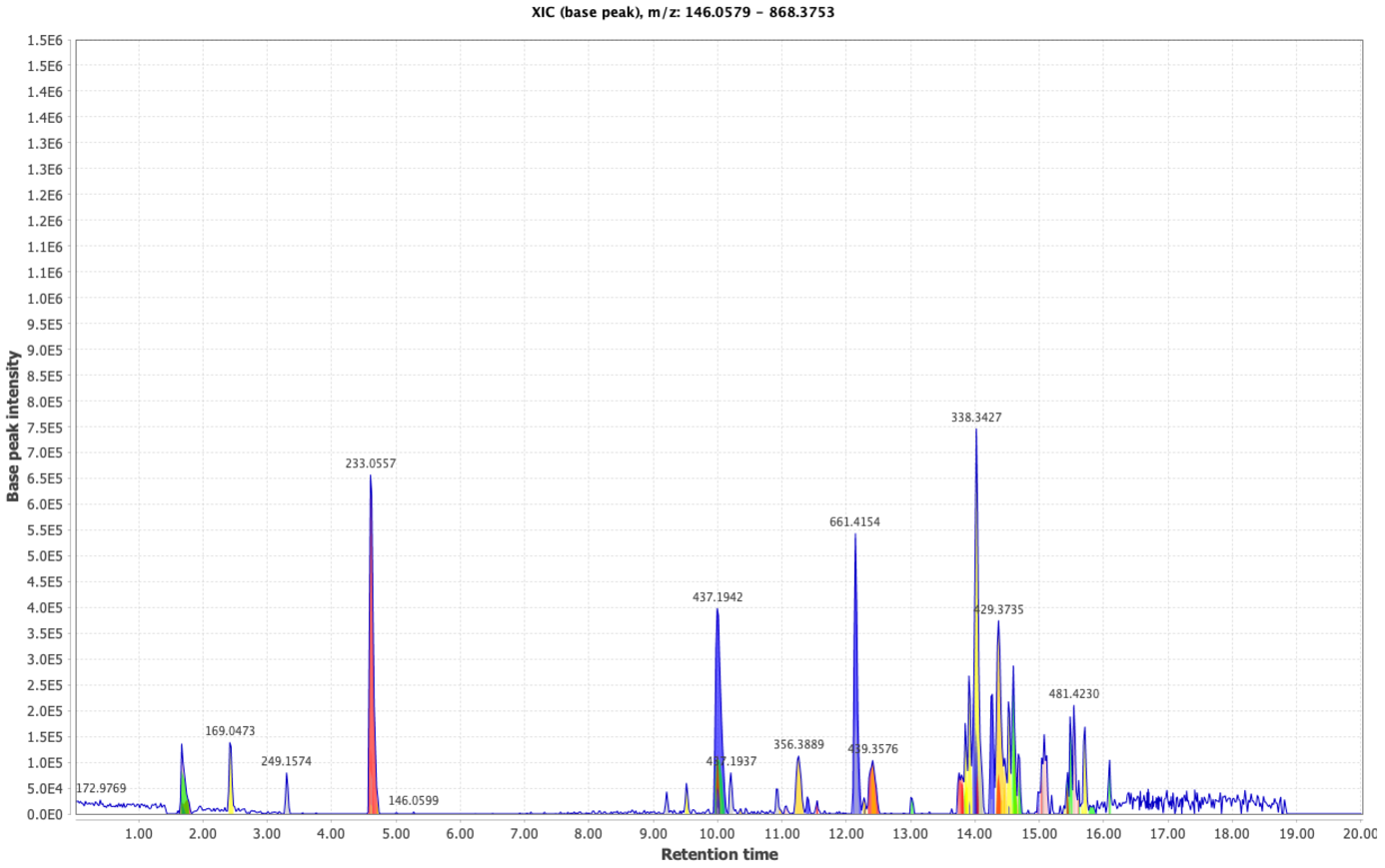


**Figure S31.** Base peak chromatogram (positive ESI) for fungus comb from colony Od152 extracted in acetonitrile (ACN), 100% ACN fraction (retention time in min).


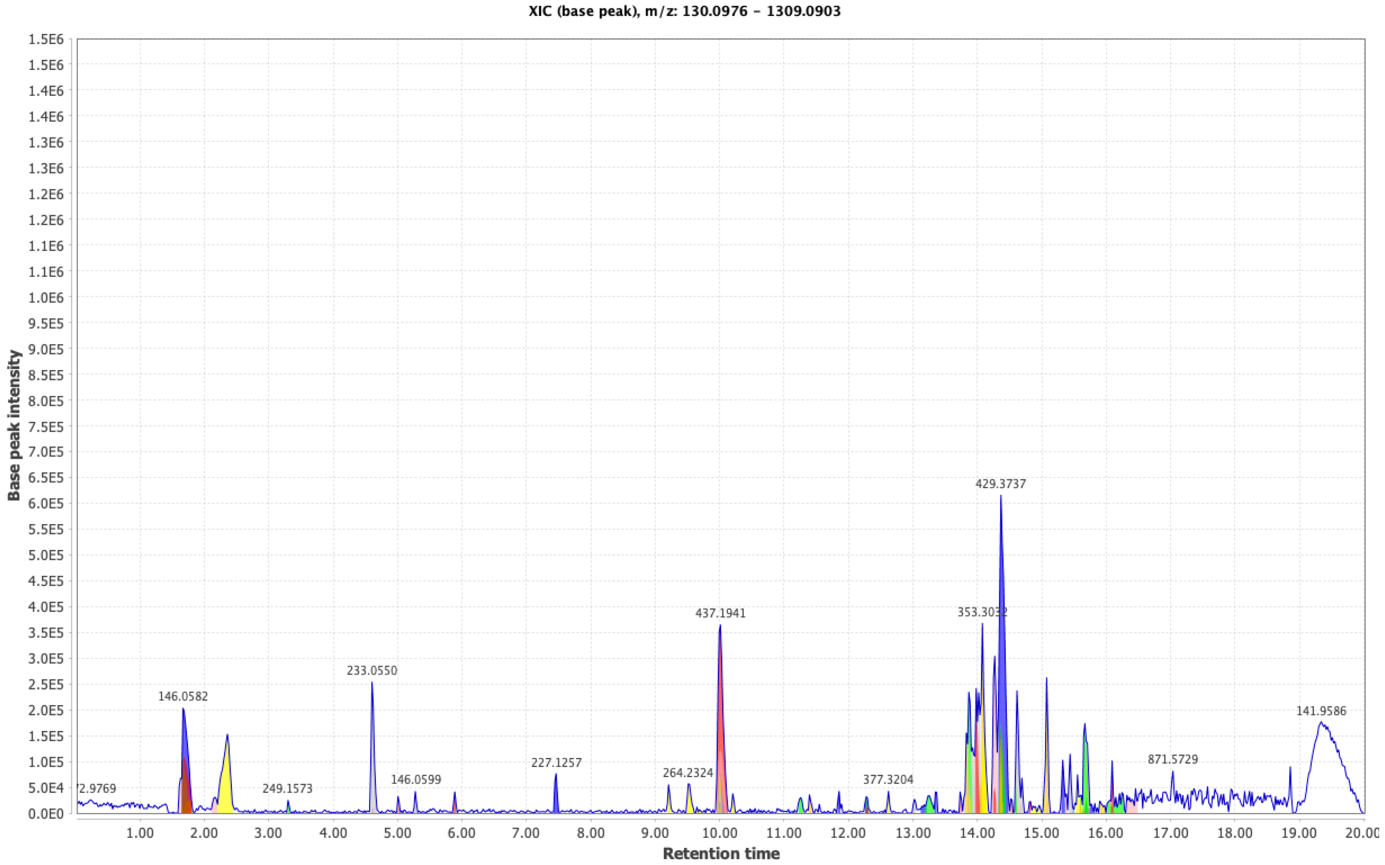


**Figure S32.** Base peak chromatogram (positive ESI) for fungus comb from colony Od167 extracted in acetonitrile (ACN), 100% ACN fraction (retention time in min).


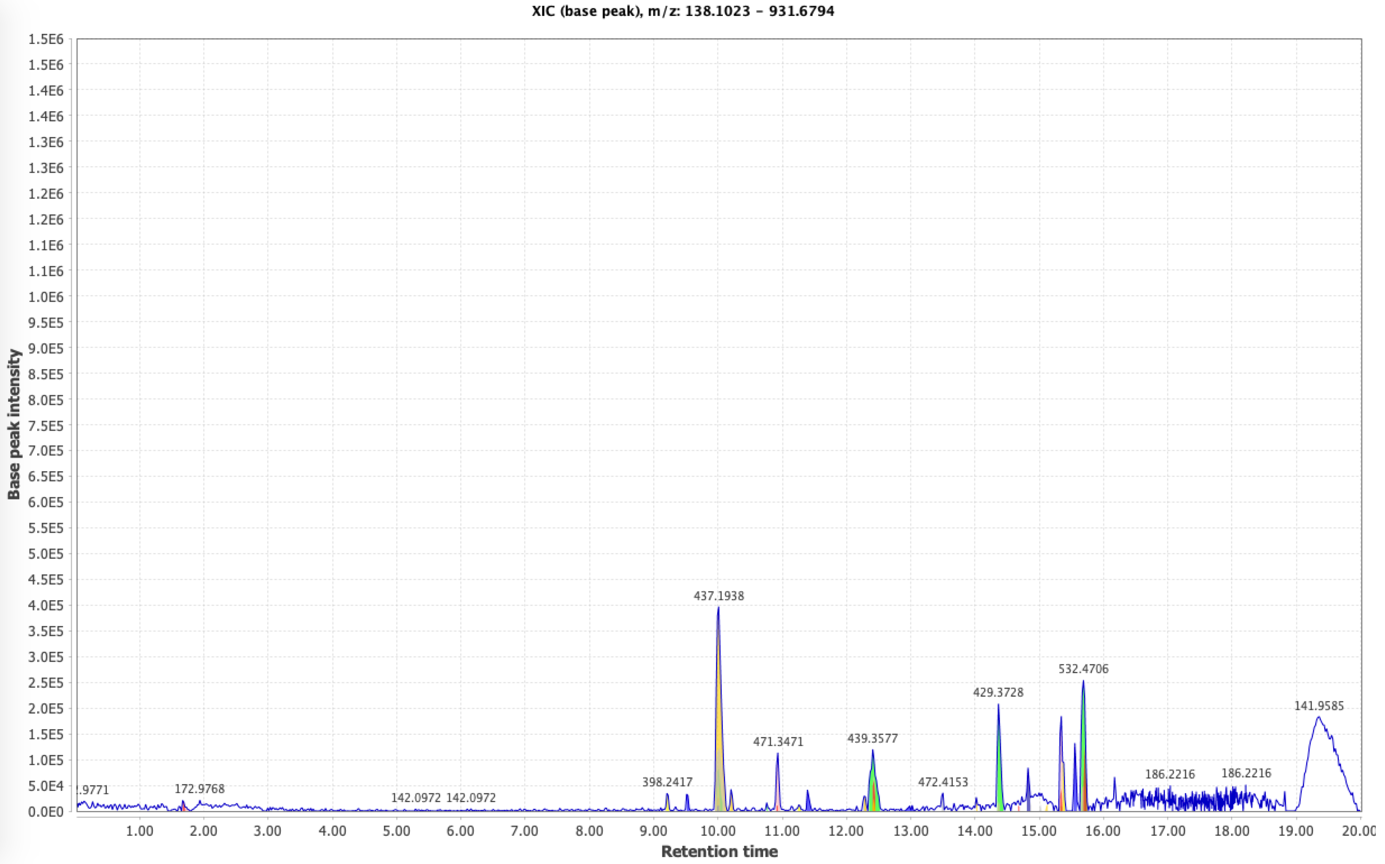


**Figure S33.** Base peak chromatogram (positive ESI) for fungus comb from colony Mn3.2 extracted in acetonitrile (ACN), 100% acetone fraction (retention time in min).


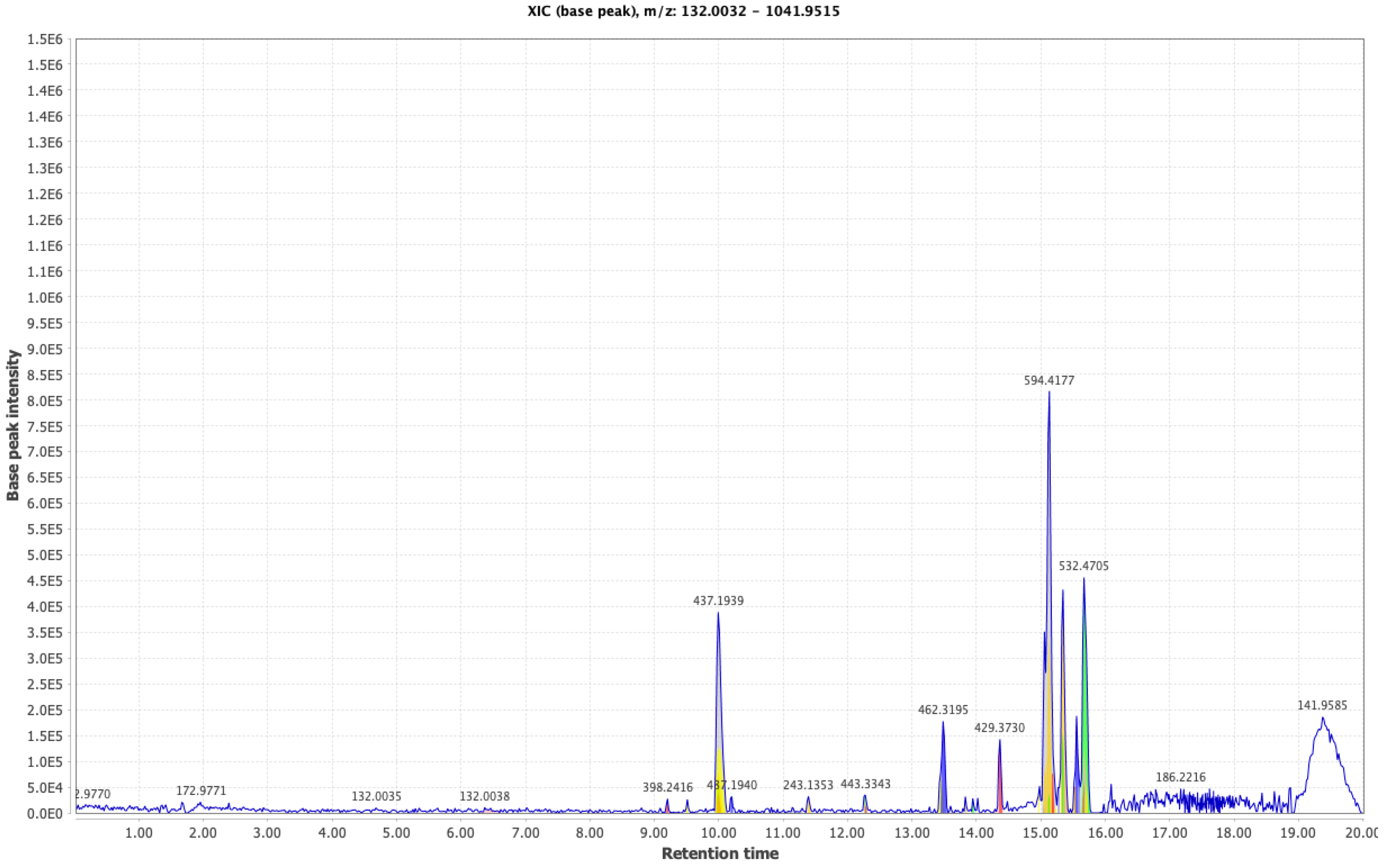


**Figure S34.** Base peak chromatogram (positive ESI) for fungus comb from colony Mn132 extracted in acetonitrile (ACN), 100% acetone fraction (retention time in min).


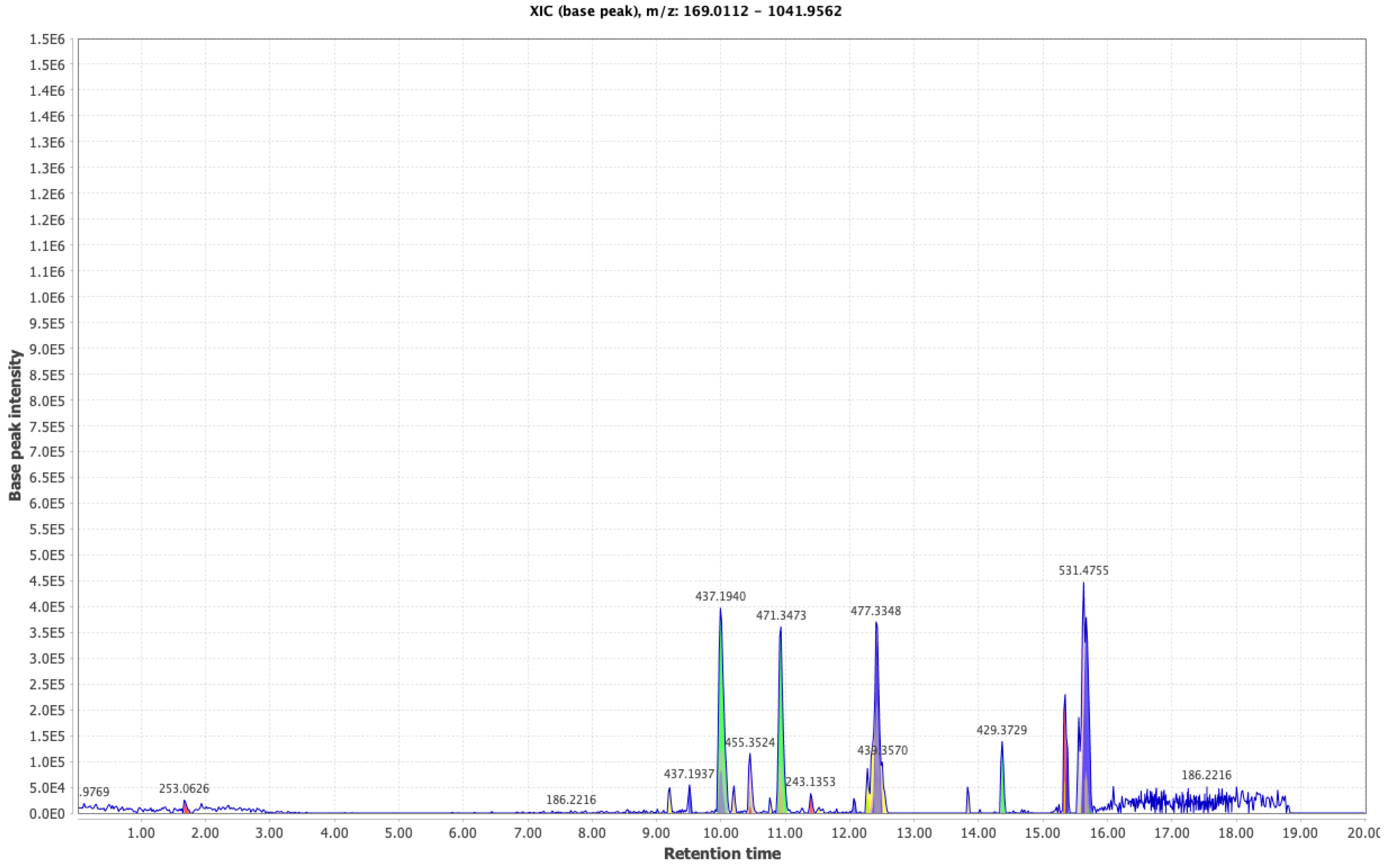


**Figure S35.** Base peak chromatogram (positive ESI) for fungus comb from colony Mn153 extracted in acetonitrile (ACN), 100% acetone fraction (retention time in min).


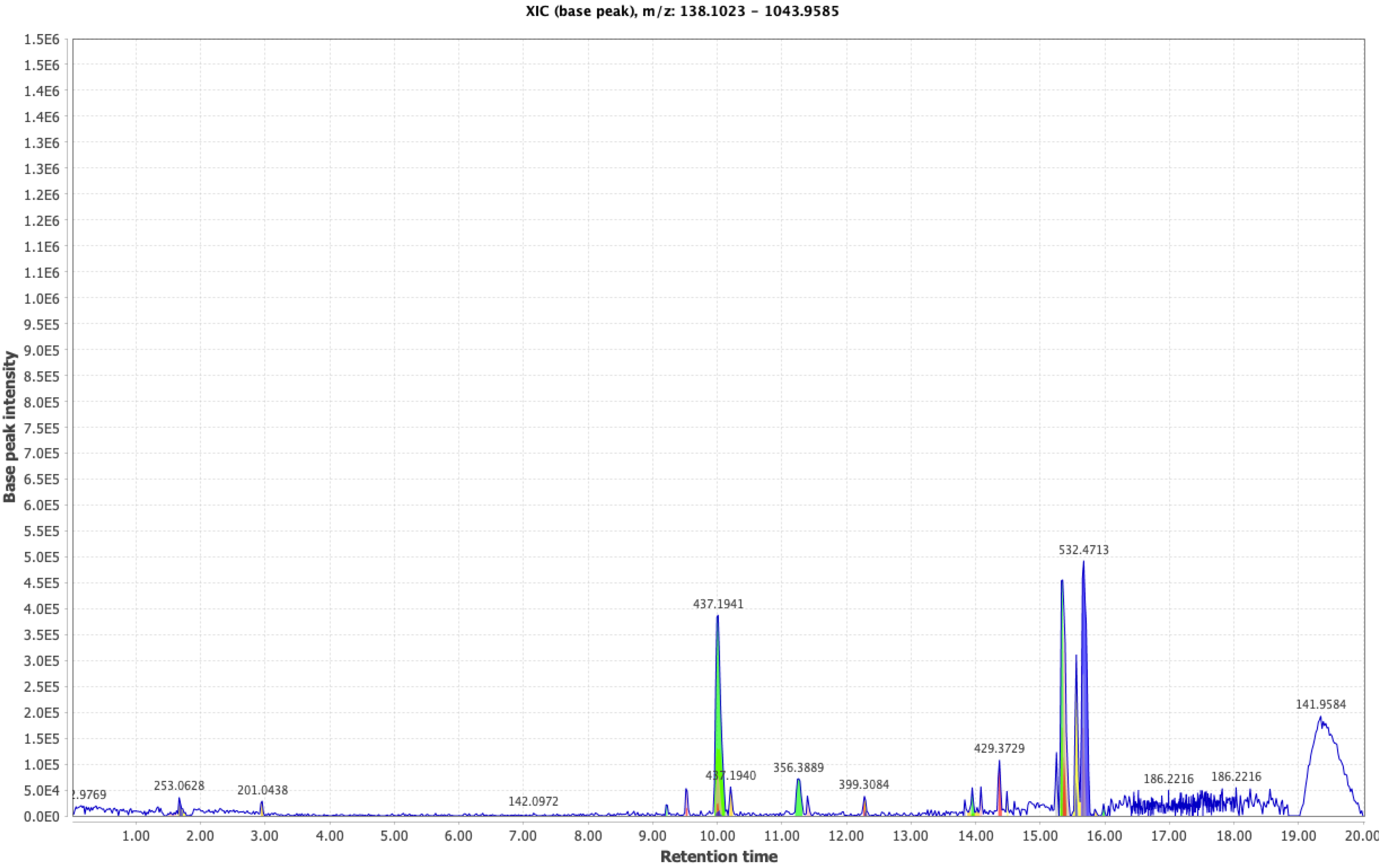


**Figure S36.** Base peak chromatogram (positive ESI) for fungus comb from colony Mn154 extracted in acetonitrile (ACN), 100% acetone fraction (retention time in min).


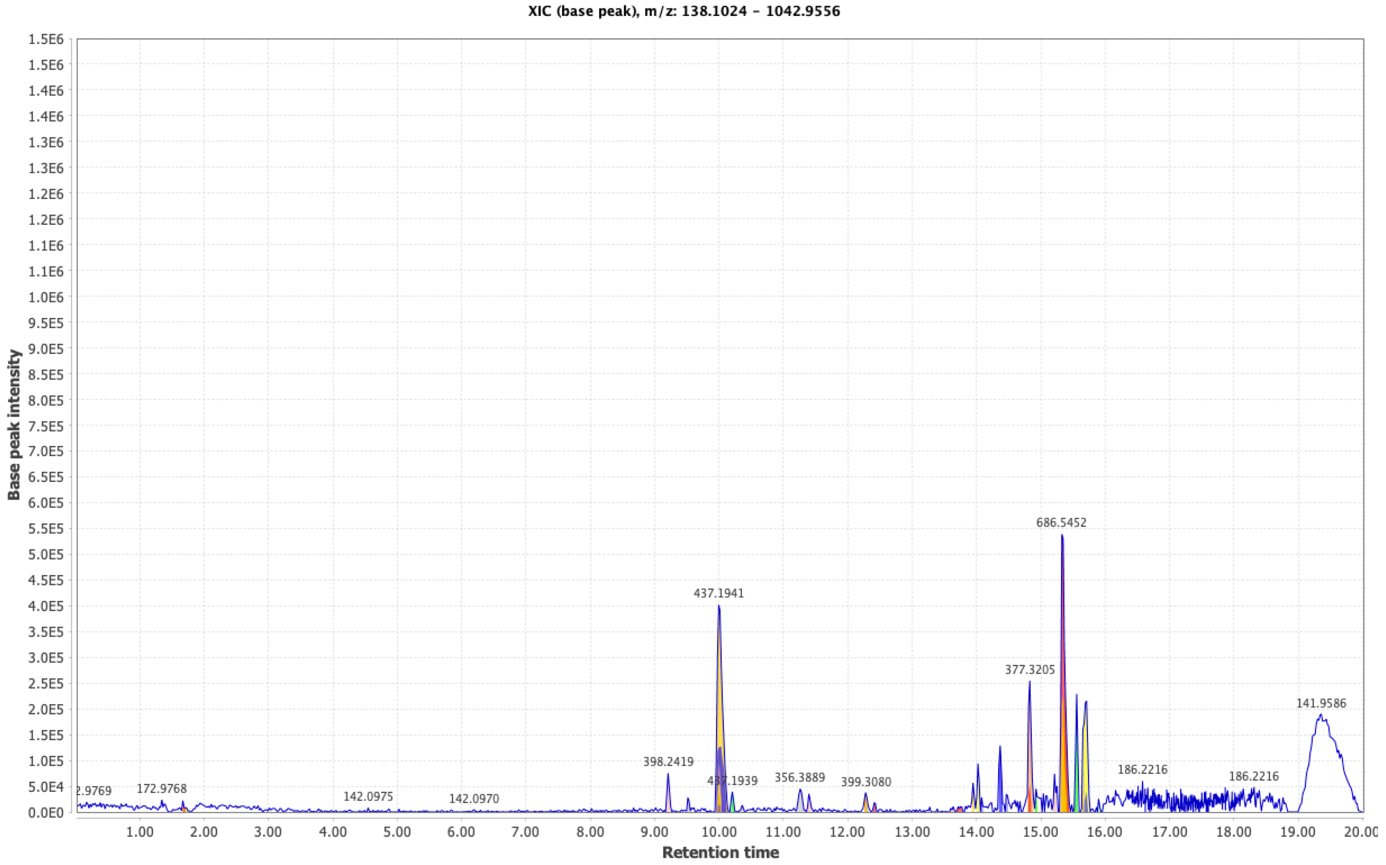


**Figure S37.** Base peak chromatogram (positive ESI) for fungus comb from colony Mn160 extracted in acetonitrile (ACN), 100% acetone fraction (retention time in min).


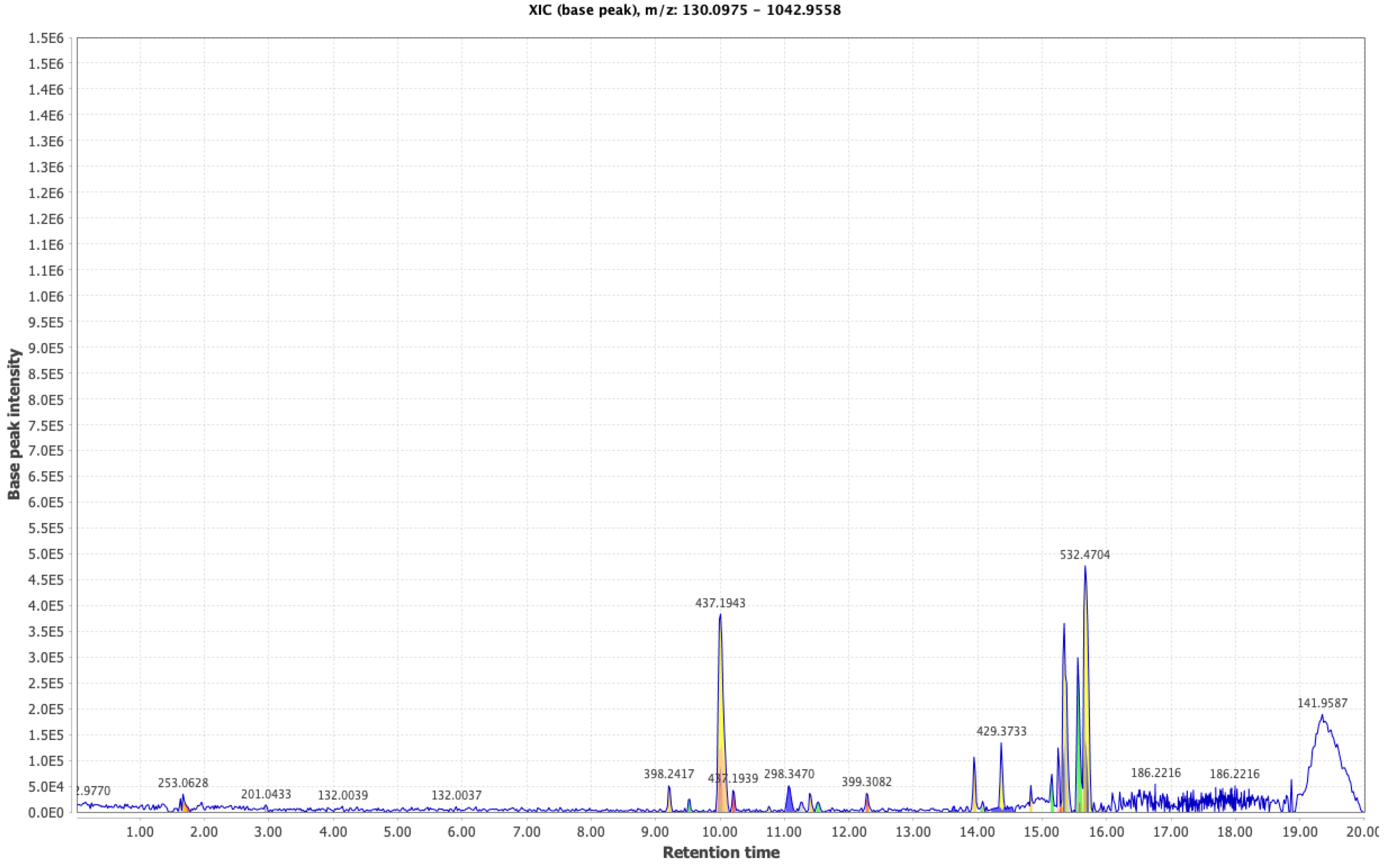


**Figure S38.** Base peak chromatogram (positive ESI) for fungus comb from colony Mn164 extracted in acetonitrile (ACN), 100% acetone fraction (retention time in min).


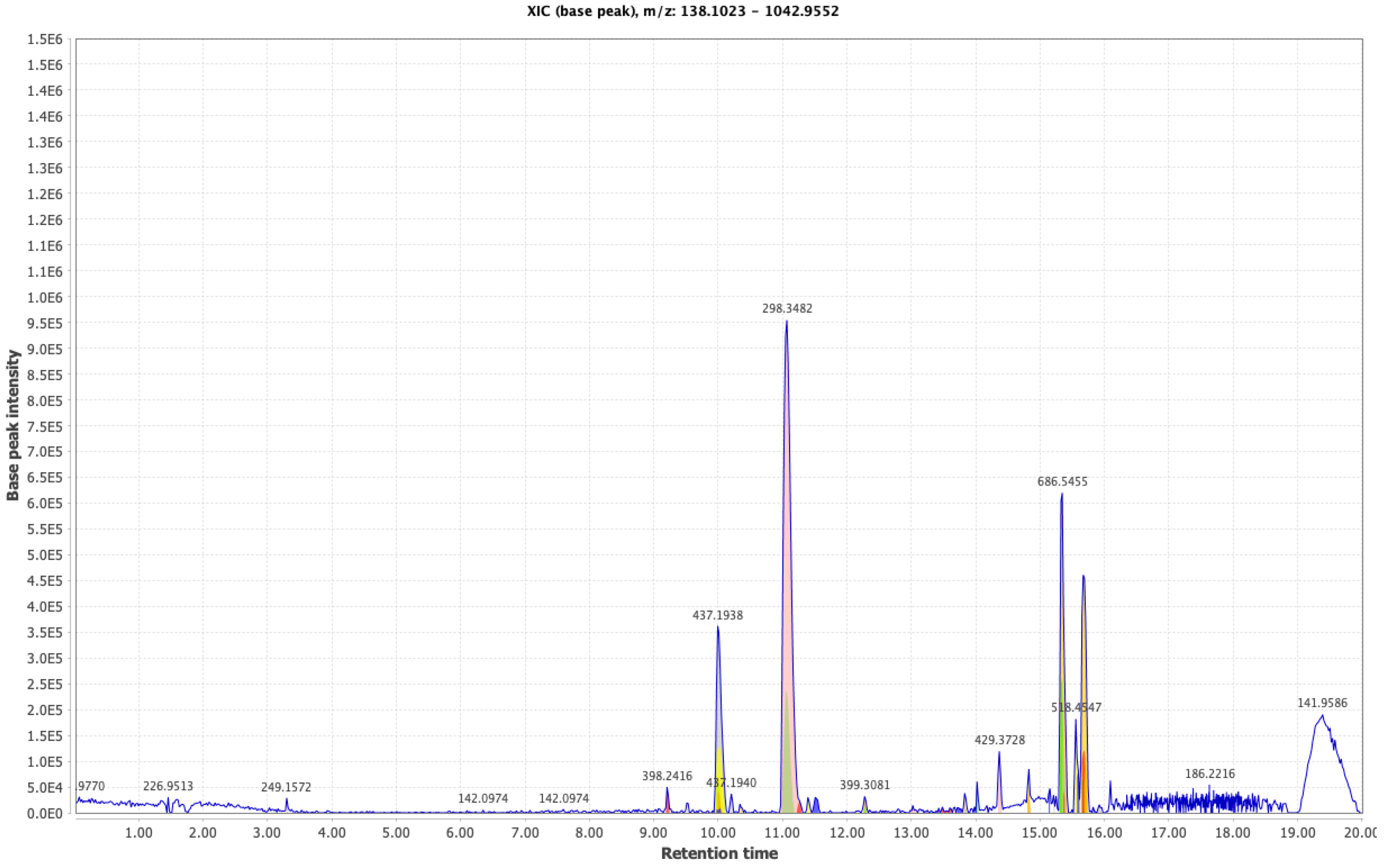


**Figure S39.** Base peak chromatogram (positive ESI) for fungus comb from colony Mn165 extracted in acetonitrile (ACN), 100% acetone fraction (retention time in min).


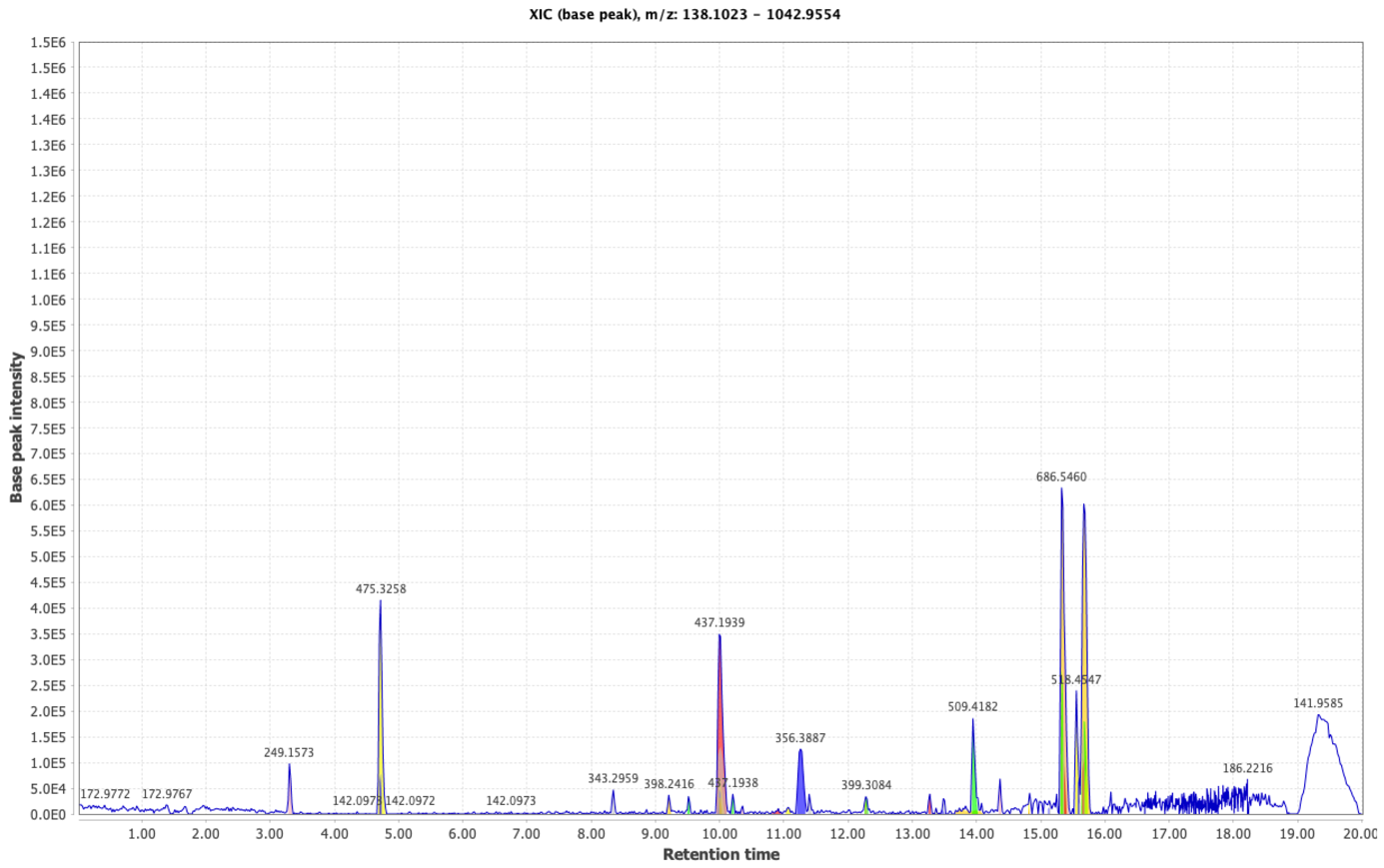


**Figure S40.** Base peak chromatogram (positive ESI) for fungus comb from colony Mn166 extracted in acetonitrile (ACN), 100% acetone fraction (retention time in min).


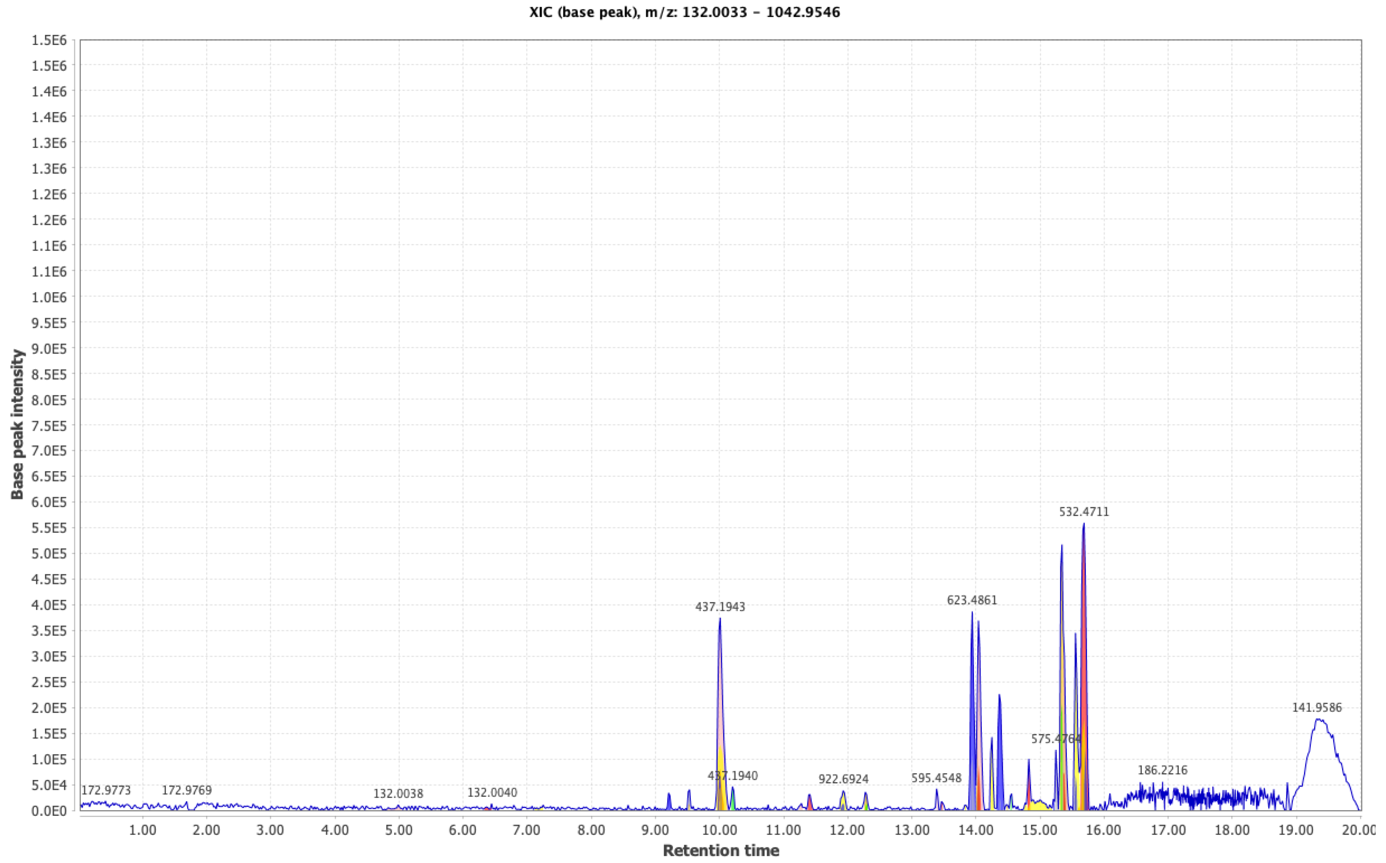


**Figure S41.** Base peak chromatogram (positive ESI) for fungus comb from colony Mn171 extracted in acetonitrile (ACN), 100% acetone fraction (retention time in min).


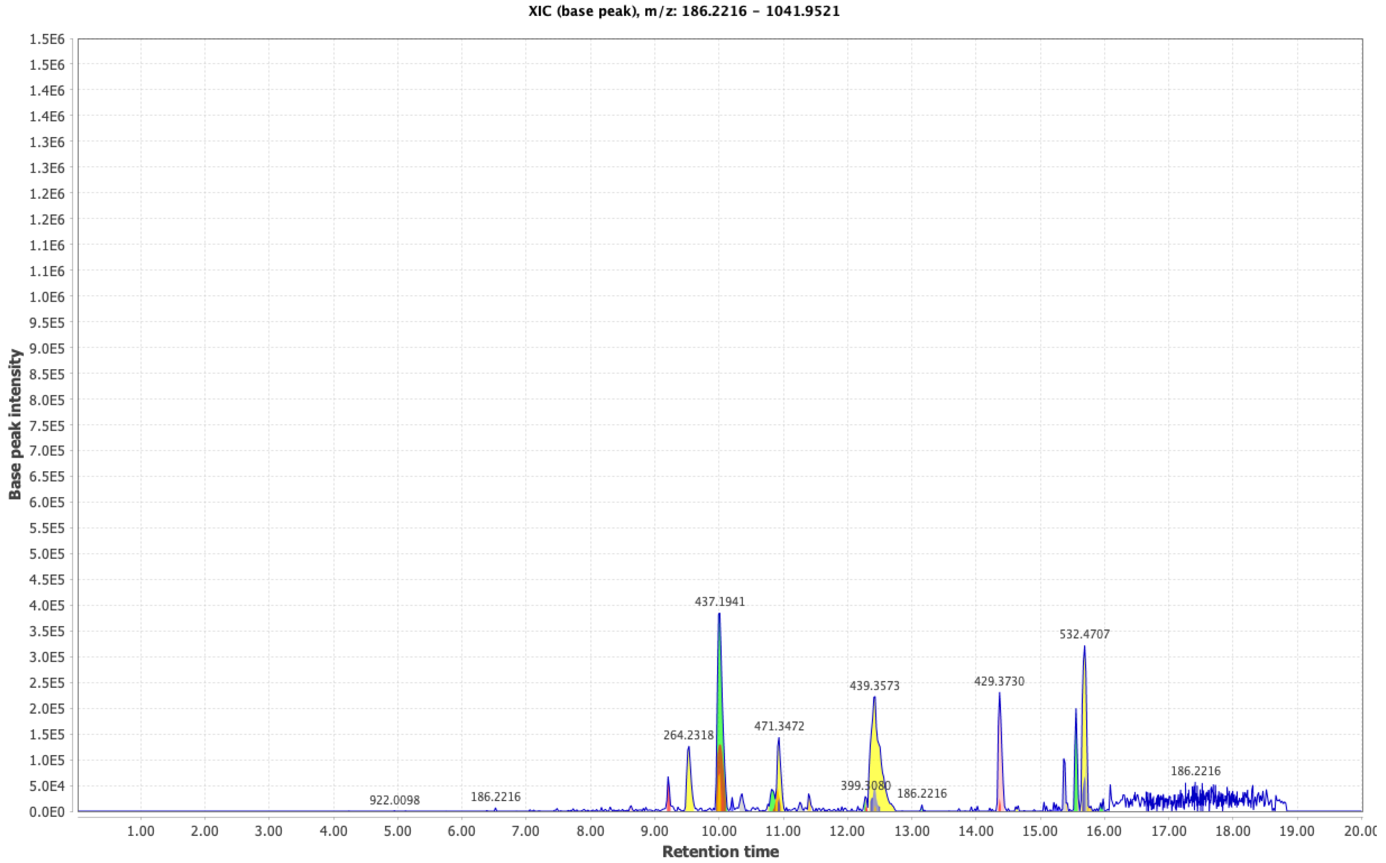


**Figure S42.** Base peak chromatogram (positive ESI) for fungus comb from colony Od127 extracted in acetonitrile (ACN), 100% acetone fraction (retention time in min).


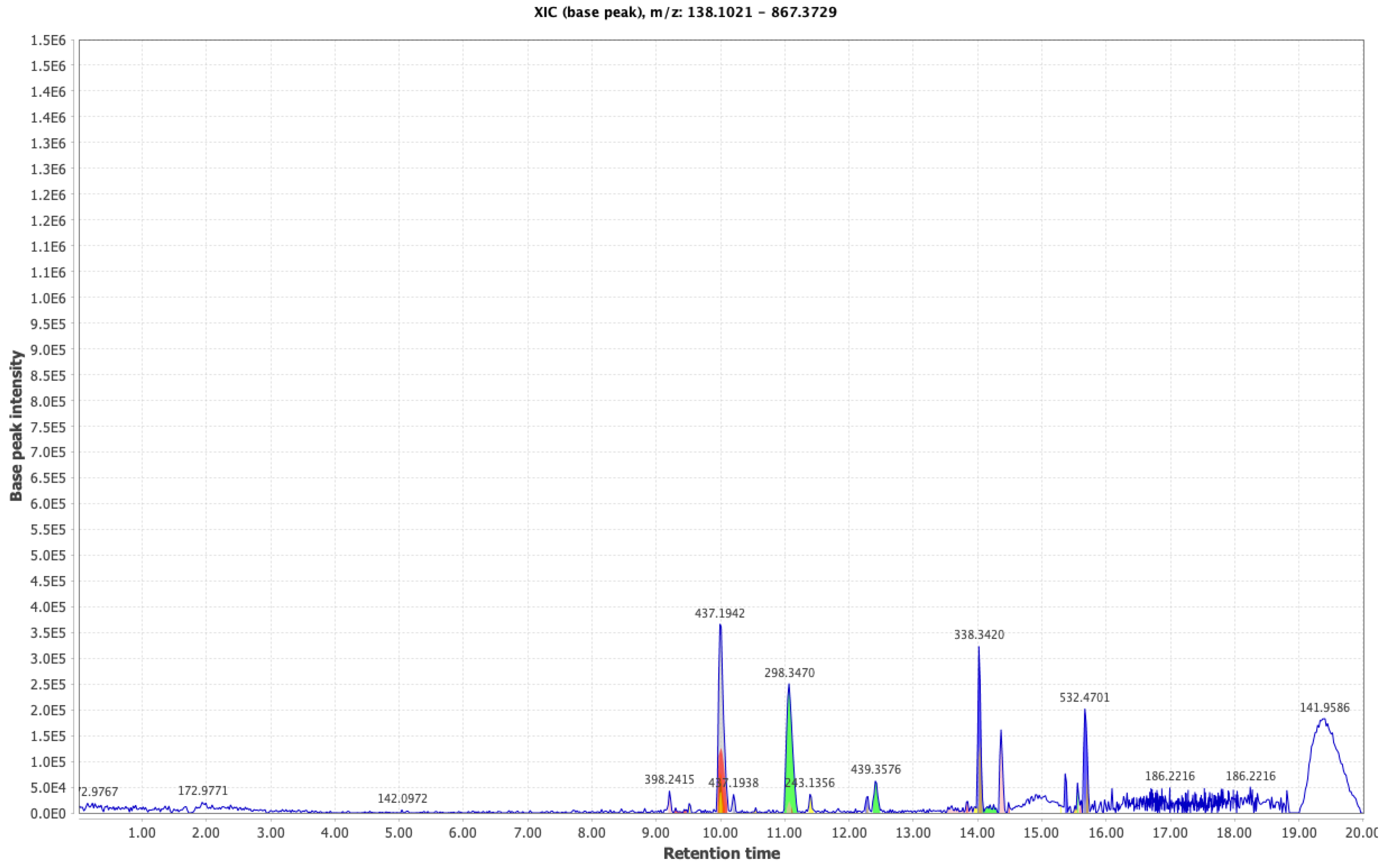


**Figure S43.** Base peak chromatogram (positive ESI) for fungus comb from colony Od152 extracted in acetonitrile (ACN), 100% acetone fraction (retention time in min).


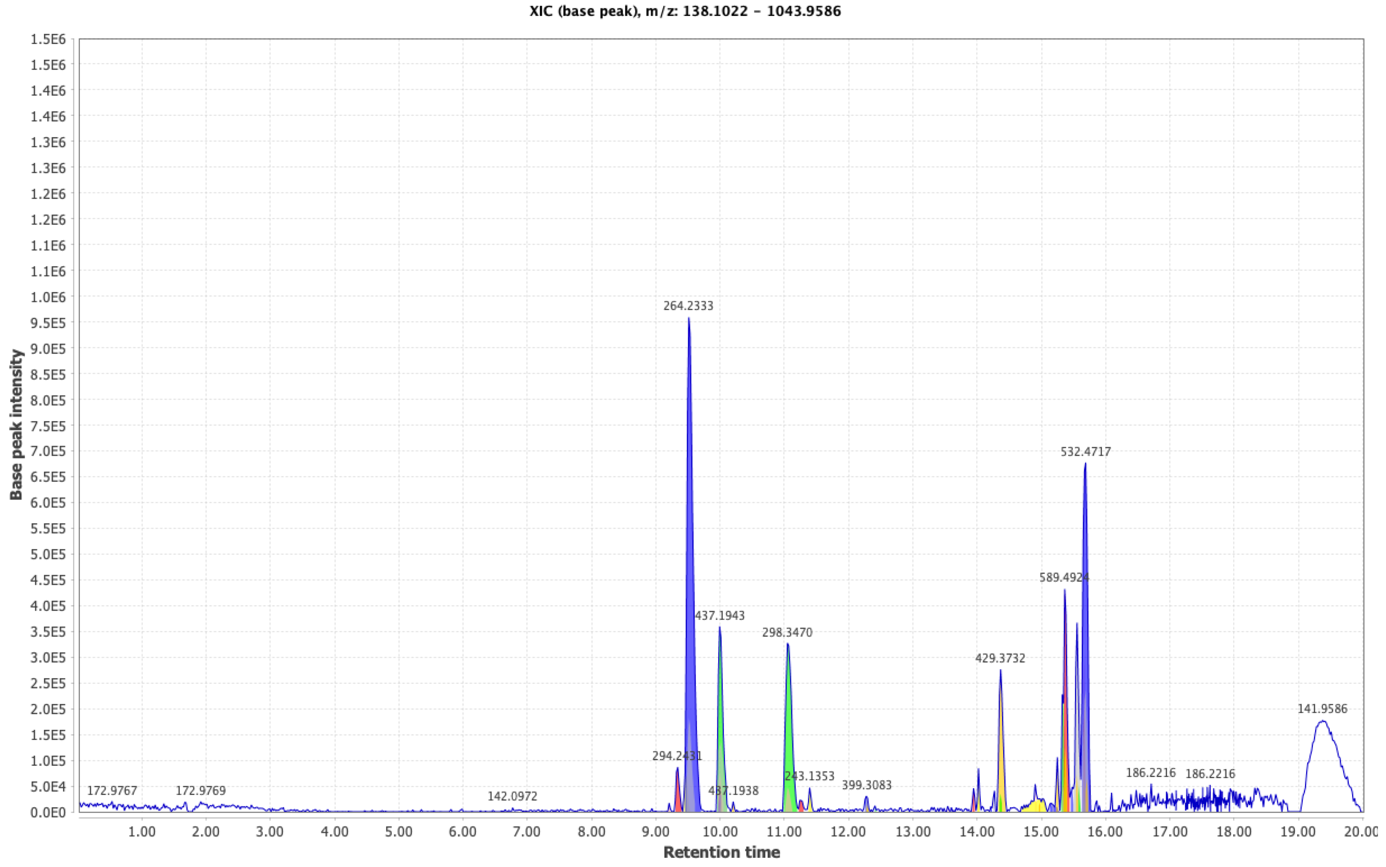


**Figure S44.** Base peak chromatogram (positive ESI) for fungus comb from colony Od167 extracted in acetonitrile (ACN), 100% acetone fraction (retention time in min).


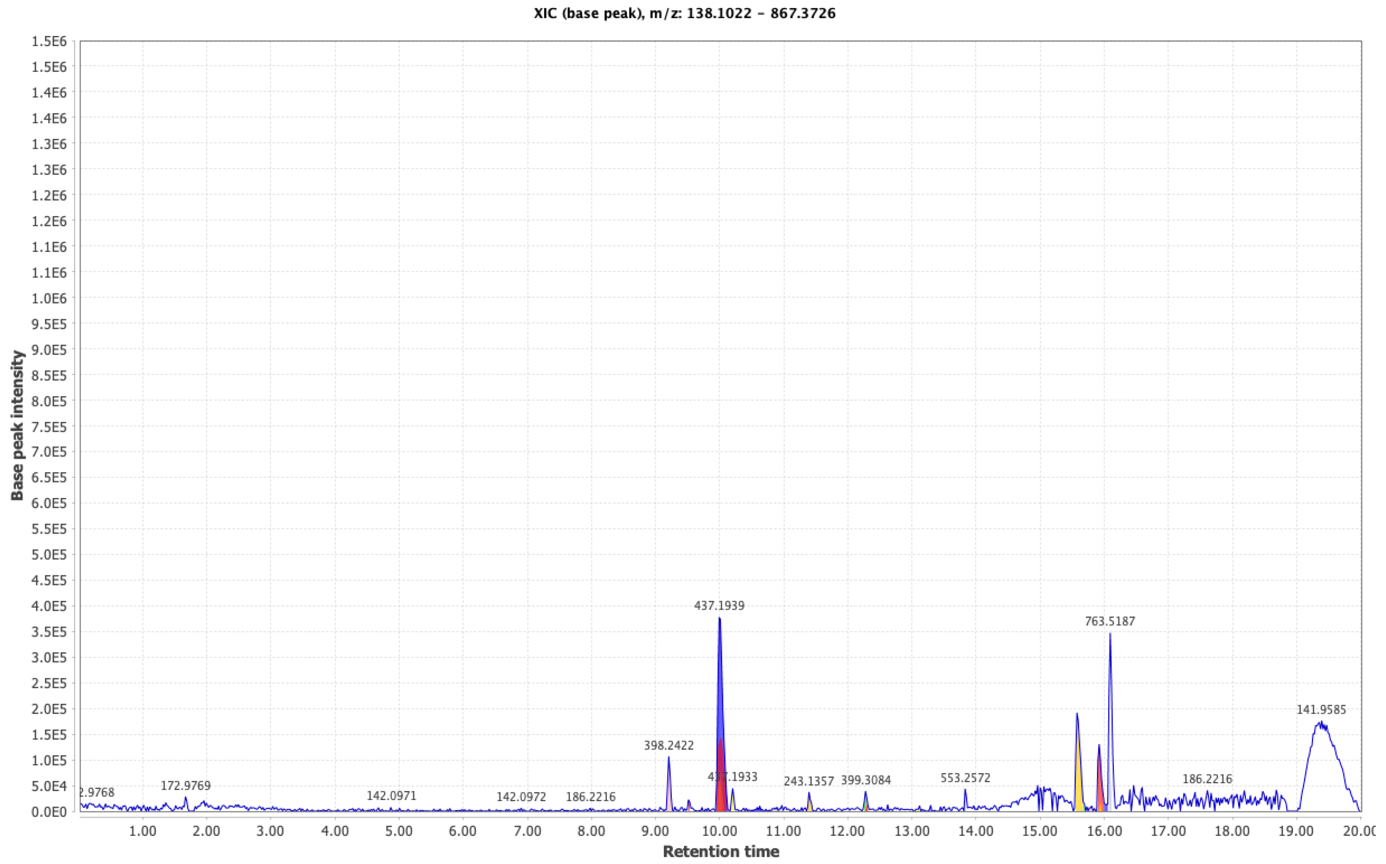


**Figure S45.** Base peak chromatogram (positive ESI) for a blank acetonitrile (ACN) extract, 100% ACN fraction (retention time in min).


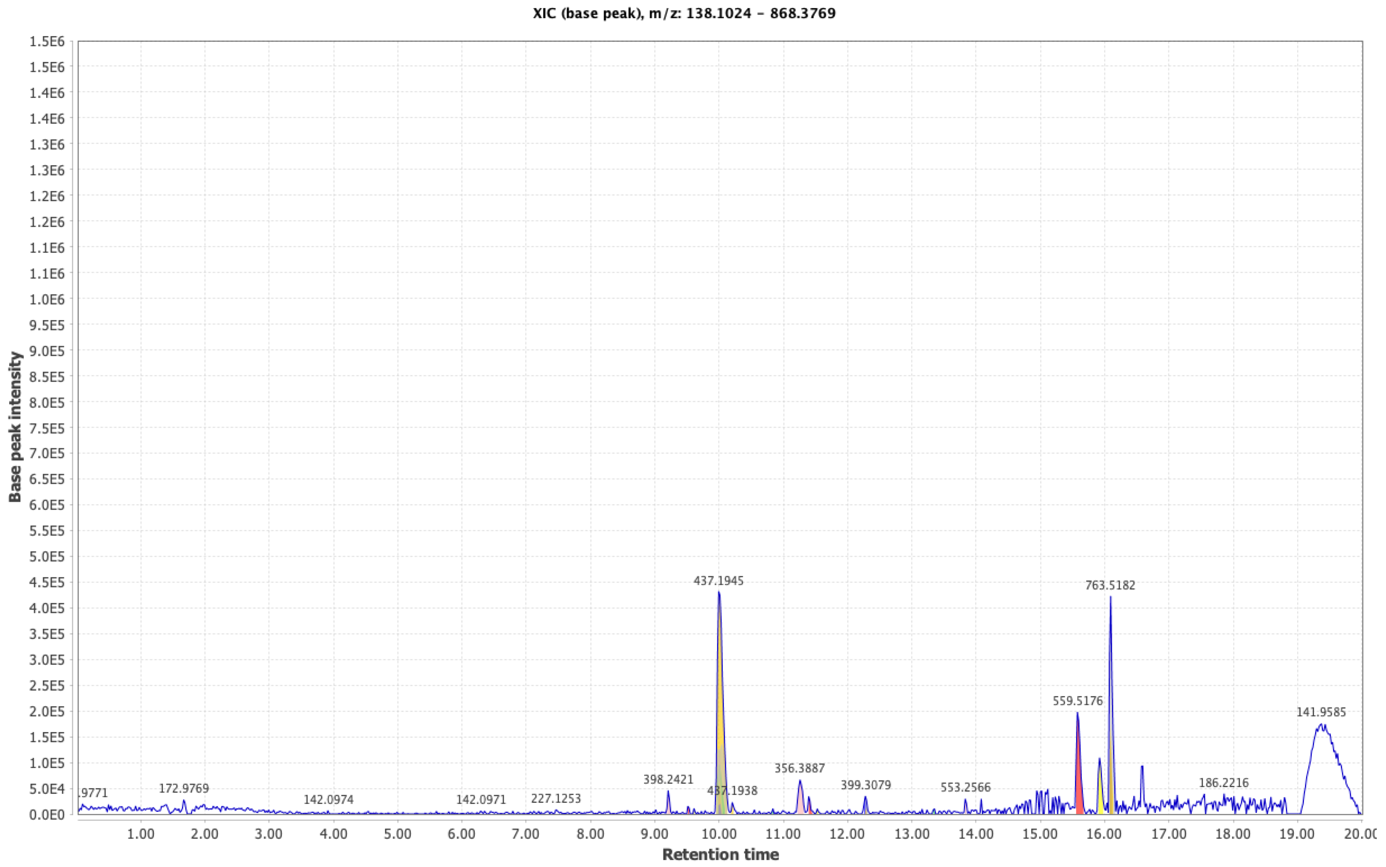


**Figure S46.** Base peak chromatogram (positive ESI) for a blank acetonitrile (ACN) extract, 80% ACN fraction (retention time in min).


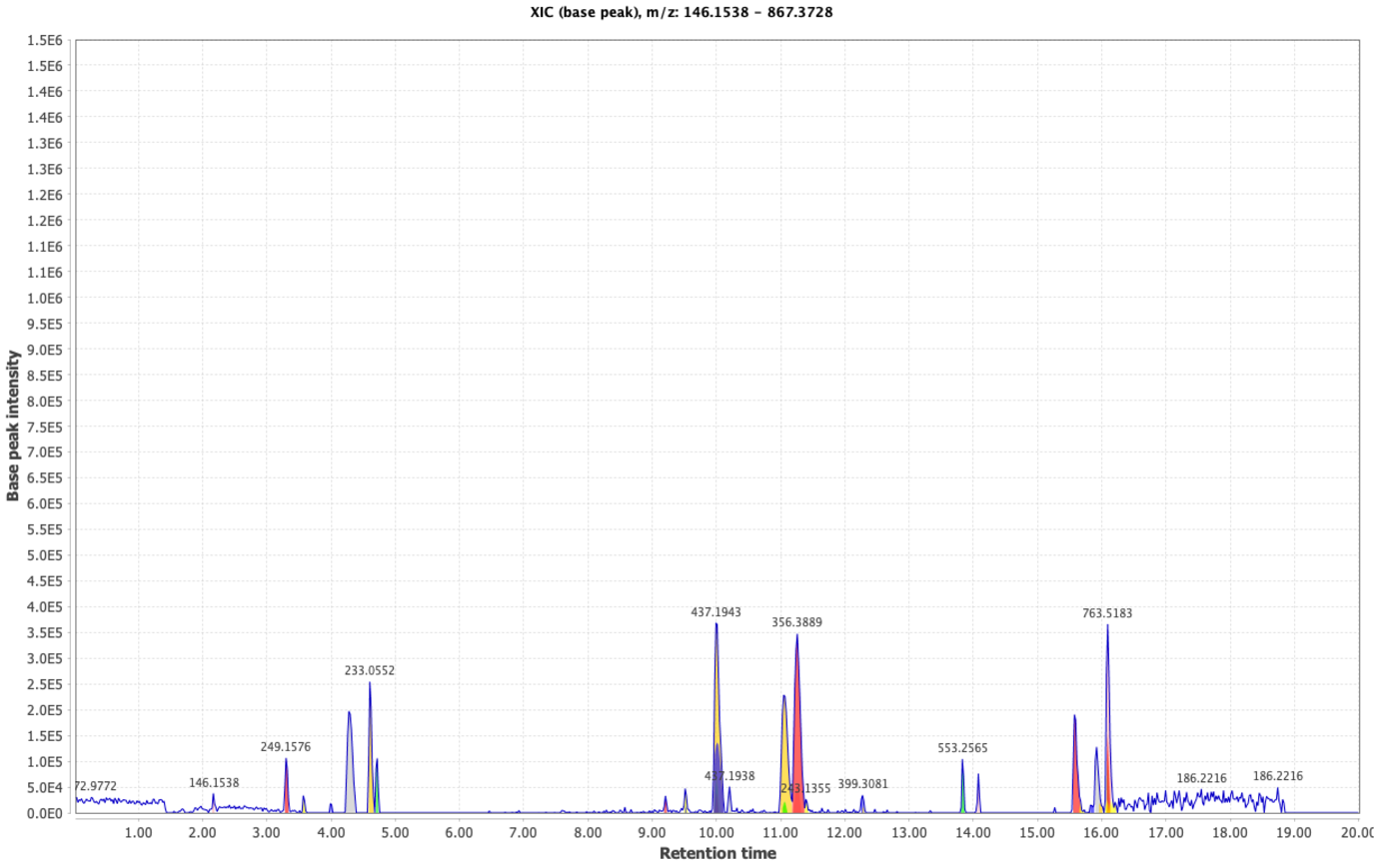


**Figure S47.** Base peak chromatogram (positive ESI) for a blank acetonitrile (ACN) extract, 100% acetone fraction (retention time in min).


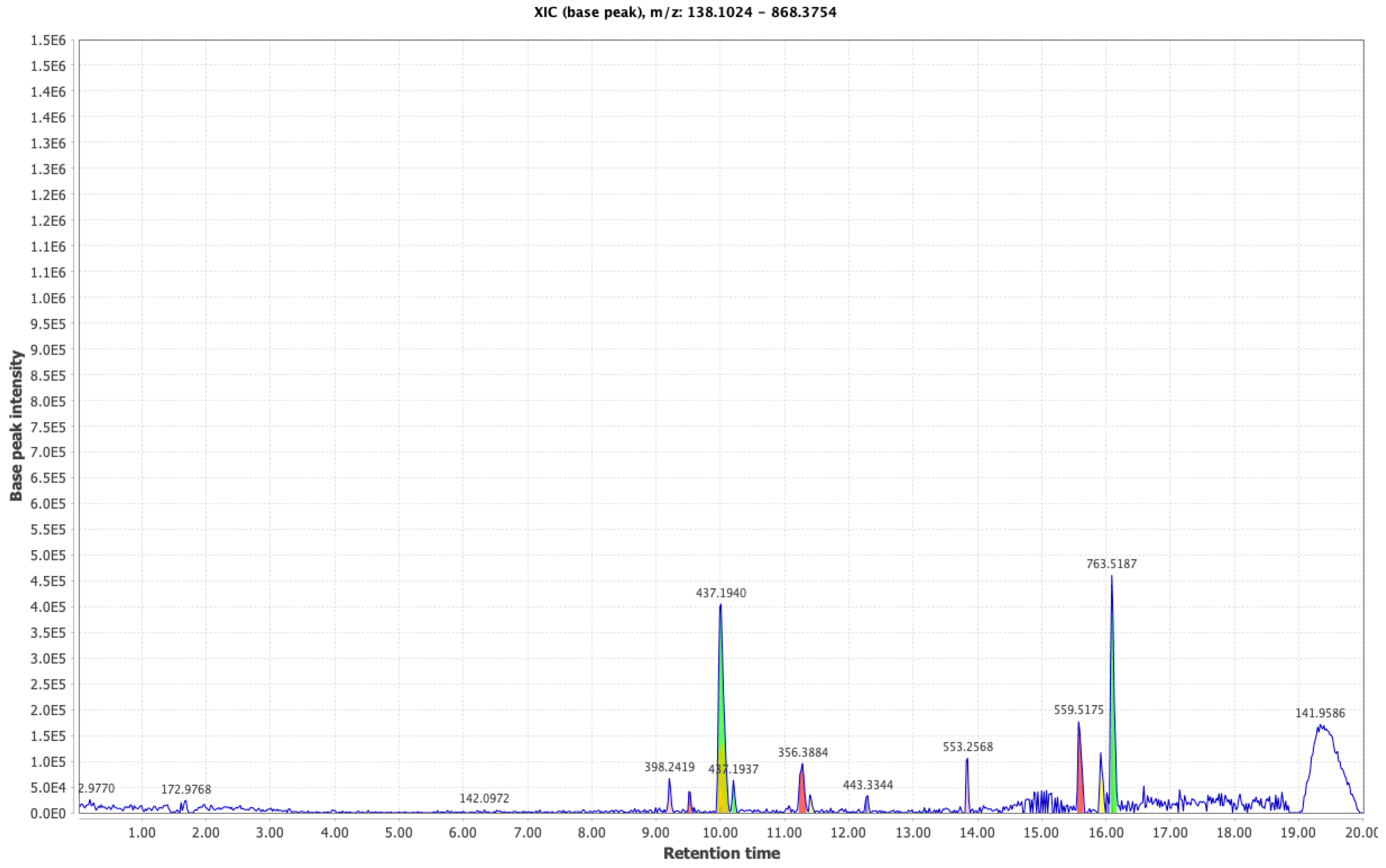


**Figure S48.** Base peak chromatogram (positive ESI) for a blank injection (retention time in min).
